# Accelerated crystal structure prediction of multi-elements random alloy using expandable features

**DOI:** 10.1038/s41598-021-84544-8

**Published:** 2021-03-04

**Authors:** Taewon Jin, Ina Park, Taesu Park, Jaesik Park, Ji Hoon Shim

**Affiliations:** 1 Department of Chemistry, Pohang University of Science and Technology, Pohang, 37673 Republic of Korea; 2 Department of Computer Science and Engineering, Pohang University of Science and Technology, Pohang, 37673 Republic of Korea; 3 Graduate School of Artificial Intelligence, Pohang University of Science and Technology, Pohang, 37673 Republic of Korea; 4 Department of Physics and Division of Advanced Materials Science, Pohang University of Science and Technology, Pohang, 37673 Republic of Korea; 5 Present Address: Department of Chemical and Biomolecular Engineering, Korea Advanced Institute of Science and Technology (KAIST), 291 Daehak-ro, Yuseong-gu, Daejeon, 34141 Republic of Korea

**Keywords:** Materials science, Mathematics and computing, Physics

## Abstract

Properties of solid-state materials depend on their crystal structures. In solid solution high entropy alloy (HEA), its mechanical properties such as strength and ductility depend on its phase. Therefore, the crystal structure prediction should be preceded to find new functional materials. Recently, the machine learning-based approach has been successfully applied to the prediction of structural phases. However, since about 80% of the data set is used as a training set in machine learning, it is well known that it requires vast cost for preparing a dataset of multi-element alloy as training. In this work, we develop an efficient approach to predicting the multi-element alloys' structural phases without preparing a large scale of the training dataset. We demonstrate that our method trained from binary alloy dataset can be applied to the multi-element alloys' crystal structure prediction by designing a transformation module from raw features to expandable form. Surprisingly, without involving the multi-element alloys in the training process, we obtain an accuracy, 80.56% for the phase of the multi-element alloy and 84.20% accuracy for the phase of HEA. It is comparable with the previous machine learning results. Besides, our approach saves at least three orders of magnitude computational cost for HEA by employing expandable features. We suggest that this accelerated approach can be applied to predicting various structural properties of multi-elements alloys that do not exist in the current structural database.

## Introduction

Properties of solid-state materials are strongly related to their crystal structures. Even in the same elemental composition, the physical properties such as magnetization and adsorption energy are significantly affected by the crystal structures^1–4^. HEA, which consists of more than five elements, has drawn intensive attention for its outstanding mechanical properties^5,6^ when forming the solid solution phase. The mechanical properties of solid solution HEA depend on its phase. The *fcc* HEA has high ductility^7^, and the *bcc* HEA has high strength^8^. That`s why valence electron concentration (VEC) is used to classify the *bcc* and the *fcc* solid solution phase of HEA^9,10^.

To confirm the crystal structures efficiently, the structural searching in combination with the evolutionary algorithm with density functional theory (DFT) have been applied^11,12^. Recent approaches for crystal structure prediction become accelerated by adopting machine learning algorithms trained with the available experimental and theoretical database. Learning-based methods even predict the crystal structures of unknown materials using a sufficient number of training data^13,14^. As a result, one can bypass direct experiments or calculations to find the structural phases, so the cost for exploring the unknown materials and their characteristics becomes significantly reduced. In practice, the existing database, such as the inorganic crystal structure database (ICSD)^15^ and Automatic-Flow (AFLOW)^16^ have been used for training data. For instance, to investigate the most probable Mn-Ge and Li-Mn-Ge system structure, deep neural network (DNN) with ICSD has been used to predict the crystal structures^13^. When the number of the existing training data is insufficient, the calculation based on DFT can be applied to generate the training data^17^.

However, the above approaches cannot be applied to the unexplored multi-elements alloys such as HEA^18^ because of the insufficient data in the experiment. In addition, the possible compositional number of HEA is more than 10^6^, so preparing training data set of HEA using DFT calculation like other crystal system^19,20^ is infeasible. Although some machine learning-based approach shows accurate performance ^21,22^, the most approaches for predicting phases of unexplored HEA are restricted to nearly equiatomic cases^23,24^. It is because the calculation of the non-equiatomic HEA dataset requires huge computation due to its vast compositional space^25^. Therefore, the prediction of the HEA’s crystal structures without the calculation in the vast space is a demanding issue^26^.

In this sense, we develop a learning-based approach to predict the vast compositional space of multi-element alloys (binary alloy, ternary alloy, and HEA), while only the binary alloy dataset is involved as the training set.

For structural phase prediction using a learning-based approach, designing proper features is crucial, because it determines the cost and accuracy of the prediction. Conventionally, the compositional properties such as *Z*^(*i*)^ (atomic number), $n_{d}$^(*i*)^ (*d*-orbital occupancy), and $\sigma_{d}$^(*i*)^ (*d*-orbital spin) for *i*th atom are used as proper features for predicting structural phases of binary alloys^27^.

Especially in previous works, it is revealed that $n_{d}$ and $\sigma_{d}$ denotes occupancy of *d* electrons^30^. The *d* electron occupancy effectively involves in cohesive interaction and determines the stability of the crystal structural phase. Therefore, from several decades ago, this occupancy is widely used to classify the structural phase of transition metal. H. L. Skriver classify *bcc*, *fcc,* and *hcp* phase of 3*d*, 4*d* and 5*d* non-magnetic transition metal using $n_{d}$^28^, and it is expanded to magnetic transition metal using $n_{d}$ and $\sigma_{d}$ features^29,30^.

However, this approach is not directly applicable to multi-element alloys because the number of features is increasing as the types of elements increase. Although {$n_{d}$^(*N*)^, $\sigma_{d}$^(*N*)^}, as a list of paired features for *N*-elements alloy, are well known as features for the crystal structure prediction of transition metal^30^, expensive DFT calculation is still necessary to obtain those values for multi-element alloys.

**Figure 1:**
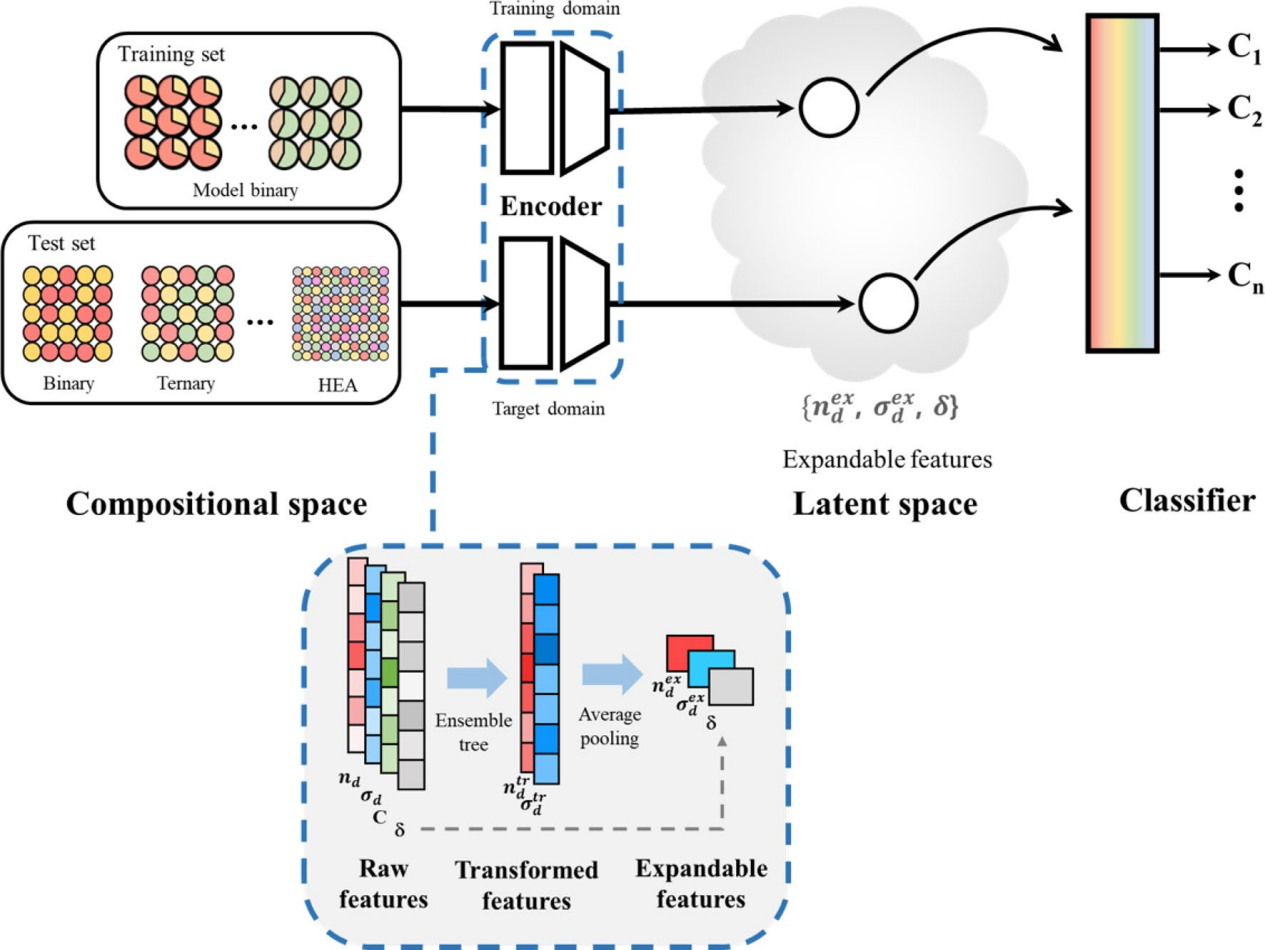
Schematic representation for feature transformation module to obtain {$n_{d}^{ex}$, $\sigma_{d}^{ex}$} features from {$n_{d}$^(*N*)^, $\sigma_{d}$^(*N*)^} features. {$n_{d}$^(*N*)^, $\sigma_{d}$^(*N*)^, *C*, δ} features in alloy {$M$^(*N*)^} consist of raw features. With regression tree ensembles, {$n_{d}$^(*N*)^, $\sigma_{d}$^(*N*)^} features are transformed to {$n_{d}$ ^tr (*N*)^, $\sigma_{d}$ ^tr (*N*)^}. In this transformation, {*C*, δ} features are used in edges in the ensemble tree. Then, by vector-wise average pooling, {$n_{d}$ ^tr (*N*)^, $\sigma_{d}$ ^tr (*N*)^} is reduced to {$n_{d}^{ex}$, $\sigma_{d}^{ex}$} features, which used *C* of the constituent atom as weight. {$n_{d}^{ex}$, $\sigma_{d}^{ex}$} is used for the training of the calculated binary alloy dataset. Note that {$n_{d}$^(*N*)^, $\sigma_{d}$^(*N*)^} features are the information from a pure transition metal, while {$n_{d}^{ex}$, $\sigma_{d}^{ex}$} features represent the information in alloy condition. After the training of the module, the {$n_{d}^{ex}$, $\sigma_{d}^{ex}$} features obtained from multi-element alloys can be used for the prediction of the structural phases.

In this work, we propose expandable {$n_{d}^{ex}$, $\sigma_{d}^{ex}$} features, which are transformed from {$n_{d}$^(N)^, $\sigma_{d}$^(N)^} features as illustrated in Fig. 1. For the transformation from {$n_{d}$^(N)^, $\sigma_{d}$^(N)^} to {$n_{d}^{ex}$, $\sigma_{d}^{ex}$}, we utilized ensemble trees^31^ considering each atoms’ surrounding condition in the alloy. In practice, $n_{d}$ and $\sigma_{d}$ of the transition metal can be changed by the electron transfer from *s* or *p* orbitals when the lattice constants or surrounding atoms are changed in transition metal alloy^32^. For example, $\sigma_{d}$ of Mn can be significantly enlarged when the volume of the alloy increases^33^. To consider those conditions, the concentration (*C*) and atomic radius difference (*δ*) are added as additional features to obtain {$n_{d}^{tr \left( N \right)}$, $\sigma_{d}^{tr \left( N \right)} \}$ features in alloy condition. Finally, the {$n_{d}^{tr \left( N \right)}$, $\sigma_{d}^{tr \left( N \right)} \}$ features are reduced to {$n_{d}^{ex}$, $\sigma_{d}^{ex}$} features by average pooling, as shown in Fig. 1. (The details of the feature transformation process are in section "Algorithm of this work".) Note the {$n_{d}^{ex}$, $\sigma_{d}^{ex}$} features are always two variables in any number of element types in the multi-elements alloy. So, these expandable features can be used to train of binary alloy dataset, and then applied to the prediction of the multi-elements alloy properties as demonstrated in the following.

## Results and discussions

For the generation of binary alloy dataset, the stable crystal structures of disordered transition metal binary alloys are calculated in all compositional space. Using DFT calculations, we consider three structural phases of body-centered cubic (*bcc*), face-centered cubic (*fcc*), and hexagonal close-packed (*hcp*), which are competing with each other depending on the elemental configurations. The calculated structural phases are compared to the experimental results, which show good agreement. So, we believe that our calculated results can be used for the training set of crystal structure prediction of the experimental data.

**Figure 2:**
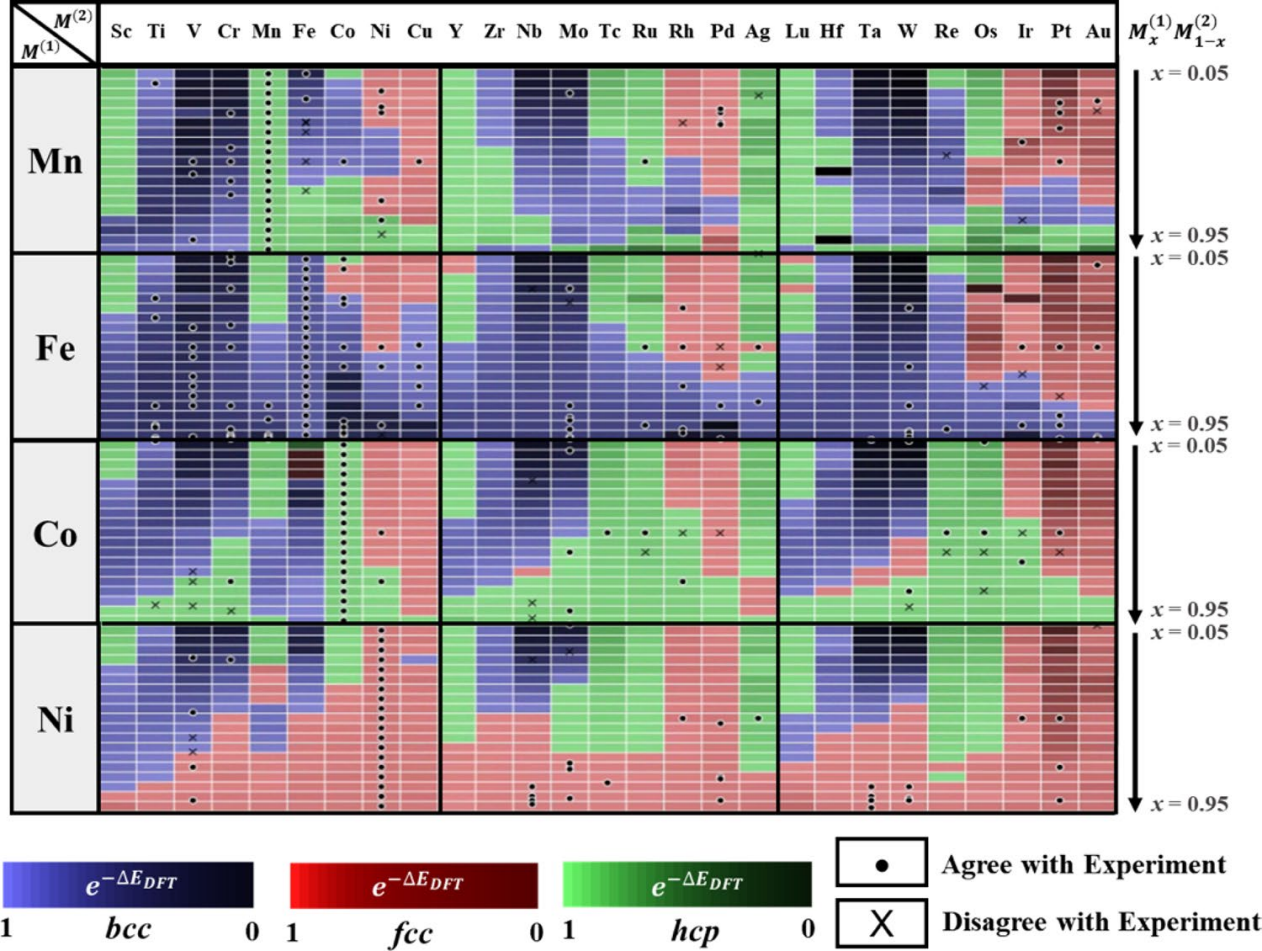
Binary alloy dataset of $M_{x}^{\left( 1 \right)} M_{1 - x}^{\left( 2 \right)}$($M^{\left( 1 \right)}$ = Mn, Fe, Co, and Ni; $M^{\left( 2 \right)}$ = TM) generated from DFT calculation. *bcc*, *fcc*, and *hcp* structures are denoted in blue, red, and green, respectively. The lowest energy phase is denoted as a stable phase and has the second-lowest energy used as a meta-stable phase. The energy difference between stable phase and meta-stable phase is denoted as ΔE_DFT_. To confirm the validity of the DFT calculation, the phases of binary alloys from experimental reports are denoted as dot and cross for correct and incorrect predictions, respectively. Among the experimental data of binary alloys, 212 alloys containing Mn, Fe, Co, and Ni are used (The experimental binary alloy data is available in Table S1).

**Figure 3:**
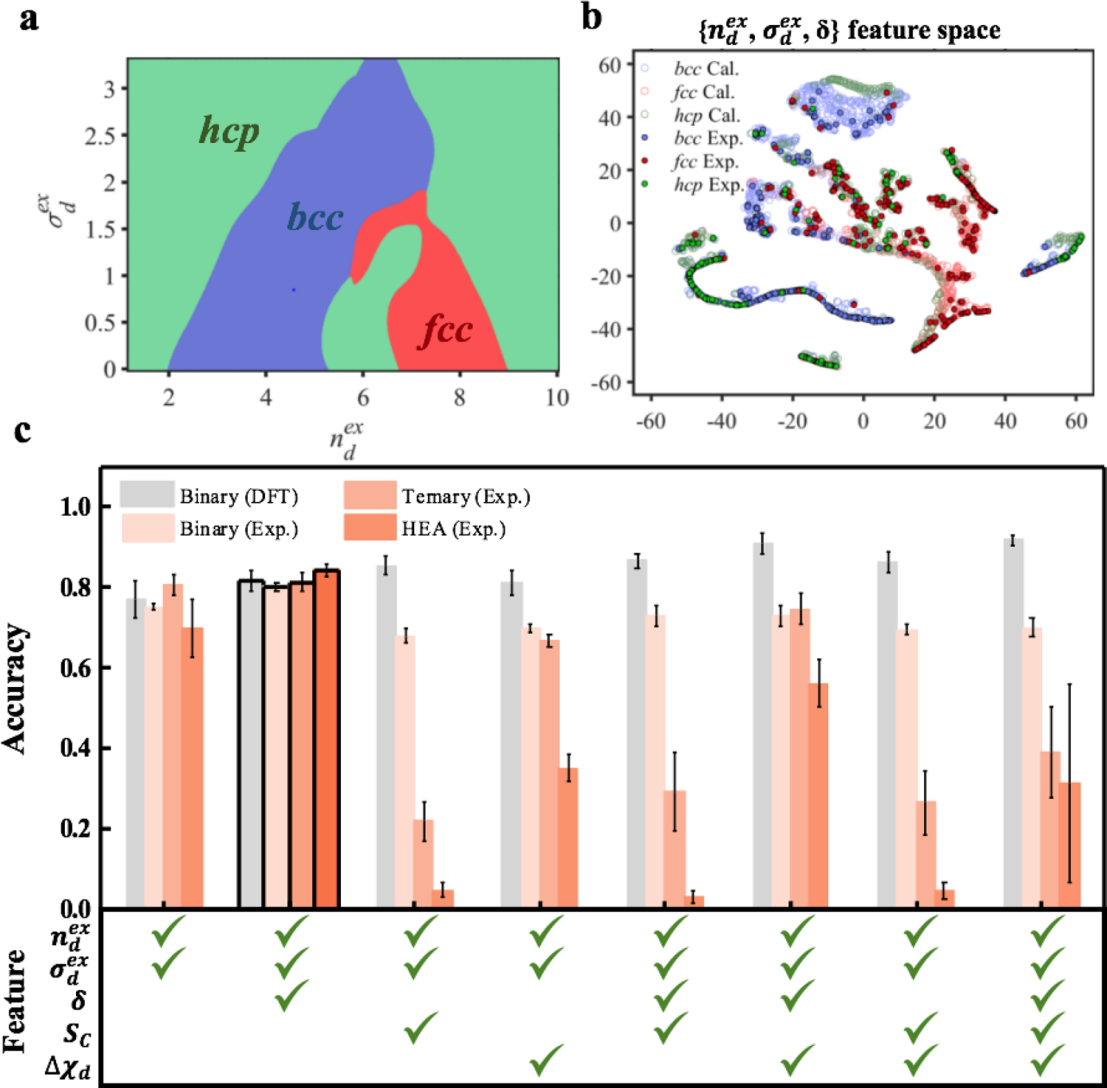
(**a**) Classification region of the structural phases in {$n_{d}^{ex}$, $\sigma_{d}^{ex}$} feature space, which is trained from the calculated binary alloy dataset. *bcc*, *fcc,* and *hcp* are denoted with blue, red, and green shaded regions, respectively. Since the trained regions from the {$n_{d}^{ex}$, $\sigma_{d}^{ex}$} and {$n_{d}^{\left( N \right)}$,${ }\sigma_{d}^{\left( N \right)}$} features are similar, and we only show the trained result from {$n_{d}^{ex}$, $\sigma_{d}^{ex}$} features in this figure. (**b**) A t-SNE^58^ plot of {$n_{d}^{ex}$, $\sigma_{d}^{ex}$, *δ*} features. The 1862 binary alloys from DFT calculation and the experimentally determined 870 multi-element alloys are denoted with circles for comparison. All the experimental multi-element alloys are located in the range of the calculated binary alloys. (**c**) Mean accuracy of the test set for the calculated data of binary alloy and the experimental data of binary alloy, ternary alloy, and HEA with various feature sets. 10% of the calculation data is randomly chosen as the test set, and the remaining 90% of the calculated data is used as the training set. The error bar denoted the standard deviation of the accuracy.

To validate the {$n_{d}^{ex}$, $\sigma_{d}^{ex}$} features, we compare the accuracies of structural phase predictions using {$n_{d}^{\left( N \right)}$, $\sigma_{d}^{\left( N \right)}$} and {$n_{d}^{ex}$, $\sigma_{d}^{ex}$} features by the evaluation from the test set of the calculated binary alloys in Fig. 2. Figure 3(a) shows structural phase classification region trained by {$n_{d}^{ex}$, $\sigma_{d}^{ex}$} features. The accuracy with {$n_{d}^{\left( N \right)}$, $\sigma_{d}^{\left( N \right)}$} features is 81.1% and with {$n_{d}^{ex}$, $\sigma_{d}^{ex}$} features is 78.74%. This validation indicates that the transformed features, {$n_{d}^{ex}$, $\sigma_{d}^{ex}$} well reproduce the prediction accuracy with raw features, {$n_{d}$^(N)^, $\sigma_{d}$^(N)^} in the binary alloy.

In addition to the transformed {$n_{d}^{ex}$, $\sigma_{d}^{ex}$} features, we also use atomic size difference (*δ*), configurational entropy (*S*_*c*_) and electronegativity difference ($\chi$_*d*_) which are known to determine the stability of HEA^49^. To predict the structural phase with the chosen features, the support vector machine^50^ was used with the calculated binary alloy dataset in Fig. 2. Figure 3(c) shows the accuracies of the phase prediction for each set of chosen features. As expected, the accuracy for binary dataset increases with a large number of features, and it is up to 91.78%. This behavior is well known and shown in most machine learning works^22,23^ when the training set and test set are divided from the same data set.

We applied this trained algorithm to the experimentally reported multi-elements alloys (Tables S1 and S2) for the practical demonstration of this work. Here, many binary alloys, ternary alloys, and HEAs such as VNbMoTaW^51^ and CoCrFeMnNi^52^, well known for application, are included in the test set. As shown in Fig. 3(c), the accuracies of the calculated data and all the experimental data are comparable in case of {$n_{d}^{ex}$, $\sigma_{d}^{ex}$} and {$n_{d}^{ex}$, $\sigma_{d}^{ex}$, *δ*} feature spaces. In {$n_{d}^{ex}$, $\sigma_{d}^{ex}$, *δ*} feature space as shown in Fig. 3(b), the accuracy for experimental data of all multi-elements alloys is 80.56%, which is comparable with 81.79% accuracy for calculated data of binary alloys. Especially, the accuracy of the HEA increases up to 84.20%, and it comes from that the existing HEA data mainly consists of *bcc* and *fcc* phases. In both the calculated data and the experimental data, the misclassification data mainly comes from *fcc* and *hcp* phases (See the confusion matrices in figure S3). It implies that the accurate determination of *fcc* and *hcp* phases in the calculated data will improve the classification performance of the experimental multi-element alloy.

This result implies that the trained algorithm by binary alloys can be expanded to the prediction for the experimental multi-elements dataset, including HEA. The accuracy of HEA is comparable with the previous works that classify the phases of HEA with machine learning. For classification of *bcc*, *fcc,* and NSP (non-single phase) of HEA with support vector machine (SVM), it has 90.69% accuracy^22^ and classification of *bcc*, *fcc,* and *hcp* phase of HEA 87 ~ 89% accuracy^23^.

**Figure 4:**
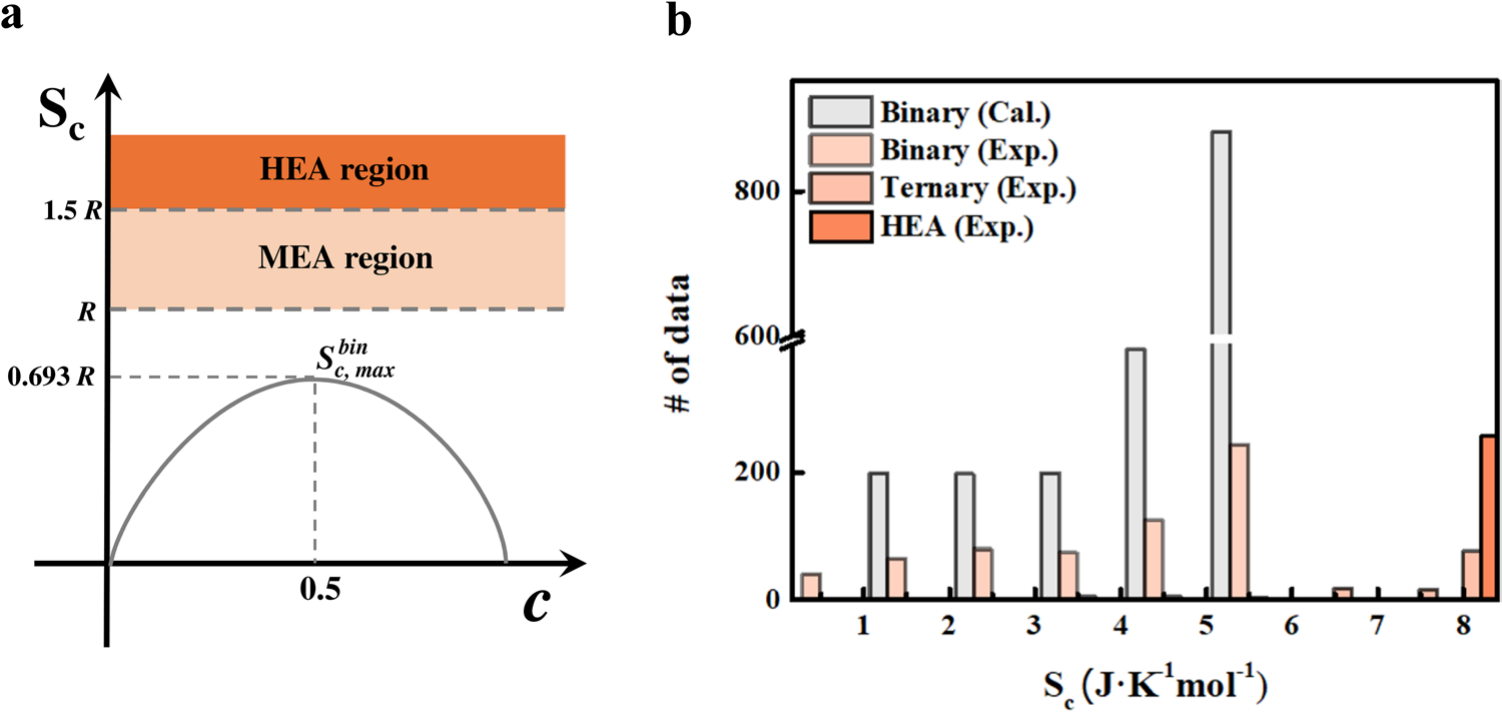
(**a**) S_c_ of binary alloy as a function of *c* (Gray line). $S_{c,max}^{bin}$ is the theoretical maximum S_c_ that binary alloy can have. Beige color and orange color denote the MEA (Medium entropy alloy) and HEA region, respectively. (**b**) Data distribution of calculated binary alloy and experimental data (binary alloy, ternary alloy, HEA) in and S_c_ feature.

Unlike the accuracy of the calculated binary alloy data, the accuracy of the experimental data of ternary alloy, and HEA drastically decreases when S_c_ and $\chi$_*d*_ features are added. Figure 4(a) shows why S_c_ can`t use as expandable features. In Fig. 4(a), binary can have $S_{c,max}^{bin}$ when it forms the binary equiatomic alloy. However, a multi-element alloy which consists of MEA and HEA have larger S_c_ than $S_{c,\max }^{{{\text{bin}}}}$ ^53^. It makes the HEA data located in the extrapolative region. In Fig. 4 (b), most S_c_ of ternary alloy and HEA data distributed out of range of the binary alloy data. It is well known that machine learning shows poor performance in extrapolation region^54^, so the HEA data shows 30% accuracy with the five features, including S_c_. When $\chi$_*d*_ is applied as an additional feature, the accuracy of HEA also significantly decreases compared with the binary and ternary alloy, so the $\chi$_*d*_ feature is not considered as an expandable feature for phase prediction of HEA.

This work has an advantage of saving costs for generating training set data. The existing work based on raw features and neural networks used at least 80% data of HEA in the training process to classify the phases of HEA^23,55,56^. The HEA data for training is limited because it is based on the experimental results, and it is hard to get calculation data for its substantial computational cost. However, in this work, no HEA data is involved in the training process. Instead, a simple binary alloy dataset is used to predict the phase of HEA with comparable accuracy. This kind of approach is not common and applicable only when the training set and the test set are located in similar feature space (Fig. 3b).

**Figure 5:**
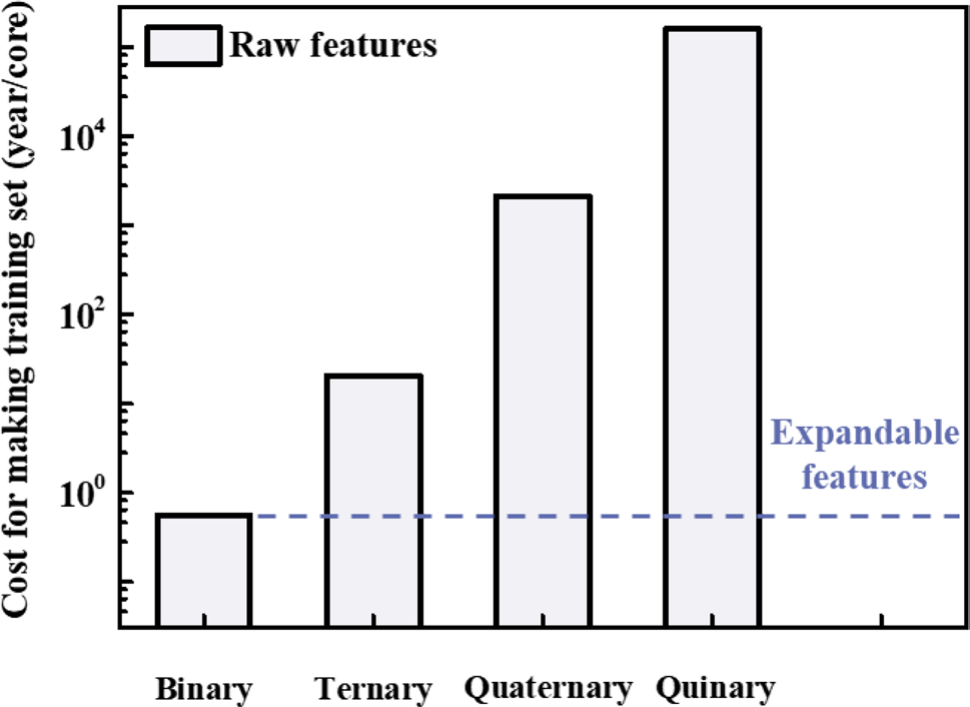
Computational cost of generating a training dataset using DFT calculation. For the training set using raw features, we suppose that 80% of the training set is required from the original dataset. However, using expandable features, the cost for generating the training set of the binary alloy is only required because the training algorithm using expandable features can be directly applicable to the multi-element alloys.

By using expandable features for the structural phase prediction of multi-elements alloy, this work shows significant improvement in the cost of preparing the training set. As shown in Fig. 5, the cost for generating a training set with raw features increases when the number of elemental types of alloy increases. By using the expandable features, however, the cost for generating a binary alloy dataset is only required. To schematically evaluate the cost for training set, the average cost for equiatomic 3*d* transition metals is calculated with AKAI-KKR-CPA^40^ code in 2.1 GHz Intel Xeon E5-2620 processor. By considering the number of possible configurations with the cost, the total cost for the binary alloy dataset requires 0.56 years/core. Likewise, the cost becomes 20.46, 1,144.11, and 159,056.46 years for ternary, quaternary, and quinary alloys, respectively. So, it becomes more than three orders of magnitude larger computational cost for HEA than this work. It implies that for training the machine learning algorithm to predict phases of the unknown HEA, obtaining HEA data in vast compositional space can be bypassed.

We believe that this work can be practically applied to find new multi-element alloy by combining with further experiments, likewise the previous works based on machine learning^49,57^. Further experiment should be needed about the issue which can`t be solved in machine learning level for the lack of data. Combining with this work, new multi-element alloy such as the solid solution of HEA can be practically found by dealing with the issue such as segregation^58^ in the further experiment.

## Conclusion

To conclude, we suggest a learning-based algorithm to predict structural phase of multi-element alloy (binary alloy, ternary alloy and HEA) from binary alloy dataset. In this approach, we transformed the raw features {$n_{d}$^(N)^, $\sigma_{d}$^(N)^} to accurate and expandable features {$n_{d}^{ex}$, $\sigma_{d}^{ex}$}. By employing the {$n_{d}^{ex}$, $\sigma_{d}^{ex}$, *δ*} features, it shows 80.56% accuracy for the experimentally reported multi-element alloys, which shows the practicality of the algorithm. These expandable features enable to obtain comparable accuracy without using the multi-element alloys in the training data. Furthermore, it only requires at least three orders of magnitude smaller computational cost for HEA than generating a training set with raw features.

We suggest that this work can be used to find new multi-element alloy such as the solid solution HEA with further experiment. In further work, we expect the approach can be expanded to find unknown solid solution phase HEA by screening multi-phase and intermetallic phase with training result from binary alloy data, which guarantees small computational cost for the training set.

## Methods

### Dataset for training and test

We make a binary alloy dataset for the training and utilize this for inferencing multi-element alloys. The binary alloy dataset is shown in Fig. 2. The generated dataset consists of 1876 kinds of disordered binary alloys, as indicated below:

$$M_{x}^{{\left( 1 \right)}} M_{{1 - x}}^{{\left( 2 \right)}} (M^{{\left( 1 \right)}} = {\text{ Mn}},{\text{ Fe}},{\text{ Co}},{\text{ Ni}};M^{{\left( 2 \right)}}:{\text{transition}}\;{\text{metal}};\;x = {\mkern 1mu} 0.05,{\mkern 1mu} 0.1,{\mkern 1mu} 0.15 \ldots {\mkern 1mu} 0.95)$$

To prevent the overfitting $\sigma_{d} = 0$ region, we restricted the binary alloy training data by locating magnetic center Mn, Fe, Co, Ni in $M^{\left( 1 \right)}$ site. Since *d* electron bandwidth is broad to stabilize Madelung energy, it prefers closed packed structure such as *bcc*, *fcc,* and *hcp*. Therefore, we only consider *bcc*, *fcc,* and *hcp* structures of each composition^34^. Although the alloy can have additional intermetallic phases, the classification of these simple phases is still important. In HEA, the *fcc* phase HEA has high ductility, and the *bcc* phase HEA has high strength ^10,35^, and the phases are dominant in both binary alloy and HEA when the atomic size difference (*δ*) is small (binary alloy: figure S2, HEA^36,37^). It implies that the classification of the simple lattice structures is valid in some compositional spaces. Therefore, in this work, we focused on our interest in these simple phases. Based on the structural phase of a binary alloy, including intermetallic phases (figure S2), we`ll extend to classify solid solution phases and intermetallic phases of multi-element alloy such as HEA in further work.

In addition, since we used the all-*d*-metal binary alloy data as the training set, we restricted the test set of the multi-element alloy as all-*d*-metal alloy such as CoCrFeMnNi, which is still located in vast compositional space and practically applicable^38,39^.

For generating the dataset, DFT calculation in AKAI-KKR-CPA^40^ code was applied. For the calculation, Korringa-Kohn-Rostoker (KKR)^41^ method is implemented with Coherent-Potential-Approximation (CPA). CPA method effectively consider disordered random alloy such as $M_{x}^{\left( 1 \right)} M_{1 - x}^{\left( 2 \right)}$ by considering one lattice site with the average concentration of $M^{\left( 1 \right)}$ and $M^{\left( 2 \right)}$. To find lattice parameter at the ground state, we calculated the total energy of $M_{x}^{\left( 1 \right)} M_{1 - x}^{\left( 2 \right)}$ with various volumes. The electron exchange–correlation potential is considered with the generalized gradient approximation, Perdew–Wang functional, (GGA91)^42^. Spin orbit coupling (SOC) was considered when 5*d* transition metal in the binary alloy. The structural phase calculation from AKAI-KKR-CPA showed consistency with 165 kinds of compositions among the 212 compositions from the experiment. For predicting the structural phase of multi-element alloy in experimental data, we make the multi-element dataset with 611 binary alloys and 106 ternary alloys from NIMS material database^43^, and 259 HEA data^23,25,44^ was used. Since our attention is restricted to the *d* valence element, we excluded the alloy with *p*, *s,* or *f* valence elements in multi-element alloy.

### Main feature of this work

With {$n_{d}^{ex}$, $\sigma_{d}^{ex}$} feature, we additionally choose features which determine stability of HEA as below equations: $S_{c} = - R\mathop \sum \limits_{i = 1}^{N} c_{i} lnc_{i}$

$${\varvec{\varDelta}}H = 4\mathop \sum \limits_{i = 1,i \ne j}^{N}{\varvec{\varDelta}}H_{ij}^{liq} c_{i} c_{j}$$

$${\text{VEC}} = \mathop \sum \limits_{i = 1}^{N} c_{i} {\text{VEC}}_{i}$$

$$\delta = 100\% \sqrt {\mathop \sum \limits_{i = 1}^{N} c_{i} \left( {1 - \frac{{r_{i} }}{{\tilde{r}_{i} }}} \right)^{2} }$$

$$\chi_{d} = \sqrt {\mathop \sum \limits_{i = 1}^{N} c_{i} \left( {\chi_{i} - \tilde{\chi }_{i} } \right)^{2} }$$

Configurational entropy (S_c_), mixing enthalpy (*ΔH*), valence electron concentration (VEC), atomic size difference (*δ*) and electronegativity difference ($\chi$_*d*_) are used to classify the structural phase of HEA to *bcc*, *fcc,* and non-single phases^22^. *N* is the number of elements in the alloy, and $c_{i}$ is molar fraction of element *i.* ${\varvec{\varDelta}}H_{ij}^{liq}$ is the mixing enthalpy of element *i* and *j* of binary liquid alloy from Miedema`s theory^48^. VEC is evaluated by averaging of VEC of element *i*. Among the features of HEA, VEC is excluded in this work for its similarity with $n_{d}^{ex}$. *ΔH*, is also excluded for its required improvement^60^. Then, the feature set become {$n_{d}^{ex}$, $\sigma_{d}^{ex}$, δ, S_c_, $\chi$_*d*_}. To reduce possible configuration of the feature set, we choose two main features among {$n_{d}^{ex}$, $\sigma_{d}^{ex}$, *δ*, S_c_, $\chi$_*d*_}. In Figure S1, we evaluated the accuracy of the test set with various paired features, and {$n_{d}^{ex}$, $\sigma_{d}^{ex}$} shows the best accuracy, 0.8346. Therefore, we use {$n_{d}^{ex}$, $\sigma_{d}^{ex}$} as two main features and add additional features among the remaining feature set, {*δ*, S_c_, $\chi$_*d*_}.

### Algorithm of this work

**Figure 6:**
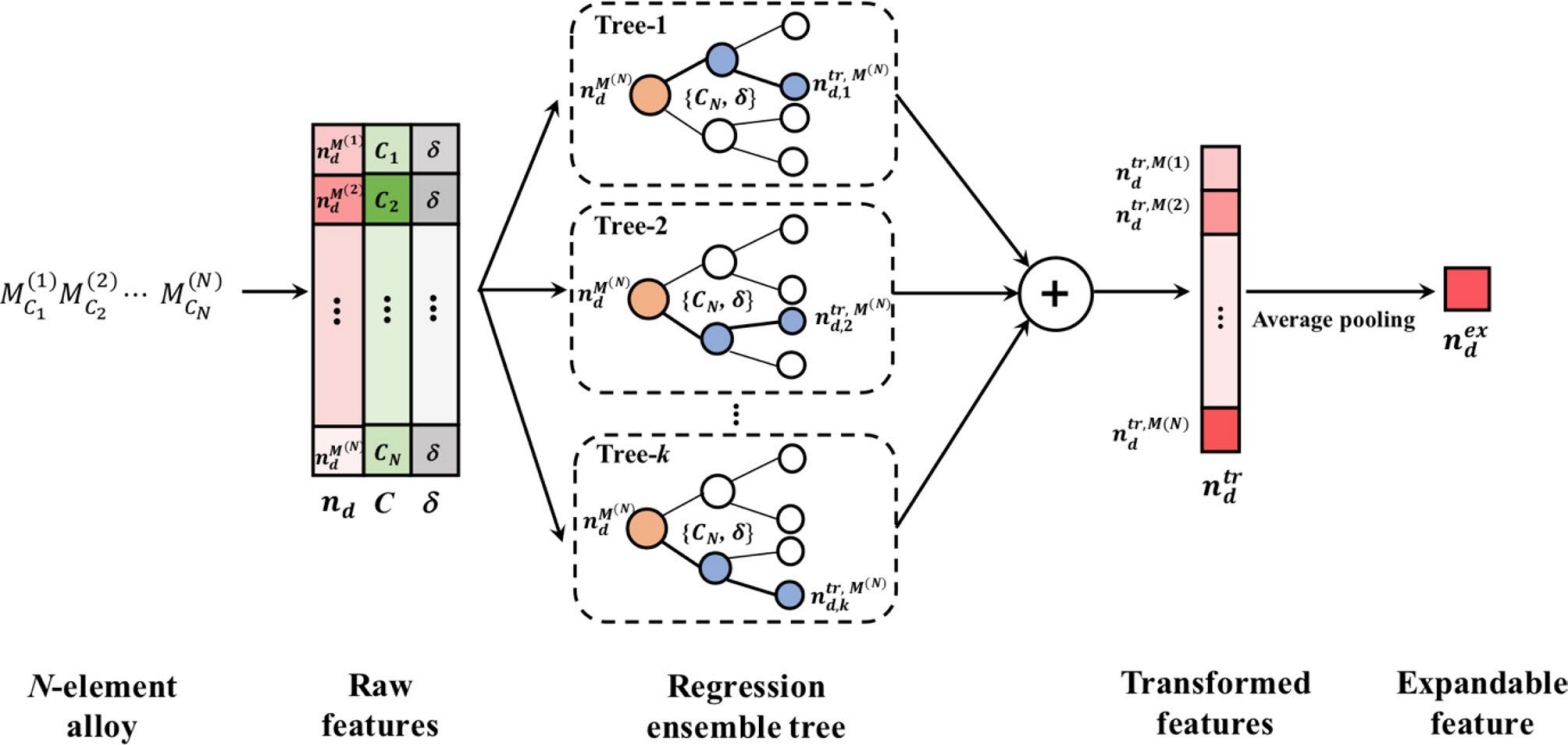
Details of the feature transformation process in this work. Through the ensemble regression tree and average pooling, raw features in the *N*-element alloy are transformed into expandable features. With {$\sigma_{d}^{{M^{N} }}$, $C_{N} ,$ $\delta$} features, $\sigma_{d}^{ex}$ can be obtained in the same way.

Figure 6 describes the detailed process of how raw features in *N*-element alloy can be transformed into expandable features. From the multi-element alloys ($M_{{C_{1} }}^{1} M_{{C_{2} }}^{2} \cdots$ $M_{{C_{N} }}^{N}$), the raw features ({$n_{d}^{{M^{N} }}$, $C_{N} ,$ $\delta$}, {$\sigma_{d}^{{M^{N} }}$, $C_{N} ,$ $\delta$}) can be obtained from their compositional information. To obtain the transformed features ({$n_{d}^{{tr, M^{N} }} , \sigma_{d}^{{tr,M^{N} }}$}), the regression ensemble tree is used. {$C_{N} ,$ $\delta$} features are used as decision rules in the ensemble tree. All parameters such as nodes and depth in the ensemble tree are optimized by training the ensemble tree with the binary alloy data. For training the ensemble tree, {$n_{d}^{{M^{N} }}$} and {$\sigma_{d}^{{M^{N} }}$} of the calculated 1862 binary alloy is used as {$n_{d}^{{tr, M^{N} }}$} and {$\sigma_{d}^{{tr,M^{N} }}$}. From the trained ensemble tree, transformed features in each tree {$n_{d,k}^{{tr, M^{N} }}$} are obtained, and their averaged value is used as the transformed features of the multi-element alloy. Then, by weighted average pooling when {$C_{N}$} used as a weight, 1xN array of the transformed features are reduced to a scalar, expandable features ($n_{d}^{ex} , \sigma_{d}^{ex}$) as follow:

$$n_{d}^{ex} = \frac{{\sum C_{i} n_{d}^{tr,M\left( i \right)} }}{{\sum C_{i} }}$$

For classification of the phase of the multi-element, we utilize the support vector machine (SVM) algorithm with error-correcting output coding (ECOC)^45^ as implemented in MATLAB^46^. Three structural phases, *bcc*, *fcc,* and *hcp*, were used as classes, and all hyper-parameters in the SVM are optimized to minimize the classification error. Various subsets of the feature set {$n_{d}^{ex}$, $\sigma_{d}^{ex}$, δ, S_c_, $\chi$_*d*_} used as input in the algorithm. The *bcc*, *fcc,* and *hcp* phases are represented using integer encoding. To obtain an unbiased prediction error of the classification model, we perform fivefold cross validation^47^. To cope with the nonlinearity using SVM, we used Gaussian kernel function *K* with support vector *x* and kernel scale *G*, $K{ }\sim{ }e^{{ - \left| {x - \overline{x}} \right|/G}}$.

## Supplementary information

Supplementary material 1 (PDF 1213 kb)

## Data Availability

Features and structural phases of experimental data of multi-element alloy (binary alloy, ternary alloy, and HEA) are available in the supplementary section.

## References

[1] Asada T, Terakura K (1993). Generalized-gradient-approximation study of the magnetic and cohesive properties of bcc, fcc, and hcp Mn. Phys. Rev. B.

[2] Rodene DD, Eladgham EH, Gupta RB, Arachchige IU, Tallapally V (2019). Crystal structure and composition-dependent electrocatalytic activity of Ni−Mo nanoalloys for water splitting to produce hydrogen. ACS Appl. Energy Mater..

[3] Walmer MS, Chen CH, Walmer MH (2000). A new class of Sm-TM magnets foroperating temperatures up to 550/spl deg/C. IEEE Trans. Magn..

[4] Ge Q, Neurock M (2004). Structure dependence of NO adsorption and dissociation on platinum surfaces. J. Am. Chem. Soc..

[5] Yao MJ, Pradeep KG, Tasan CC, Raabe D (2014). A novel, single phase, non-equiatomic FeMnNiCoCr high-entropy alloy with exceptional phase stability and tensile ductility. Scrita Mater..

[6] Deng Y, Tasan CC, Pradeep KG, Springer H, Kostka A, Raabe D (2015). Design of a twinning-induced plasticity high entropy alloy. Acta Mater..

[7] Guo S, Ng C, Lu J, Liu CT (2011). Effect of valence electron concentration on stability of fcc or bcc phase in high entropy alloys. J. Appl. Phys..

[8] Chen R, Qin G, Zheng H, Wang L, Su Y, Chiu Y, Ding H, Guo J, Fu H (2018). Composition design of high entropy alloys using the valence electron concentration to balance strength and ductility. Acta Mater..

[9] Guo S (2011). Effect of valence electron concentration on stability of fcc or bcc phase in high entropy alloys. J. Appl. Phys..

[10] Kube SA (2019). Phase selection motifs in High Entropy Alloys revealed through combinatorial methods: Large atomic size difference favors BCC over FCC. Acta Mater..

[11] Wang Y, Lv J, Zhu L, Ma Y (2012). CALYPSO: A method for crystal structure prediction. Comput. Phys. Commun..

[12] Lysgaard S, Mýrdal JSG, Hansen HA, Vegge T (2015). A DFT-based genetic algorithm search for AuCu nanoalloy electrocatalysts for CO2 reduction. Phys Chem. Chem. Phys..

[13] Ryan K, Lengyel J, Shatruk M (2018). Crystal structure prediction via deep learning. J. Am. Chem. Soc..

[14] Prodryabinkin EV, Tikhonov EV, Shapeev AV, Oganov AR (2019). Accelerating crystal structure prediction by machine-learning interatomic potentials with active learning. Phys. Rev. B.

[15] ICSD, *Inorganic Crystal Structure Database; Fachinformationszentrum Karlsruhe*. Karlsruhe, Germany, 2006.

[16] Curtarolo S, Setyawan W, Hart GLW, Jahnatek M, Chepulskii RV, Taylor RH, Wang S, Xue J, Yang K, Levy O, Mehl MJ, Stokes HT, Demchenko DO, Morgan D (2012). AFLOW: An automatic framework for high-throughput materials discovery. Comput. Mater. Sci..

[17] Oliynyk AO, Antono E, Sparks TD, Ghadbeigi L, Gaultois MW, Meredig B, Mar A (2016). High-Throughput machine-learning-driven synthesis of full-heusler compounds. Chem. Mater..

[18] Yeh JW, Chen SK, Lin SJ, Gan JY, Chin TS, Shun TT, Tsau CH, Chang SY (2004). Nanostructured high-entropy alloys with multiple principal elements: novel alloy design concepts and outcomes. Adv. Eng. Mater..

[19] Schmidt J, Shi J, Borlido P, Chen L, Botti S, Marques MAL (2017). Predicting the thermodynamic stability of solids combining density functional theory and machine learning. Chem. Mater..

[20] Faber FA, Lindmaa A, Lilienfeld OAV, Armiento R (2016). Machine learning energies of 2 million Elpasolite (ABC2D6) crystals. Phys. Rev. Lett..

[21] Zhou Z, Zhou Y, He Q, Ding Z, Li F, Yang Y (2019). Machine learning guided appraisal and exploration of phase design for high entropy alloys. NPJ Comput. Mater..

[22] Yao L, Guo W (2019). Machine-learning model for predicting phase formations of high-entropy alloys. Phys. Rev. Mater..

[23] Qi J, Cheung AM, Poon SJ (2019). High entropy alloys mined from binary phase diagrams. Sci. Rep..

[24] Huang W, Martin P, Zhuang HL (2019). Machine-learning phase prediction of high-entropy alloys. Acta Mater..

[25] Ye YF, Wang Q, Lu J, Liu CT, Yang Y (2016). High-entropy alloy: challenges and prospects. Mater. Today.

[26] Miracle DB (2019). High entropy alloys as a bold step forward in alloy development. Nat. Commun..

[27] Oliynyk AO, Adutwum LA, Harynuk JJ, Mar A (2016). Classifying crystal structures of binary compounds ab through cluster resolution feature selection and support vector machine analysis. Chem. Mater..

[28] Skriver HL (1985). Crystal structure from one-electron theory. Phys. Rev. B.

[29] Soderlind P (1994). Crystal structure and elastic-constant anomalies in the magnetic 3*d* transition metals. Phys. Rev. B.

[30] Jin T, Ji HS, Lee YJ, Kim JY, Kwon SK, Lee C, Shim JH (2018). Descriptor-based crystal structure prediction of magnetic transition metals: Orbital-spin occupancy rule. AIP Adv..

[31] Opitz D, Maclin R (1999). Popular ensemble methods: an empirical study. J. Artif. Intell. Res..

[32] Velisavljevic N, Chesnut GN (2007). Direct hcp → bcc structural phase transition observed in titanium alloy at high pressure. Appl. Phys. Lett..

[33] Han JW, Oda T (2017). Chemical states of 3d transition metal impurities in a liquid lead–bismuth eutectic analyzed using first principles calculations. Phys. Chem. Chem. Phys..

[34] Söderlind P, Eriksson O, Johansson B, Wills JM, Boring AM (1995). A unified picture of the crystal structures of metals. Nature.

[35] Kube SA, Sohn S, Uhl D, Datye A, Mehta A, Schroers J (2019). Phase selection motifs in High Entropy Alloys revealed through combinatorial methods: Large atomic size difference favors BCC over FCC. Acta. Mater..

[36] Yang X, Zhang Y (2012). Prediction of high-entropy stabilized solid-solution in multi-component alloys. Mater. Chem. Phys.

[37] Guo S (2013). More than entropy in high-entropy alloys: Forming solid solutions or amorphous phase. Intermetallics.

[38] Yao MJ (2014). A novel, single phase, non-equiatomic FeMnNiCoCr high-entropy alloy with exceptional phase stability and tensile ductility. Scripta Mater..

[39] Bludovatz B (2014). A fracture-resistant high-entropy alloy for cryogenic applications. Science.

[40] Akai-kkr: http://sham.phys.sci.osaka-u.ac.jp/˜kkr/

[41] Gyorffy BL (1972). Coherent-potential approximation for a nonoverlapping-muffin-tin-potential model of random substitutional alloys. Phys. Rev. B.

[42] Mlynarski P, Salahub DR (1991). Self-consistent implementation of nonlocal exchange and correlation in a Gaussian density-functional method *Phys*. Rev. B.

[43] NIMS database: http://crystdb.nims.go.jp/crystdb/search-materials

[44] Senkov ON, Miller JD, Miracle DB, Woodward C (2015). Accelerated exploration of multi-principal element alloys for structural applications. Coupling Phase Diagrams Thermochem..

[45] Escalera S, Pujol O, Radeva P (2010). On the decoding process in ternary error-correcting output codes. IEEE Trans. Pattern Anal. Mach. Intell..

[46] MATLAB and Statistics and Machine Learning Toolbox Release 2018 The MathWorks Inc Natick Massachusetts, United States

[47] R. Kohavi, A study of cross-validation and bootstrap for accuracy estimation and model selection. *Proceedings of the Fourteenth International Joint Conference on Artificial Intelligence. San Mateo, CA: Morgan Kaufmann,* **2** (1995), p. 1137.

[48] Miedema AR, Châtel PFD, Boer FRD (1980). Cohesion in alloys—fundamentals of a semi-empirical model. Physica B+C.

[49] Wen C, Zhang Y, Wang C, Xue D, Bai Y, Antonov S, Dai L, Lookman T, Su Y (2019). Machine learning assisted design of high entropy alloys with desired property. Acta Mater..

[50] Cortes C, Vapnik V (1995). Support-vector networks. Mach. Learn..

[51] Senkov ON, Wilks GB, Scott JM, Miracle DB (2011). Mechanical properties of Nb25Mo25Ta25W25 and V20Nb20Mo20Ta20W20 refractory high entropy alloys. Intermetallics.

[52] Otto F, Dlouhý A, Somsen Ch, Bei H, Eggeler G, George EP (2011). The influences of temperature and microstructure on the tensile properties of a CoCrFeMnNi high-entropy alloy. Acta Mater..

[53] Yeh JW (2013). Alloy design strategies and future trends in high-entropy alloys. JOM.

[54] G. Martius, and C. H Lampert, Extrapolation and learning equations. **2016**. arXiv preprint https://arxiv.org/abs/1610.02995.

[55] Pei Z, Yin J, Hawk JA, Alman DE, Gao MC (2020). Machine-learning informed prediction of high-entropy solid solution formation: Beyond the Hume-Rothery rules. Npj Comput. Mater..

[56] Zhang L, Chen H, Tao X, Cai H, Liu J, Ouyang Y, Peng Q, Du Y (2020). Machine learning reveals the importance of the formation enthalpy and atom-size difference in forming phases of high entropy alloys. Mater. Des..

[57] Kaufmann K (2020). Discovery of high-entropy ceramics via machine learning. NPJ Comput. Mater..

[58] Todai M (2017). Novel TiNbTaZrMo high-entropy alloys for metallic biomaterials. Scripta Mater..

[59] Maaten L, Hinton G (2008). Visualing data using t-SNE. J. Mach. Learn. Res..

[60] Zhang RF, Rajan K (2014). Statistically based assessment of formation enthalpy for intermetallic compounds. Chem. Phys. Lett..

