# Supplementary material for: Accelerated crystal structure prediction of multi-elements random alloy using expandable features
Source: Sci Rep. 2021 Mar 4;11:5194. doi: 10.1038/s41598-021-84544-8 (PMC7933338; doi:10.1038/s41598-021-84544-8)
Supplement: Supplementary file 1 — Supplementary material 1 (PDF 1213 kb) [file 41598_2021_84544_MOESM1_ESM.pdf]

Supplementary materials for

Accelerated crystal structure prediction of multi-elements random alloy using expandable features

Taewon Jin[1,†], Ina Park[1], Taesu Park[1], Jaesik Park[2,3*], and Ji Hoon Shim[1,3,4*]

[1]*Department of Chemistry, Pohang University of Science and Technology, Pohang 37673, Republic of Korea*

[2]*Department of Computer Science and Engineering, Pohang University of Science and Technology, Pohang 37673, Republic of Korea*

[3]*Graduate School of Artificial Intelligence, Pohang University of Science and Technology, Pohang 37673, Republic of Korea*

[4]*Department of Physics and Division of Advanced Materials Science, Pohang University of Science and Technology, Pohang 37673, Republic of Korea*

†Current address: Department of Chemical and Biomolecular Engineering, Korea Advanced Institute of Science and Technology (KAIST), 291 Daehak-ro, Yuseong-gu, Daejeon, 34141, Republic of Korea

Contents

Figure S1. Test set error of paired features among $\{n_d^{ex},\ \sigma_d^{ex}, \delta, S_c,\ \chi_d\}$.

Figure S2. Data distribution of solid solution phase and intermetallic phase of experimental binary alloy.

Figure S3. Confusion matrices for training data (calculated binary alloy) and test data (experimental binary alloy, ternary alloy and HEA).

Table S1. Structural and feature values of single phase binary alloy from experiment.

Table S2. Structural and feature values of single phase ternary alloy and HEAs from experiment.

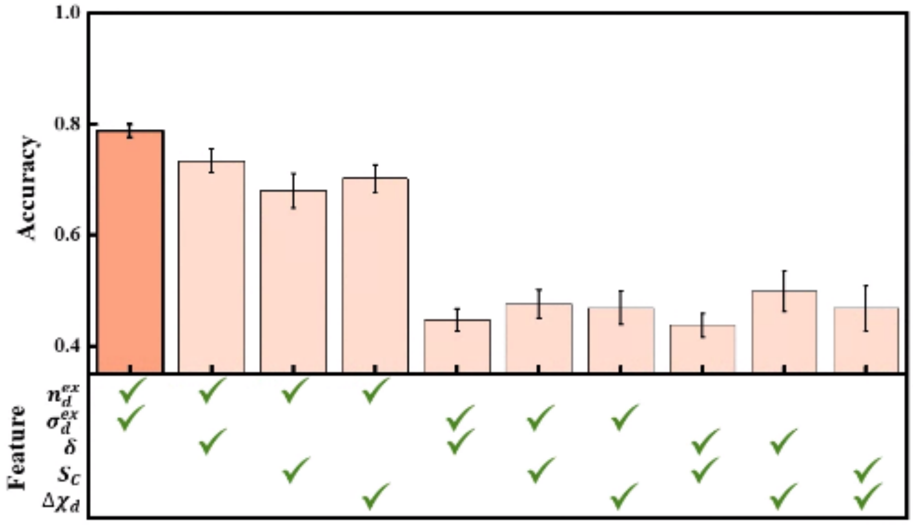

Figure S1. Test set error of paired features among $\{n_d^{ex},\ \sigma_d^{ex}, \delta, S_c,\ \chi_d\}$.

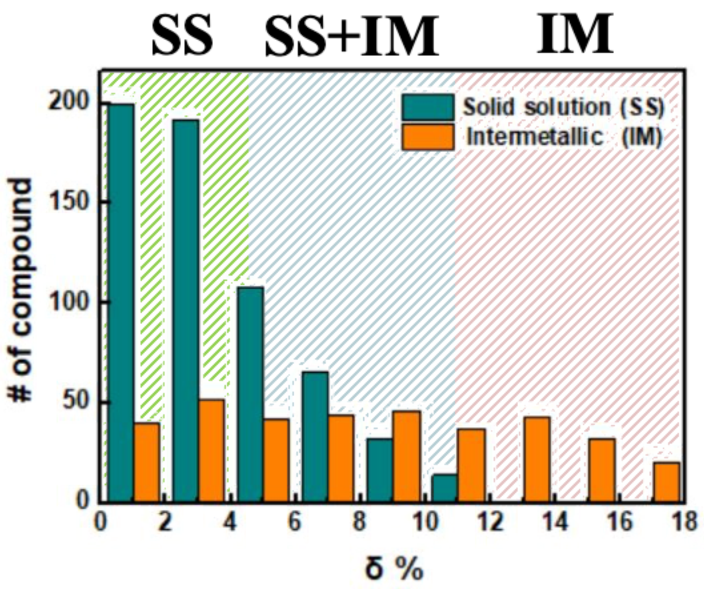

Figure S2. Data distribution of solid solution phase and intermetallic phase of experimental binary alloy. According to atomic size difference(δ), the binary alloy data has similar trend with HEA[5], so we expect that solid solution phase and intermetallic phase of multi-element alloy such as HEA can be predicted with the machine learning algorithm trained by binary alloy data.

Predicted

Training data (Cal.)

Target	*bcc*	*fcc*	*hcp*
bcc	**89.26 %**	3.05 %	7.69 %
fcc	6.53 %	**81.83 %**	11.63 %
hcp	11.43 %	15.97 %	**72.60 %**

Test data (Exp.)

Target	*bcc*	*fcc*	*hcp*
bcc	**94.63 %**	1.13 %	4.24 %
fcc	11.71 %	**70.47 %**	17.82 %
hcp	24.30 %	9.58 %	**66.13 %**

Figure S3. Confusion matrices for training data (calculated binary alloy) and test data (experimental binary alloy, ternary alloy and HEA).

Table S1. Structural and feature values of single phase binary alloy from experiment. The expandable features $\{n_d^{ex},\ \sigma_d^{ex}\}$ are obtained using the trained feature transformation module. Atomic size difference (δ), configuration entropy (S_c), electronegativity difference (χ_d) are obtained using equation in method section. Sample preparation methods are also denoted in the table.

Composition	n_d^{ex}	σ_d^{ex}	δ	S_c	χ_d	Phase	Preparation method	Ref
Mn0.1Ti0.9	2.6601	0.3023	2.5788	2.7027	0.0030	BCC		49
Mn0.5V0.5	4.3470	0.4685	3.0120	5.7628	0.0400	BCC	Arc-melting	275
Mn0.565V0.435	4.4507	0.4264	2.9982	5.6924	0.0397	BCC	Arc-melting	68
Mn0.9V0.1	4.9854	1.2090	1.8519	2.7027	0.0240	BCC	Arc-melting	68
Mn0.253Cr0.747	4.6228	0.2515	1.3195	4.7025	0.0478	BCC	Arc-melting	310
Mn0.43Cr0.57	4.7466	0.4113	1.5108	5.6811	0.0545	BCC	Melting	288
Mn0.5Cr0.5	4.7955	0.5706	1.5291	5.7628	0.0550	BCC	Arc-melting	255
Mn0.6Cr0.4	4.8654	0.4033	1.5028	5.5954	0.0539	BCC	Arc-melting	255
Mn0.67Cr0.33	4.9143	0.4214	1.4455	5.2726	0.0517	BCC	Melting	199
Mn0.99Cr0.01	5.1380	0.6413	0.3089	0.4656	0.0109	BCC	Melting	199
Mn0.05Fe0.95	6.2774	2.5413	0.6974	1.6505	0.0610	BCC		75
Mn0.18Fe0.82	6.1224	2.5595	1.2243	3.9192	0.1076	BCC	Vapor quenching	170
Mn0.298Fe0.702	5.9818	1.7659	1.4521	5.0646	0.1281	FCC	As-cast, water quenched	309
Mn0.3Fe0.7	5.9794	1.7813	1.4548	5.0787	0.1283	HCP	Arc-melting	136
Mn0.35Fe0.65	5.9198	1.7883	1.5118	5.3829	0.1336	FCC	Melting	191
Mn0.5Fe0.5	5.7410	1.9479	1.5773	5.7628	0.1400	FCC		111
Mn0.65Fe0.35	5.5622	0.8741	1.4975	5.3829	0.1336	BCC	Melting	191
Mn0.5Co0.5	6.2275	1.8005	2.8754	5.7628	0.1650	FCC	As-cast	160
Mn0.139Ni0.861	7.8950	1.1185	2.7553	3.3517	0.1245	FCC	Melting, as-cast	201
Mn0.22Ni0.78	7.6363	1.3206	3.2781	4.3807	0.1491	FCC	Arc-melting	313
Mn0.25Ni0.75	7.5405	1.4015	3.4185	4.6753	0.1559	FCC		258
Mn0.5Ni0.5	6.7420	1.9584	3.8710	5.7628	0.1800	FCC	Arc-melting	313
Mn0.7Ni0.3	6.1032	1.7453	3.4937	5.0787	0.1650	FCC	Melting, as-cast	201
Mn0.8Ni0.2	5.7838	0.8441	3.0265	4.1603	0.1440	FCC	melting	141
Mn0.874Ni0.126	5.5474	0.6213	2.4969	3.1486	0.1195	FCC	Melting, as-cast	201
Mn0.5Cu0.5	7.1660	1.6585	5.2288	5.7628	0.1750	FCC	melting	138
Mn0.15Mo0.85	4.4285	0.5052	5.5777	3.5144	0.2178	BCC	Arc-melting	76
Mn0.5Ru0.5	5.8655	1.6948	5.0147	5.7628	0.3250	HCP	Arc-melting	87
Mn0.3Rh0.7	6.6885	1.0460	3.2462	5.0787	0.3345	FCC	Arc-melting	87

Mn0.23Pd0.77	7.6952	0.7587	2.0140	4.4835	0.2735	FCC	Arc-melting	128
Mn0.25Pd0.75	7.6290	0.8346	2.0743	4.6753	0.2815	FCC	Induction-melting	33
Mn0.3Pd0.7	7.4634	1.0185	2.2005	5.0787	0.2979	FCC	Induction heating	162
Mn0.31Pd0.69	7.4303	1.0521	2.2219	5.1472	0.3006	FCC	Induction heating	162
Mn0.16Ag0.84	8.5134	0.3424	0.8922	3.6554	0.1393	FCC		188
Mn0.97Ag0.03	5.2653	0.6284	0.4235	1.1202	0.0648	FCC		87
Mn0.47Re0.53	4.9712	1.5081	7.6868	5.7479	0.1747	HCP	Arc-melting	263
Mn0.4Ir0.6	6.2250	1.4006	5.3991	5.5954	0.3184	FCC	Arc-melting	87
Mn0.8Ir0.2	5.5050	1.7307	4.6117	4.1603	0.2600	FCC	Arc-melting	268
Mn0.2Pt0.8	7.4498	0.6681	3.6824	4.1603	0.2920	FCC	Arc-melting	87
Mn0.25Pt0.75	7.3058	0.7492	4.0047	4.6753	0.3161	FCC	Arc-melting	45
Mn0.33Pt0.67	7.0753	0.4774	4.3812	5.2726	0.3433	FCC	Arc-melting	163
Mn0.5Pt0.5	6.5855	1.7936	4.7337	5.7628	0.3650	FCC	Arc-melting	149
Mn0.19Au0.81	8.0675	0.4897	2.9732	4.0425	0.3884	FCC	Melting	246
Mn0.24Au0.76	7.8871	0.8325	3.2491	4.5817	0.4228	HCP		54
Fe0.25Ti0.75	3.3723	0.7130	5.0645	4.6753	0.1256	BCC	Vapor quenching	167
Fe0.35Ti0.65	3.7676	0.9393	5.6446	5.3829	0.1383	BCC		247
Fe0.8Ti0.2	5.5464	2.1978	5.0000	4.1603	0.1160	BCC	Vapor quenching	167
Fe0.9Ti0.1	5.9417	2.4086	3.7975	2.7027	0.0870	BCC	Vapor quenching	167
Fe0.91Ti0.09	5.9812	2.4313	3.6271	2.5153	0.0830	BCC		193
Fe0.965Ti0.035	6.1986	2.4838	2.3456	1.2614	0.0533	BCC		75
Fe0.975Ti0.025	6.2382	2.4079	1.9952	0.9720	0.0453	BCC	Melting, cast	56
Fe0.98Ti0.02	6.2579	2.4533	1.7903	0.8151	0.0406	BCC	Arc-melting	11
Fe0.4V0.6	4.6642	0.9113	4.4536	5.5954	0.0980	BCC	Arc-melting	280
Fe0.5V0.5	4.9430	1.2290	4.5872	5.7628	0.1000	BCC	Arc-melting	280
Fe0.55V0.45	5.0824	1.3502	4.5852	5.7212	0.0995	BCC	Arc-melting	12
Fe0.65V0.35	5.3612	1.3978	4.4369	5.3829	0.0954	BCC	Arc-melting	301
Fe0.7V0.3	5.5006	1.6643	4.2828	5.0787	0.0917	BCC		134
Fe0.75V0.25	5.6400	1.8923	4.0658	4.6753	0.0866	BCC		225
Fe0.8V0.2	5.7794	2.1360	3.7736	4.1603	0.0800	BCC	Arc-melting	163
Fe0.96V0.04	6.2255	2.3383	1.8770	1.3963	0.0392	BCC	Arc-melting	11
Fe0.99V0.01	6.3091	2.5372	0.9558	0.4656	0.0199	BCC	Arc-melting	11
Fe0.0195Cr0.9805	4.4829	0.0443	0.8340	0.7989	0.0235	BCC	Induction-melting	115
Fe0.026Cr0.974	4.4952	0.0550	0.9601	1.0023	0.0271	BCC	Induction-melting	115
Fe0.038Cr0.962	4.5179	0.0718	1.1544	1.3430	0.0325	BCC	Induction-melting	115
Fe0.0665Cr0.9335	4.5718	0.1311	1.5070	2.0327	0.0424	BCC	Induction-melting	115
Fe0.2Cr0.8	4.8242	0.4079	2.4390	4.1603	0.0680	BCC		304
Fe0.387Cr0.613	5.1778	0.3473	3.0042	5.5487	0.0828	BCC	Induction-melting	115

Fe0.5Cr0.5	5.3915	0.7560	3.1056	5.7628	0.0850	BCC		271
Fe0.8Cr0.2	5.9588	1.8265	2.5316	4.1603	0.0680	BCC		7
Fe0.92Cr0.08	6.1857	2.3116	1.7302	2.3177	0.0461	BCC	Levitation-melting	184
Fe0.95Cr0.05	6.2425	2.3392	1.3926	1.6505	0.0371	BCC	Arc-melting	259
Fe0.96Cr0.04	6.2614	2.4553	1.2529	1.3963	0.0333	BCC		15
Fe0.97Cr0.03	6.2803	2.4753	1.0914	1.1202	0.0290	BCC		75
Fe0.8Mn0.2	6.0986	2.2876	1.2739	4.1603	0.1120	BCC	Vapor quenching	170
Fe0.87Mn0.13	6.1820	2.4602	1.0734	3.2124	0.0942	BCC		111
Fe0.95Mn0.05	6.2774	2.5413	0.6974	1.6505	0.0610	BCC		75
Fe0.96Mn0.04	6.2893	2.5140	0.6273	1.3963	0.0549	BCC		15
Fe0.97Mn0.03	6.3012	2.5167	0.5462	1.1202	0.0478	BCC	Arc-melting	11
Fe0.05Co0.95	7.2614	1.7486	0.5728	1.6505	0.0109	HCP		274
Fe0.1Co0.9	7.2127	1.7768	0.7874	2.7027	0.0150	FCC		291
Fe0.25Co0.75	7.0668	2.0134	1.1321	4.6753	0.0217	BCC	Arc-melting	163
Fe0.28Co0.72	7.0376	2.1106	1.1729	4.9298	0.0224	BCC	KOH solution hetead	117
Fe0.5Co0.5	6.8235	2.1424	1.2987	5.7628	0.0250	BCC		122
Fe0.6Co0.4	6.7262	2.1909	1.2692	5.5954	0.0245	BCC	Arc-melting	55
Fe0.88Co0.12	6.4538	2.4761	0.8358	3.0506	0.0162	BCC	Induction-melting	139
Fe0.91Co0.09	6.4246	2.5054	0.7355	2.5153	0.0143	BCC	Arc-melting	11
Fe0.94Co0.06	6.3954	2.4735	0.6099	1.8870	0.0119	BCC	Levitation-melting	184
Fe0.95Co0.05	6.3857	2.4821	0.5596	1.6505	0.0109	BCC	Arc-melting	11
Fe0.5Ni0.5	7.3380	1.7685	2.2951	5.7628	0.0400	FCC	Evaporation method	143
Fe0.6Ni0.4	7.1378	1.8887	2.2384	5.5954	0.0392	BCC	Evaporation method	143
Fe0.9Ni0.1	6.5372	2.2987	1.3522	2.7027	0.0240	BCC		276
Fe0.95Ni0.05	6.4371	2.4607	0.9802	1.6505	0.0174	HCP		276
Fe0.49Cu0.51	7.7905	1.3076	3.6564	5.7612	0.0350	BCC	Vapor phase transport	62
Fe0.6Cu0.4	7.4770	1.5324	3.5547	5.5954	0.0343	BCC	Vapor quenching	171
Fe0.7Cu0.3	7.1920	1.8266	3.3011	5.0787	0.0321	BCC	Milling	44
Fe0.8Cu0.2	6.9070	1.8203	2.8609	4.1603	0.0280	BCC		295
Fe0.2Nb0.8	3.2762	0.5981	8.8608	4.1603	0.0920	FCC	E-gun evaporating	39
Fe0.2Mo0.8	4.7090	0.5986	7.4236	4.1603	0.1320	BCC	Rf sputtering	168
Fe0.27Mo0.73	4.8515	0.8156	8.3479	4.8492	0.1465	FCC	E-gun evaporating	324
Fe0.8Mo0.2	5.9300	2.1529	8.3538	4.1603	0.1320	BCC	Rf sputtering	168
Fe0.86Mo0.14	6.0521	2.2919	7.3386	3.3669	0.1145	BCC		193
Fe0.875Mo0.125	6.0826	2.2864	7.0168	3.1325	0.1091	BCC		6
Fe0.92Mo0.08	6.1742	2.3902	5.8115	2.3177	0.0895	BCC	Arc-melting	265
Fe0.96Mo0.04	6.2556	2.5606	4.2340	1.3963	0.0647	BCC	Arc-melting	265
Fe0.97Mo0.03	6.2760	2.5833	3.6938	1.1202	0.0563	BCC	Arc-melting	265

Fe0.5Ru0.5	6.4615	1.5208	6.5868	5.7628	0.1850	HCP	Arc-melting	108
Fe0.9Ru0.1	6.3619	2.4129	4.1719	2.7027	0.1110	BCC	Levitation-melting	184
Fe0.3Rh0.7	7.0461	0.8344	4.6399	5.0787	0.2062	FCC		166
Fe0.5Rh0.5	6.8435	1.4975	5.1672	5.7628	0.2250	FCC	Induction-melting	34
Fe0.7Rh0.3	6.6409	1.9861	4.8357	5.0787	0.2062	BCC	Vapor quenching	315
Fe0.92Rh0.08	6.4180	2.1182	2.9308	2.3177	0.1221	BCC	Levitation-melting	184
Fe0.94Rh0.06	6.3978	2.4050	2.5712	1.8870	0.1069	BCC	Levitation-melting	184
Fe0.5Pd0.5	7.3970	1.4007	4.0000	5.7628	0.1850	FCC		318
Fe0.6Pd0.4	7.1850	1.6206	3.9508	5.5954	0.1813	BCC	Vapor quenching	254
Fe0.8Pd0.2	6.7610	2.1048	3.2787	4.1603	0.1480	BCC	Vapor quenching	254
Fe0.97Pd0.03	6.4006	2.3844	1.4180	1.1202	0.0631	BCC		75
Fe0.5Ag0.5	7.7460	1.2946	2.8037	5.7628	0.0500	FCC		169
Fe0.78Ag0.22	6.9570	2.0202	2.3599	4.3807	0.0414	BCC		169
Fe0.975Ta0.025	6.2619	2.5741	4.3727	0.9720	0.0515	BCC		229
Fe0.3W0.7	4.7984	0.8868	9.3213	5.0787	0.2429	BCC	Vapor quenching	195
Fe0.6W0.4	5.4578	1.6856	10.6125	5.5954	0.2596	BCC	Vapor quenching	195
Fe0.8W0.2	5.8974	2.1445	9.0575	4.1603	0.2120	BCC	Vapor quenching	195
Fe0.93W0.07	6.1831	2.3720	5.9527	2.1088	0.1352	BCC	melting	42
Fe0.94W0.06	6.2051	2.4090	5.5537	1.8870	0.1259	BCC		193
Fe0.96W0.04	6.2491	2.4629	4.6041	1.3963	0.1039	BCC	Arc-melting	265
Fe0.98W0.02	6.2930	2.6241	3.3048	0.8151	0.0742	BCC		75
Fe0.92Re0.08	6.2154	2.4183	5.4751	2.3177	0.0190	BCC	Levitation-melting	184
Fe0.7Os0.3	6.2341	1.9358	8.0689	5.0787	0.1696	HCP	Arc-melting	136
Fe0.985Os0.015	6.3319	2.4286	2.2533	0.6475	0.0450	BCC		75
Fe0.5Ir0.5	6.6410	1.5229	7.1429	5.7628	0.1850	FCC		166
Fe0.64Ir0.36	6.5559	1.9475	6.9971	5.4325	0.1776	HCP		97
Fe0.9Ir0.1	6.3978	2.3517	4.5455	2.7027	0.1110	BCC	Levitation-melting	184
Fe0.5Pt0.5	7.1815	1.5310	6.3063	5.7628	0.2250	FCC		173
Fe0.75Pt0.25	6.7593	2.0448	5.6392	4.6753	0.1949	BCC	Arc-melting	16
Fe0.85Pt0.15	6.5904	2.2951	4.7116	3.5144	0.1607	BCC	Arc-melting	163
Fe0.9Pt0.1	6.5059	2.4438	3.9848	2.7027	0.1350	BCC	Levitation-melting	184
Fe0.97Pt0.03	6.3877	2.4428	2.2871	1.1202	0.0768	BCC		75
Fe0.08Au0.92	8.5597	0.2097	2.8299	2.3177	0.1926	FCC		85
Fe0.5Au0.5	7.5450	1.4540	5.4545	5.7628	0.3550	FCC		85
Fe0.96Au0.04	6.4336	2.3700	2.2507	1.3963	0.1391	BCC		64
Co0.87Ti0.13	6.6696	1.4659	5.2033	3.2124	0.1143	FCC		284
Co0.7V0.3	6.1817	1.0629	5.5212	5.0787	0.1146	FCC	Arc-melting	305
Co0.75V0.25	6.3698	1.2776	5.2486	4.6753	0.1083	FCC	Melting	306

Co0.876V0.124	6.8436	1.5146	4.0569	3.1163	0.0824	FCC	Arc-melting	307
Co0.75Cr0.25	6.5940	1.2574	3.8985	4.6753	0.0953	HCP	Arc-melting	163
Co0.9Cr0.1	7.0236	1.6306	2.7379	2.7027	0.0660	FCC	Arc-melting	163
Co0.5Ni0.5	7.8245	1.1983	0.9967	5.7628	0.0150	FCC		23
Co0.75Ni0.25	7.5673	1.4386	0.8589	4.6753	0.0130	HCP		23
Co0.23Nb0.77	3.6148	0.4557	10.3288	4.4835	0.1178	FCC	Sputtering	322
Co0.86Nb0.14	6.6381	1.3814	10.0741	3.3669	0.0972	BCC		203
Co0.935Nb0.065	6.9981	1.4738	7.3167	1.9996	0.0690	FCC		203
Co0.08Mo0.92	4.5426	0.1326	5.5141	2.3177	0.0760	BCC	Powder metallurgical technique	272
Co0.6Mo0.4	6.1068	1.0417	11.1340	5.5954	0.1372	HCP	Arc-melting	163
Co0.9Mo0.1	7.0092	1.4170	7.3171	2.7027	0.0840	HCP	Arc-melting	163
Co0.5Tc0.5	6.3780	0.8351	9.2537	5.7628	0.0100	HCP	Arc-melting	148
Co0.5Ru0.5	6.9480	1.0013	7.8788	5.7628	0.1600	HCP		298
Co0.6Ru0.4	7.0204	1.1870	7.8432	5.5954	0.1568	FCC		298
Co0.5Rh0.5	7.3300	1.0152	6.4615	5.7628	0.2000	FCC		298
Co0.75Rh0.25	7.3200	1.3019	5.7827	4.6753	0.1732	HCP		298
Co0.5Pd0.5	7.8835	0.9012	5.2960	5.7628	0.1600	FCC	Melting in plasma	312
Co0.8W0.2	6.6758	1.3056	10.2372	4.1603	0.1920	FCC	Arc-melting	163
Co0.88W0.12	6.9295	1.4652	8.4906	3.0506	0.1560	FCC		182
Co0.5Re0.5	6.0635	0.9171	10.5882	5.7628	0.0100	HCP		298
Co0.6Re0.4	6.3128	1.0487	10.5988	5.5954	0.0098	FCC		298
Co0.5Os0.5	6.6520	0.9217	9.7923	5.7628	0.1600	HCP		298
Co0.6Os0.4	6.7836	1.0730	9.7861	5.5954	0.1568	FCC		298
Co0.8Os0.2	7.0468	1.3486	8.3228	4.1603	0.1280	FCC		298
Co0.5Ir0.5	7.1275	0.9896	8.4337	5.7628	0.1600	FCC		298
Co0.65Ir0.35	7.1823	1.2868	8.2541	5.3829	0.1526	HCP	Arc-melting	163
Co0.5Pt0.5	7.6680	1.0160	7.5988	5.7628	0.2000	FCC		63
Co0.6Pt0.4	7.5964	1.1934	7.5602	5.5954	0.1960	HCP	Arc-melting	163
Co0.02Au0.98	8.7241	0.0207	1.7746	0.8151	0.0924	FCC	Induction-melting, water-quenching	302
Co0.975Au0.025	7.3461	1.6627	2.2516	0.9720	0.1030	FCC		85
Ni0.19V0.81	4.4591	0.1179	5.1736	4.0425	0.1098	BCC		77
Ni0.47V0.53	5.8003	0.0996	6.8344	5.7479	0.1397	BCC		223
Ni0.6V0.4	6.4230	0.1413	6.8300	5.5954	0.1372	FCC		221
Ni0.67V0.33	6.7583	0.2328	6.6202	5.2726	0.1317	FCC		250
Ni0.75V0.25	7.1415	0.4623	6.1659	4.6753	0.1212	FCC	Arc-melting	313
Ni0.92V0.08	7.9558	0.5893	3.9589	2.3177	0.0760	FCC	Arc-melting	163

Ni0.2Cr0.8	5.2246	0.0255	4.1820	4.1603	0.1000	BCC		22
Ni0.98Zr0.02	8.2047	0.4973	5.3150	0.8151	0.0812	FCC		258
Ni0.99Zr0.01	8.2719	0.6874	3.7918	0.4656	0.0577	FCC	Arc-melting	313
Ni0.2Nb0.8	3.6766	0.0091	10.4145	4.1603	0.1240	FCC	Sputtering	323
Ni0.85Nb0.15	7.4648	0.1078	11.1906	3.5144	0.1107	FCC	Splat-cooling	223
Ni0.9Nb0.1	7.7562	0.2944	9.5517	2.7027	0.0930	FCC		258
Ni0.92Nb0.08	7.8728	0.4778	8.6930	2.3177	0.0841	FCC	Arc-melting	163
Ni0.94Nb0.06	7.9893	0.5135	7.6588	1.8870	0.0736	FCC	Arc-melting	313
Ni0.02Mo0.98	4.3827	0.0087	3.0341	0.8151	0.0350	BCC	Cold pressed into small bars	225
Ni0.16Mo0.84	4.9479	0.0486	8.1939	3.6554	0.0917	FCC	E-gun evaporation system	324
Ni0.73Mo0.27	7.2490	0.0088	11.3715	4.8492	0.1110	FCC		258
Ni0.75Mo0.25	7.3298	0.0112	11.1482	4.6753	0.1083	FCC		250
Ni0.76Mo0.24	7.3701	0.2381	11.0239	4.5817	0.1068	FCC	Cold pressed into small bars	225
Ni0.91Mo0.09	7.9757	0.4976	7.6845	2.5153	0.0715	FCC	Arc-melting	313
Ni0.83Tc0.17	7.8472	0.3751	8.2514	3.7902	0.0038	FCC		281
Ni0.5Rh0.5	7.8445	0.3664	7.4534	5.7628	0.1850	FCC	Heating	109
Ni0.526Pd0.474	8.3949	0.2831	6.3014	5.7516	0.1448	FCC	Induction-melting	183
Ni0.8Pd0.2	8.3626	0.2783	5.2288	4.1603	0.1160	FCC		258
Ni0.81Pd0.19	8.3614	0.2899	5.1348	4.0425	0.1138	FCC	Arc-melting	313
Ni0.92Pd0.08	8.3484	0.6447	3.6028	2.3177	0.0787	FCC	Arc-melting	163
Ni0.5Ag0.5	8.7470	0.1698	5.0955	5.7628	0.0100	FCC	Arc-melting	245
Ni0.99Hf0.01	8.2755	0.6500	3.9243	0.4656	0.0607	FCC	Arc-melting	313
Ni0.85Ta0.15	7.5878	0.1030	11.6251	3.5144	0.1464	FCC	Splat-cooling	223
Ni0.9Ta0.1	7.8382	0.3089	9.9286	2.7027	0.1230	FCC		127
Ni0.92Ta0.08	7.9384	0.4580	9.0384	2.3177	0.1112	FCC	Arc-melting	313
Ni0.9576Ta0.0424	8.1267	0.4605	6.7983	1.4591	0.0826	FCC	Levitation-melting	123
Ni0.85W0.15	7.7090	0.1803	10.0971	3.5144	0.1607	FCC		106
Ni0.91W0.09	7.9610	0.4138	8.2322	2.5153	0.1288	FCC	Arc-melting	313
Ni0.92W0.08	8.0030	0.4903	7.8264	2.3177	0.1221	FCC	Arc-melting	163
Ni0.5Ir0.5	7.6420	0.4122	9.4225	5.7628	0.1450	FCC		80
Ni0.5Pt0.5	8.1825	0.4207	8.5890	5.7628	0.1850	FCC		279
Ni0.75Pt0.25	8.2608	0.4122	7.7720	4.6753	0.1602	FCC	Arc-melting	313
Ni0.92Pt0.08	8.3140	0.5425	5.0226	2.3177	0.1004	FCC		279
Au0.11Mo0.89	4.5120	0.0000	2.6595	2.8809	0.1189	BCC		113
Nb0.75Au0.25	4.5288	0.0000	5.4127	4.6753	0.4070	BCC	Arc-melting	175

Nb0.7Au0.3	4.8223	0.0000	5.7642	5.0787	0.4308	BCC	Arc-melting	198
V0.7Au0.3	4.8989	0.0000	0.7998	5.0787	0.4170	BCC	Arc-melting	227
V0.75Au0.25	4.6108	0.0000	0.7564	4.6753	0.3940	BCC	Quenching from 1400 ℃	105
Mo0.7Cr0.3	4.0146	0.0000	6.0165	5.0787	0.2291	BCC	Pelletized and reacted	140
Cr0.75Mo0.25	4.0878	0.0000	6.0420	4.6753	0.2165	BCC	Arc-melting	224
Cr0.7Mo0.3	4.0797	0.0000	6.3500	5.0787	0.2291	BCC	Pelletized and reacted	140
Cr0.5Mo0.5	4.0472	0.0000	6.7416	5.7628	0.2500	BCC		124
Cr0.5W0.5	3.8078	0.0000	7.5209	5.7628	0.3500	BCC		266
Cr0.75Os0.25	4.5066	0.0000	4.8183	4.6753	0.2338	BCC		282
Cr0.7Re0.3	4.2769	0.0000	5.8411	5.0787	0.1100	BCC		189
Cr0.73Ru0.27	4.7012	0.0000	3.1479	4.8492	0.2397	BCC		189
Cr0.95Ru0.05	4.2346	0.0000	1.5698	1.6505	0.1177	BCC		189
V0.99Hf0.01	3.1579	0.0000	2.1482	0.4656	0.0328	BCC	Arc-melting	230
V0.95Hf0.05	3.1072	0.0000	4.6653	1.6505	0.0719	BCC	Arc-melting	230
Mo0.8Hf0.2	3.5529	0.0000	3.7190	4.1603	0.3440	BCC	Induction-melting	25
Mo0.67Hf0.33	3.2845	0.0000	4.3196	5.2726	0.4044	BCC	Arc-melting	319
W0.67Hf0.33	2.9637	0.0000	3.5631	5.2726	0.4984	BCC	Arc-melting	319
Nb0.78Hf0.22	2.8060	0.0000	2.0692	4.3807	0.1243	BCC	Arc-melting	21
Hf0.5Nb0.5	2.4812	0.0000	2.4631	5.7628	0.1500	BCC	Arc-melting	21
Hf0.7Nb0.3	2.2492	0.0000	2.2354	5.0787	0.1375	BCC	Van Akel process	210
Hf0.5Ta0.5	2.2809	0.0000	1.9608	5.7628	0.1000	BCC		46
Hf0.6Mo0.4	2.7270	0.0000	4.3915	5.5954	0.4213	BCC	Induction-melting	25
Hf0.52Mo0.48	2.8922	0.0000	4.5108	5.7562	0.4297	BCC	Arc-melting	206
Hf0.8Mo0.2	2.3141	0.0000	3.5225	4.1603	0.3440	BCC	Arc-melting	253
Mo0.5Tc0.5	4.5760	0.0000	1.8767	5.7628	0.1300	BCC	Arc-melting	118
Mo0.57Re0.43	4.2485	0.0000	0.5235	5.6811	0.1287	BCC		287
Mo0.6Re0.4	4.2287	0.0000	0.5179	5.5954	0.1274	BCC	Arc-melting	119
Mo0.92Re0.08	4.0184	0.0000	0.2858	2.3177	0.0705	BCC		14
Mo0.62Re0.38	4.2156	0.0000	0.5130	5.5210	0.1262	BCC		7
Mo0.7Ru0.3	4.6509	0.0000	2.9502	5.0787	0.0183	BCC	Arc-melting	130
Mo0.85Pt0.15	4.5753	0.0000	2.4685	3.5144	0.0428	BCC	Arc-melting	112
Mo0.65Rh0.35	5.1568	0.0000	4.4056	5.3829	0.0572	BCC	Arc-melting	228
Mo0.85Rh0.15	4.4762	0.0000	3.2383	3.5144	0.0428	BCC		43
Mo0.86Os0.14	4.2003	0.0000	0.9165	3.3669	0.0139	BCC	Induction-melting	26
Mo0.95Pd0.05	4.1972	0.0000	2.4223	1.6505	0.0087	BCC		43
Mo0.9Ir0.1	4.2406	0.0000	1.5873	2.7027	0.0120	BCC	Arc-melting	252
Cr0.96Nb0.04	4.0858	0.0000	3.7486	1.3963	0.0118	BCC	Arc-melting	129

Mo0.5Nb0.5	3.5135	0.0000	2.0619	5.7628	0.2800	BCC	Arc-melting	129
Nb0.52Mo0.48	3.4954	0.0000	2.0585	5.7562	0.2798	BCC		120
Mo0.516Nb0.484	3.5280	0.0000	2.0622	5.7586	0.2799	BCC	Electron-beam floating-zone melting	292
Nb0.95Mo0.05	3.1065	0.0000	0.8824	1.6505	0.1220	BCC	Arc-melting	4
Nb0.5Tc0.5	4.1237	0.0000	3.9370	5.7628	0.1500	BCC	Arc-melting	53
Nb0.69Tc0.31	3.7200	0.0000	3.5880	5.1472	0.1387	BCC	Electron beam melting	9
Nb0.5V0.5	3.1160	0.0000	7.3171	5.7628	0.0150	BCC	Arc-melting	121
Nb0.5W0.5	3.2742	0.0000	1.2788	5.7628	0.3800	BCC	Arc-melting	21
Nb0.75Re0.25	3.4517	0.0000	2.2149	4.6753	0.1299	BCC	Arc-melting	31
Nb0.56Re0.44	3.7485	0.0000	2.5640	5.7028	0.1489	BCC	Arc-melting	8
Nb0.6Re0.4	3.6860	0.0000	2.5252	5.5954	0.1470	BCC	Arc-melting	31
Nb0.553Re0.447	3.7594	0.0000	2.5690	5.7160	0.1492	BCC		287
Nb0.6Ru0.4	4.3366	0.0000	5.1568	5.5954	0.2939	BCC		86
Nb0.65Ru0.35	4.1772	0.0000	4.9944	5.3829	0.2862	BCC	Arc-melting	114
Nb0.83Os0.17	3.4998	0.0000	2.4941	3.7902	0.2254	BCC	Arc-melting	235
Nb0.98Cr0.02	3.0826	0.0000	2.2700	0.8151	0.0084	BCC	Arc-melting	129
Nb0.88Cr0.12	3.1893	0.0000	5.3558	3.0506	0.0195	BCC		137
Nb0.75Pd0.25	4.4442	0.0000	6.5832	4.6753	0.2598	BCC		29
Nb0.67Pd0.33	4.8867	0.0000	7.2367	5.2726	0.2821	BCC		17
Nb0.8Rh0.2	3.9227	0.0000	5.1813	4.1603	0.2720	BCC	Induction-melting	135
Nb0.815Rh0.185	3.8581	0.0000	5.0200	3.9815	0.2640	BCC	Arc-melting	50
Nb0.93Ir0.07	3.3169	0.0000	2.3344	2.1088	0.1531	BCC		31
Nb0.9Pt0.1	3.5580	0.0000	3.2159	2.7027	0.2040	BCC		234
Cr0.95Ta0.05	4.0551	0.0000	4.4187	1.6505	0.0349	BCC		181
Re0.61Ta0.39	3.8577	0.0000	3.0377	5.5600	0.1951	BCC		287
Mo0.5Ta0.5	3.3132	0.0000	2.5641	5.7628	0.3300	BCC	Electron beam zone refining	178
Mo0.96Ta0.04	3.9136	0.0000	1.0292	1.3963	0.1293	BCC		14
Nb0.5Ta0.5	2.8609	0.0000	0.5025	5.7628	0.0500	BCC		46
Ta0.5V0.5	2.9156	0.0000	7.8167	5.7628	0.0650	BCC		164
V0.67Ta0.33	3.0023	0.0000	7.5517	5.2726	0.0611	BCC	Arc-melting	213
Ta0.5W0.5	3.0738	0.0000	1.7812	5.7628	0.4300	BCC		321
Ta0.6Re0.4	3.4456	0.0000	3.0117	5.5954	0.1960	BCC		286
Ta0.55Re0.45	3.5437	0.0000	3.0678	5.7212	0.1990	BCC	Arc-melting	152
Ta0.75Re0.25	3.1512	0.0000	2.6376	4.6753	0.1732	BCC		7
Ta0.75Ru0.25	3.5578	0.0000	4.8978	4.6753	0.3031	BCC	Arc-melting	95

Ta0.85Cr0.15	2.8808	0.0000	6.2291	3.5144	0.0571	BCC		137
Ta0.88Pd0.12	3.3724	0.0000	5.1324	3.0506	0.2275	BCC		232
Ta0.9Os0.1	2.9586	0.0000	2.2670	2.7027	0.2100	BCC	Rapid cooling from anneal	217
Cr0.86Ti0.14	3.8687	0.0000	2.0728	3.3669	0.0416	BCC	Arc-melting	285
Cr0.5Ti0.5	3.2008	0.0000	2.9240	5.7628	0.0600	BCC		107
Ti0.8Cr0.2	2.6442	0.0000	2.2989	4.1603	0.0480	BCC		209
Mo0.67Ti0.33	3.4072	0.0000	3.5511	5.2726	0.2915	BCC	Arc-melting	319
Mo0.5Ti0.5	3.1195	0.0000	3.8251	5.7628	0.3100	BCC		120
Nb0.5Ti0.5	2.6672	0.0000	5.8824	5.7628	0.0300	BCC		289
Nb0.954Ti0.046	3.0250	0.0000	2.3396	1.5511	0.0126	BCC		3
Ta0.5Ti0.5	2.4668	0.0000	6.3830	5.7628	0.0200	BCC		57
Ti0.5V0.5	2.7219	0.0000	1.4409	5.7628	0.0450	BCC	Arc-melting	270
V0.99Ti0.01	3.1617	0.0000	0.2908	0.4656	0.0090	BCC	Arc-melting	230
V0.95Ti0.05	3.1258	0.0000	0.6363	1.6505	0.0196	BCC	Arc-melting	230
Ti0.5W0.5	2.8801	0.0000	4.6070	5.7628	0.4100	BCC		103
Ti0.75Rh0.25	3.5470	0.0000	0.7412	4.6753	0.3204	BCC		78
Ti0.8Pd0.2	3.5371	0.0000	1.6037	4.1603	0.2640	BCC	Arc-melting	253
Ti0.8Ru0.2	3.0684	0.0000	0.4535	4.1603	0.2640	BCC		78
Cr0.5V0.5	3.6496	0.0000	1.4837	5.7628	0.0150	BCC		311
Mo0.5V0.5	3.5682	0.0000	5.2632	5.7628	0.2650	BCC		102
V0.5W0.5	3.3289	0.0000	6.0440	5.7628	0.3650	BCC		65
V0.7W0.3	3.2656	0.0000	5.6766	5.0787	0.3345	BCC		65
V0.7Tc0.3	3.7753	0.0000	3.1495	5.0787	0.1237	BCC	Arc-melting	36
V0.85Rh0.15	3.8003	0.0000	0.4169	3.5144	0.2321	BCC	Arc-melting	233
V0.8Ir0.2	3.8792	0.0000	2.0833	4.1603	0.2280	BCC	Arc-melting	30
V0.73Os0.27	3.8377	0.0000	3.5561	4.8492	0.2531	BCC		7
V0.8Os0.2	3.6647	0.0000	3.2221	4.1603	0.2280	BCC		40
V0.8Re0.2	3.4611	0.0000	3.8991	4.1603	0.1080	BCC	Arc-melting	153
V0.8Ru0.2	3.7864	0.0000	1.6241	4.1603	0.2280	BCC	Arc-melting	231
V0.75Ru0.25	3.9404	0.0000	1.7546	4.6753	0.2468	BCC		86
V0.95Pt0.05	3.4136	0.0000	0.7634	1.6505	0.1417	BCC		240
Mo0.5W0.5	3.7264	0.0000	0.7833	5.7628	0.1000	BCC		267
Tc0.5W0.5	4.3366	0.0000	2.6596	5.7628	0.2300	BCC		10
W0.85Ru0.15	3.9014	0.0000	2.8079	3.5144	0.0571	BCC		66
W0.75Re0.25	3.7711	0.0000	1.1291	4.6753	0.1992	BCC		287
W0.65Re0.35	3.8847	0.0000	1.2470	5.3829	0.2194	BCC	Arc-melting	119
W0.8Re0.2	3.7143	0.0000	1.0417	4.1603	0.1840	BCC		260

W0.71Re0.29	3.8165	0.0000	1.1844	5.0063	0.2087	BCC		189
W0.95Pd0.05	3.7424	0.0000	2.7272	1.6505	0.0349	BCC	Arc-melting	297
W0.99Ir0.01	3.5193	0.0000	0.6707	0.4656	0.0159	BCC		187
W0.9Os0.1	3.7025	0.0000	1.2487	2.7027	0.0480	BCC	Arc-melting	24
Nb0.999Y0.001	3.0595	0.0000	0.2235	0.0657	0.0120	BCC	Arc-melting	35
W0.97Zr0.03	3.4490	0.0000	1.1467	1.1202	0.1757	BCC		303
V0.99Zr0.01	3.1611	0.0000	2.0324	0.4656	0.0298	BCC	Arc-melting	230
V0.95Zr0.05	3.1230	0.0000	4.4157	1.6505	0.0654	BCC	Arc-melting	230
Mo0.925Zr0.075	3.8347	0.0000	2.2041	2.2147	0.2186	BCC		76
Ta0.82Zr0.18	2.5810	0.0000	1.1464	3.9192	0.0653	BCC	Arc-melting	47
Mo0.67Zr0.33	3.3891	0.0000	3.8526	5.2726	0.3903	BCC	Arc-melting	320
Mo0.53Zr0.47	3.1445	0.0000	4.0429	5.7479	0.4143	BCC	Arc-melting	206
W0.67Zr0.33	3.0684	0.0000	3.0984	5.2726	0.4843	BCC	Arc-melting	319
Zr0.686Nb0.314	2.4830	0.0000	1.8246	5.1735	0.1253	BCC	Arc-melting	116
Nb0.5Zr0.5	2.6398	0.0000	1.9802	5.7628	0.1350	BCC	Arc-melting	5
Ti0.5Zr0.5	2.2457	0.0000	7.8534	5.7628	0.1050	BCC		269
Zr0.65W0.35	2.6624	0.0000	3.0780	5.3829	0.4913	BCC	Arc-melting, splat-quenced	206
Zr0.8Pd0.2	3.4933	0.0000	7.4522	4.1603	0.3480	BCC	Arc-melting	253
Zr0.87Mo0.13	2.4455	0.0000	2.6387	3.2124	0.2791	BCC	Arc-melting, quenched	174
Zr0.96Mo0.04	2.2883	0.0000	1.5268	1.3963	0.1626	BCC	Arc-melting	207
Cu0.99Ag0.01	9.3777	0.0000	1.3705	0.4656	0.0030	FCC	Induction-melting	154
Pt0.940.Ag0.060	8.0830	0.0000	0.4029	1.8870	0.5415	FCC	Melting	208
Ag0.5Au0.5	9.2213	0.0000	2.6549	5.7628	0.3050	FCC	Melting in evacuated quartz tube	179
Ag0.5Pd0.5	9.0521	0.0000	1.1976	5.7628	0.1350	FCC		
Pd0.9Ag0.1	8.6847	0.0000	0.7117	2.7027	0.0810	FCC	Arc-melting	161
Pd0.7Ag0.3	8.8684	0.0000	1.0924	5.0787	0.1237	FCC	Induction-melting	277
Pd0.8Ag0.2	8.7765	0.0000	0.9512	4.1603	0.1080	FCC	Induction-melting	277
Ag0.5Pt0.5	8.7701	0.0000	3.5088	5.7628	0.1750	FCC	Melting, quenching	125
Ag0.75Pt0.25	9.1407	0.0000	3.0929	4.6753	0.1516	FCC		18
Ag0.91Pt0.09	9.3779	0.0000	2.0678	2.5153	0.1002	FCC		18
Ag0.667Pt0.333	9.0177	0.0000	3.3465	5.2901	0.1650	FCC	Melting	208
Ag0.91Sc0.09	8.7735	0.0000	3.2616	2.5153	0.1631	FCC	Arc-melting	158
Ag0.97Cu0.03	9.5073	0.0000	2.0753	1.1202	0.0051	FCC	Induction-melting	154
Ag0.99Y0.01	9.4292	0.0000	2.8262	0.4656	0.0706	FCC	Arc-melting	158
Ag0.98Y0.02	9.3471	0.0000	3.9653	0.8151	0.0994	FCC		314

Au0.55Cr0.45	6.7701	0.0000	2.3357	5.7212	0.4378	FCC		92
Au0.8Cr0.2	7.9708	0.0000	1.8561	4.1603	0.3520	FCC	Arc-melting	104
Au0.5Cr0.5	6.5299	0.0000	2.3529	5.7628	0.4400	FCC	Cosputtering	296
Pd0.9Au0.1	8.6267	0.0000	0.8850	2.7027	0.1020	FCC	Arc-melting	161
Au0.5Pt0.5	8.4801	0.0000	0.8547	5.7628	0.1300	FCC		126
Au0.84V0.16	8.0096	0.0000	0.6338	3.6554	0.3336	FCC		58
Au0.8V0.2	7.7792	0.0000	0.6920	4.1603	0.3640	FCC	Melting, annealing	159
Au0.88Ti0.12	8.1324	0.0000	0.3730	3.0506	0.3250	FCC		216
Au0.925Zr0.075	8.4279	0.0000	4.7781	2.2147	0.3187	FCC		84
Au0.92Sc0.08	8.3219	0.0000	1.5520	2.3177	0.3201	FCC	Arc-melting	212
Au0.99Mo0.01	8.8817	0.0000	0.9141	0.4656	0.0378	FCC		113
Au0.9Ta0.1	8.3043	0.0000	4.4168	2.7027	0.3120	FCC		94
Cu0.75Au0.25	9.2651	0.0000	8.2479	4.6753	0.2771	FCC		59
Cu0.68Au0.32	9.2339	0.0000	8.7683	5.2118	0.2985	FCC	as-cast	132
Au0.5Cu0.5	9.1538	0.0000	9.0909	5.7628	0.3200	FCC		110
Cu0.88Au0.12	9.3229	0.0000	6.3469	3.0506	0.2080	FCC		261
Au0.71Cu0.29	9.0604	0.0000	7.9468	5.0063	0.2904	FCC		261
Cu0.97Au0.03	9.3630	0.0000	3.3914	1.1202	0.1092	FCC		251
Cu0.791Au0.209	9.2833	0.0000	7.8056	4.2620	0.2602	FCC	Heating, quenching	157
Cu0.75Pd0.25	9.1805	0.0000	6.8823	4.6753	0.1299	FCC	Arc-melting	51
Cu0.748Pd0.252	9.1789	0.0000	6.8984	4.6934	0.1302	FCC	Melting	192
Cu0.712Pd0.288	9.1507	0.0000	7.1541	4.9913	0.1358	FCC		1
Cu0.7Pd0.3	9.1413	0.0000	7.2261	5.0787	0.1375	FCC		19
Cu0.54Pd0.46	9.0159	0.0000	7.6657	5.7362	0.1495	FCC		19
Pd0.866Cu0.134	8.6978	0.0000	4.9315	3.2751	0.1022	FCC	Arc-melting	161
Cu0.5Pt0.5	8.7026	0.0000	9.9379	5.7628	0.1900	FCC		18
Cu0.5Rh0.5	8.3725	0.0000	8.8050	5.7628	0.1900	FCC		41
Cu0.77Cr0.23	8.1693	0.0000	5.8983	4.4835	0.1010	FCC	Rf sputtering	52
Cu0.95Ir0.05	9.2432	0.0000	5.1980	1.6505	0.0654	FCC		79
Cu0.95Ti0.05	9.0212	0.0000	4.6102	1.6505	0.0785	FCC	Induction-melting	200
Cu0.92Ti0.08	8.8081	0.0000	5.7025	2.3177	0.0977	FCC	Melting	293
Cu0.96Ti0.04	9.0922	0.0000	4.1539	1.3963	0.0705	FCC		98
Cu0.98Ti0.02	9.2343	0.0000	2.9804	0.8151	0.0504	FCC		202
Ir0.5Pd0.5	7.6532	0.0000	3.1519	5.7628	0.0000	FCC	Induction-melting	90
Ir0.5Pt0.5	7.3712	0.0000	0.8403	5.7628	0.0400	FCC	Electron beam melting	256
Ir0.5Rh0.5	7.0410	0.0000	1.9830	5.7628	0.0400	FCC		257
Ir0.78Mo0.22	6.1090	0.0000	2.2736	4.3807	0.0166	FCC	Arc-melting	252

Ir0.85Mo0.15	6.3013	0.0000	1.9673	3.5144	0.0143	FCC		7
Rh0.9Mo0.1	7.0283	0.0000	2.9193	2.7027	0.0360	FCC	Arc-melting	228
Rh0.946Mo0.054	7.1848	0.0000	2.2093	1.7470	0.0271	FCC		91
Pt0.8Mo0.2	7.2163	0.0000	2.8953	4.1603	0.0480	FCC		100
Pd0.6Mo0.4	6.7420	0.0000	5.7992	5.5954	0.0196	FCC	Powder metallurgical technique	60
Pd0.82Mo0.18	7.7600	0.0000	4.6695	3.9192	0.0154	FCC	Arc-melting	67
Pd0.74Mo0.26	7.3898	0.0000	5.2799	4.7644	0.0175	FCC	Powder metallurgical technique	145
Mo0.85Re0.15	4.0644	0.0000	0.3765	3.5144	0.0928	FCC		37
Pd0.88Nb0.12	7.9291	0.0000	5.4638	3.0506	0.1950	FCC	Arc-melting	67
Pd0.83Nb0.17	7.6525	0.0000	6.2631	3.7902	0.2254	FCC		145
Pd0.82Nb0.18	7.5972	0.0000	6.3950	3.9192	0.2305	FCC	Arc-melting	28
Pd0.92Nb0.08	8.1503	0.0000	4.5923	2.3177	0.1628	FCC	Arc-melting	161
Pt0.82Nb0.18	7.1347	0.0000	4.4628	3.9192	0.2612	FCC	Arc-melting	234
Ir0.9Nb0.1	6.3482	0.0000	2.9703	2.7027	0.1800	FCC		31
Rh0.9Nb0.1	6.9378	0.0000	4.2735	2.7027	0.2040	FCC	Arc-melting	50
Nb0.5Pd0.5	5.8271	0.0000	7.9019	5.7628	0.3000	FCC	Arc-melting	29
Ir0.8Os0.2	6.4990	0.0000	1.1050	4.1603	0.0000	FCC	Arc-melting	218
Pt0.8Os0.2	7.5513	0.0000	1.7917	4.1603	0.0320	FCC	Arc-melting	218
Pd0.5Pt0.5	8.3109	0.0000	2.3121	5.7628	0.0400	FCC	Arc-melting, levitation-melted	146
Pt0.9Hf0.1	7.4161	0.0000	5.1638	2.7027	0.2940	FCC	Arc-melting	215
Rh0.95Cr0.05	7.2066	0.0000	0.8836	1.6505	0.1351	FCC		89
Ir0.8Cr0.2	6.1965	0.0000	3.1603	4.1603	0.2160	FCC		89
Pd0.6Cr0.4	6.8071	0.0000	0.8759	5.5954	0.2645	FCC		88
Pd0.67Cr0.33	7.1196	0.0000	0.8396	5.2726	0.2539	FCC		99
Pd0.7Cr0.3	7.2536	0.0000	0.8178	5.0787	0.2475	FCC	Arc-melting	273
Pd0.75Cr0.25	7.4768	0.0000	0.7721	4.6753	0.2338	FCC	Arc-melting	190
Cr0.5Pt0.5	6.0787	0.0000	3.2070	5.7628	0.3100	FCC	Arc-melting	242
Pt0.8Cr0.2	7.2488	0.0000	2.5172	4.1603	0.2480	FCC	Reduction method	290
Pt0.75Cr0.25	7.0538	0.0000	2.7335	4.6753	0.2685	FCC	Arc-melting	45
Rh0.875Re0.125	7.0254	0.0000	2.8368	3.1325	0.1257	FCC	Arc-melting	186
Pd0.88Re0.12	8.1165	0.0000	3.6048	3.0506	0.0975	FCC	Arc-melting	186
Ir0.73Re0.27	6.1491	0.0000	1.9498	4.8492	0.1332	FCC	Arc-melting	186
Pt0.58Re0.42	6.5985	0.0000	2.9893	5.6559	0.1876	FCC	Powder metallurgical technique	300
Pt0.7Re0.3	7.0072	0.0000	2.7958	5.0787	0.1741	FCC	Arc-melting	218

Pd0.5Rh0.5	7.9807	0.0000	1.1696	5.7628	0.0400	FCC	Arc-melting	96
Pt0.5Rh0.5	7.6987	0.0000	1.1429	5.7628	0.0000	FCC		83
Pd0.9Ru0.1	8.3585	0.0000	1.5892	2.7027	0.0000	FCC	Arc-melting	2
Ir0.63Ru0.37	6.5418	0.0000	0.5387	5.4786	0.0000	FCC	Arc-melting	96
Ir0.7Ru0.3	6.5743	0.0000	0.5109	5.0787	0.0000	FCC		101
Rh0.6Ru0.4	6.9210	0.0000	1.3997	5.5954	0.0392	FCC	Arc-melting	156
Pd0.85Sc0.15	7.5009	0.0000	3.1276	3.5144	0.2999	FCC	Arc-melting	196
Pd0.925Sc0.075	8.0469	0.0000	2.3223	2.2147	0.2212	FCC	Arc-melting	316
Pd0.9Sc0.1	7.8649	0.0000	2.6393	2.7027	0.2520	FCC	Arc-melting	317
Pd0.92Ta0.08	8.1183	0.0000	4.9044	2.3177	0.1899	FCC	Arc-melting	161
Pd0.82Ta0.18	7.5250	0.0000	6.8220	3.9192	0.2689	FCC	Arc-melting	241
Pd0.86Ta0.14	7.7623	0.0000	6.2055	3.3669	0.2429	FCC	Arc-melting	67
Rh0.92Ta0.08	6.9919	0.0000	4.1818	2.3177	0.2116	FCC	Arc-melting	131
Ir0.9Ta0.1	6.3082	0.0000	3.2967	2.7027	0.2100	FCC		294
Rh0.955Tc0.045	7.2704	0.0000	1.1952	1.5258	0.0788	FCC		155
Pd0.75Tc0.25	7.7412	0.0000	3.5143	4.6753	0.1299	FCC		155
Pt0.62Tc0.38	6.9486	0.0000	1.6245	5.5210	0.1844	FCC		155
Pd0.9Ti0.1	7.9609	0.0000	1.2375	2.7027	0.1980	FCC	Arc-melting	161
Pd0.85Ti0.15	7.6449	0.0000	1.4699	3.5144	0.2357	FCC	Arc-melting	151
Rh0.9Ti0.1	6.8590	0.0000	0.5193	2.7027	0.2220	FCC		82
Ir0.84V0.16	6.1466	0.0000	1.8478	3.6554	0.2090	FCC	Arc-melting	30
Rh0.82V0.18	6.6130	0.0000	0.4451	3.9192	0.2497	FCC	Arc-melting	239
Pd0.75V0.25	7.2373	0.0000	0.5109	4.6753	0.2468	FCC		299
Pd0.81V0.19	7.5626	0.0000	0.4632	4.0425	0.2236	FCC	Arc-melting	67
Pd0.9V0.1	8.0506	0.0000	0.3546	2.7027	0.1710	FCC	Arc-melting	161
Pt0.6V0.4	6.0856	0.0000	1.6835	5.5954	0.3184	FCC	Arc-melting	243
Pt0.75V0.25	6.8143	0.0000	1.4804	4.6753	0.2815	FCC		13
Ir0.8W0.2	6.0682	0.0000	2.8478	4.1603	0.0640	FCC		187
Pd0.78W0.22	7.4696	0.0000	5.7046	4.3807	0.0663	FCC	Arc-melting	297
Pd0.6W0.4	6.5505	0.0000	6.5832	5.5954	0.0784	FCC	Arc-melting	133
Pt0.5W0.5	5.7580	0.0000	4.3243	5.7628	0.0400	FCC	Arc-melting	133
Pd0.88Y0.12	7.7181	0.0000	8.0233	3.0506	0.3185	FCC	Arc-melting	144
Pd0.9Y0.1	7.8639	0.0000	7.4437	2.7027	0.2940	FCC	Arc-melting	317
Nb0.81Zr0.19	2.9011	0.0000	1.5730	4.0425	0.1059	FCC	Electro-decomposition	205
Pd0.79Zr0.21	7.2542	0.0000	8.5254	4.2730	0.3544	FCC	Arc-melting	67
Pd0.8Zr0.2	7.3180	0.0000	8.3900	4.1603	0.3480	FCC	Arc-melting	196
Pd0.85Zr0.15	7.6367	0.0000	7.5690	3.5144	0.3107	FCC	Arc-melting	196

Zr0.88Nb0.12	2.3195	0.0000	1.2679	3.0506	0.0877	FCC	Electro-decomposition	205
Ti0.93Ag0.07	2.7798	0.0000	1.6017	2.1088	0.0995	BCC		142
Hf0.98Au0.02	2.0418	0.0000	2.2960	0.8151	0.1736	HCP	Arc-melting	194
Os0.9Cr0.1	5.4898	0.0000	3.1131	2.7027	0.1620	HCP		7
Ru0.6Cr0.4	5.4011	0.0000	3.3942	5.5954	0.2645	HCP		89
Cr0.5Ir0.5	5.4210	0.0000	4.0462	5.7628	0.2700	HCP	Arc-melting	237
Cr0.5Rh0.5	5.7485	0.0000	2.0649	5.7628	0.3100	HCP	Arc-melting	236
Re0.99Hf0.01	4.5959	0.0000	1.0574	0.4656	0.0597	HCP		72
Hf0.5Ti0.5	2.0872	0.0000	8.3333	5.7628	0.1200	HCP		185
Hf0.5Zr0.5	2.0598	0.0000	0.4831	5.7628	0.0150	HCP	Arc-melting	150
Hf0.618Zr0.382	2.0224	0.0000	0.4689	5.5291	0.0146	HCP		222
Hf0.98Zr0.02	1.9075	0.0000	0.1346	0.8151	0.0042	HCP		197
Zr0.57Hf0.43	2.0820	0.0000	0.4787	5.6811	0.0149	HCP	Arc-melting	180
Re0.86Mo0.14	4.5311	0.0000	0.3686	3.3669	0.0902	HCP		7
Re0.92Mo0.08	4.5706	0.0000	0.2884	2.3177	0.0705	HCP		72
Tc0.85Mo0.15	5.0031	0.0000	1.3581	3.5144	0.0928	HCP		155
Tc0.75Mo0.25	4.8810	0.0000	1.6406	4.6753	0.1126	HCP		204
Tc0.82Mo0.18	4.9664	0.0000	1.4595	3.9192	0.0999	HCP	Arc-melting	119
Pd0.54Mo0.46	6.4644	0.0000	5.8582	5.7362	0.0199	HCP	Induction-melting	71
Mo0.5Pd0.5	6.2793	0.0000	5.8496	5.7628	0.0200	HCP	Powder metallurgical technique	60
Os0.55Mo0.45	4.8872	0.0000	1.3284	5.7212	0.0199	HCP		100
Os0.75Mo0.25	5.2223	0.0000	1.1625	4.6753	0.0173	HCP		7
Os0.6Mo0.4	4.9710	0.0000	1.3099	5.5954	0.0196	HCP	Induction-melting	26
Mo0.5Rh0.5	5.6672	0.0000	4.6832	5.7628	0.0600	HCP	Arc-melting	228
Rh0.73Mo0.27	6.4498	0.0000	4.2499	4.8492	0.0533	HCP	Arc-melting	214
Rh0.58Mo0.42	5.9394	0.0000	4.6578	5.6559	0.0592	HCP		43
Mo0.6Ir0.4	5.0649	0.0000	2.6339	5.5954	0.0196	HCP		100
Ir0.5Mo0.5	5.3396	0.0000	2.7027	5.7628	0.0200	HCP	Arc-melting	252
Ru0.97Mo0.03	6.1810	0.0000	1.1477	1.1202	0.0068	HCP	Arc-melting	8
Ru0.59Mo0.41	5.3132	0.0000	3.2265	5.6274	0.0197	HCP		100
Mo0.5Ru0.5	5.1077	0.0000	3.2609	5.7628	0.0200	HCP	Arc-melting	130
Mo0.6Pt0.4	5.5910	0.0000	3.4463	5.5954	0.0588	HCP	Arc-melting	226
Mo0.58Pt0.42	5.6723	0.0000	3.4769	5.6559	0.0592	HCP	Arc-melting	214
Re0.99Nb0.01	4.6075	0.0000	0.5290	0.4656	0.0298	HCP		72
Tc0.94Nb0.06	5.0586	0.0000	1.9371	1.8870	0.0712	HCP	Electron beam melting	9

Ru0.976Nb0.024	6.1730	0.0000	1.7150	0.9413	0.0918	HCP	Arc-melting	8
Ru0.83Nb0.17	5.7075	0.0000	4.1415	3.7902	0.2254	HCP		86
Ru0.71Nb0.29	5.3249	0.0000	4.9376	5.0063	0.2723	HCP	Arc-melting	114
Ru0.75Nb0.25	5.4525	0.0000	4.7324	4.6753	0.2598	HCP	Arc-melting	114
Os0.85Nb0.15	5.2541	0.0000	2.4830	3.5144	0.2142	HCP		7
Os0.8Nb0.2	5.1251	0.0000	2.7719	4.1603	0.2400	HCP	Arc-melting	238
Os0.5Ru0.5	5.9453	0.0000	1.9284	5.7628	0.0000	HCP	Arc-melting	96
Ru0.556Os0.444	5.9794	0.0000	1.9204	5.7106	0.0000	HCP		186
Os0.65Ir0.35	6.0164	0.0000	1.3014	5.3829	0.0000	HCP	Arc-melting	218
Os0.9Pt0.1	5.8799	0.0000	1.3029	2.7027	0.0240	HCP	Arc-melting	218
Os0.5Re0.5	5.1321	0.0000	0.8043	5.7628	0.1500	HCP	Arc-melting	186
Re0.9Os0.1	4.7249	0.0000	0.4795	2.7027	0.0900	HCP		72
Re0.5Ru0.5	5.4363	0.0000	2.7322	5.7628	0.1500	HCP	Arc-melting	73
Re0.9Ru0.1	4.7858	0.0000	1.6043	2.7027	0.0900	HCP		72
Re0.5Tc0.5	4.9046	0.0000	1.3477	5.7628	0.0000	HCP		155
Re0.6Ir0.4	5.4593	0.0000	2.1208	5.5954	0.1470	HCP	Arc-melting	218
Re0.58Pt0.42	6.0536	0.0000	2.9606	5.6559	0.1876	HCP	Powder metallurgical technique	300
Re0.6Pt0.4	5.9854	0.0000	2.9351	5.5954	0.1862	HCP	Arc-melting	218
Ru0.6Ir0.4	6.4351	0.0000	0.5480	5.5954	0.0000	HCP	Arc-melting	218
Ru0.95Rh0.05	6.3055	0.0000	0.6131	1.6505	0.0174	HCP	Arc-melting	8
Rh0.5Ru0.5	6.8091	0.0000	1.4245	5.7628	0.0400	HCP	Arc-melting	156
Ru0.97Pd0.03	6.3198	0.0000	0.8638	1.1202	0.0000	HCP	Arc-melting	8
Ru0.92Pd0.08	6.4370	0.0000	1.3773	2.3177	0.0000	HCP	Arc-melting	2
Hf0.5Sc0.5	1.6072	0.0000	6.1224	5.7628	0.0300	HCP		283
Sc0.5Zr0.5	1.7658	0.0000	5.6410	5.7628	0.0150	HCP		165
Sc0.90Zr0.10	2.0751	0.0000	1.6393	2.7027	0.4080	HCP	Arc-melting, remelted once	278
Re0.99Ta0.01	4.6035	0.0000	0.6347	0.4656	0.0398	HCP		72
Cr0.96Ta0.04	4.0698	0.0000	3.9810	1.3963	0.0314	HCP		20
Os0.8Ta0.2	5.0450	0.0000	3.1915	4.1603	0.2800	HCP	Arc-melting	219
Ru0.8Ta0.2	5.5317	0.0000	4.8246	4.1603	0.2800	HCP	Arc-melting	176
Ru0.79Ta0.21	5.4959	0.0000	4.9068	4.2730	0.2851	HCP		93
Ir0.5Tc0.5	5.9498	0.0000	0.8264	5.7628	0.1500	HCP	Arc-melting	148
Tc0.75Os0.25	5.2998	0.0000	0.4719	4.6753	0.1299	HCP		204
Os0.5Tc0.5	5.4136	0.0000	0.5435	5.7628	0.1500	HCP	Arc-melting	147
Rh0.5Tc0.5	6.2773	0.0000	2.8090	5.7628	0.1900	HCP		204
Ru0.5Tc0.5	5.7178	0.0000	1.3850	5.7628	0.1500	HCP		204

Tc0.75Pt0.25	5.8968	0.0000	1.4314	4.6753	0.1645	HCP		155
Tc0.67Pt0.33	6.1242	0.0000	1.5585	5.2726	0.1787	HCP	Arc-melting	148
Tc0.53Pd0.47	6.7873	0.0000	3.9607	5.7479	0.1497	HCP		155
Tc0.75Pd0.25	6.0378	0.0000	3.3773	4.6753	0.1299	HCP		204
Re0.99Ti0.01	4.5996	0.0000	0.6355	0.4656	0.0358	HCP		72
Ru0.90Ti0.10	6.5177	0.0000	0.6757	2.7027	0.6600	HCP		81
Ti0.89V0.11	2.3718	0.0000	0.8917	2.8809	0.0282	HCP	Arc-melting	177
Ti0.96Ta0.04	2.2886	0.0000	2.6577	1.3963	0.0078	HCP		38
Ti0.987Ta0.013	2.2782	0.0000	1.5419	0.5768	0.0045	HCP		49
Ti0.97Nb0.03	2.2968	0.0000	2.1244	1.1202	0.0102	HCP		38
Ti0.99Ru0.01	2.3129	0.0000	0.1131	0.4656	0.0657	HCP		81
Re0.97V0.03	4.5796	0.0000	1.5467	1.1202	0.0461	HCP		72
Tc0.95V0.05	5.0853	0.0000	1.4338	1.6505	0.0588	HCP	Arc-melting	36
Os0.85V0.15	5.2705	0.0000	2.7332	3.5144	0.2035	HCP		7
Os0.7V0.3	4.8999	0.0000	3.5485	5.0787	0.2612	HCP		82
Os0.6V0.4	4.6529	0.0000	3.8231	5.5954	0.2792	HCP	Arc-melting	40
Ru0.7V0.3	5.3259	0.0000	1.8237	5.0787	0.2612	HCP	Arc-melting	244
Rh0.81W0.19	6.6311	0.0000	4.4378	4.0425	0.0314	HCP	Arc-melting	214
Re0.9W0.1	4.5095	0.0000	0.7958	2.7027	0.1380	HCP	Arc-melting	218
Re0.92W0.08	4.5323	0.0000	0.7200	2.3177	0.1248	HCP		72
Tc0.9W0.1	5.0162	0.0000	1.6304	2.7027	0.1380	HCP		155
Tc0.85W0.15	4.9312	0.0000	1.9353	3.5144	0.1643	HCP	Arc-melting	10
Os0.65W0.35	4.8872	0.0000	2.0318	5.3829	0.0763	HCP	Arc-melting	24
Ru0.6W0.4	5.1445	0.0000	3.9937	5.5954	0.0784	HCP		66
Ir0.5W0.5	5.1003	0.0000	3.4853	5.7628	0.0800	HCP		187
Ir0.77W0.23	5.9714	0.0000	2.9897	4.4835	0.0673	HCP		187
Ru0.95Y0.05	6.0022	0.0000	4.1236	1.6505	0.2136	HCP		220
Sc0.5Y0.5	1.3083	0.0000	7.0707	5.7628	0.0700	HCP		32
Zr0.7Y0.3	1.9439	0.0000	1.3232	5.0787	0.0504	HCP	Arc-melting	249
Y0.6Zr0.4	1.6694	0.0000	1.4024	5.5954	0.0539	HCP	Arc-melting	249
Ru0.995Zr0.005	6.2294	0.0000	1.1086	0.2617	0.0614	HCP	Arc-melting	8
Re0.99Zr0.01	4.5991	0.0000	0.9517	0.4656	0.0567	HCP		72
Ti0.7Zr0.3	2.2567	0.0000	7.4312	5.0787	0.0962	HCP		211
Ti0.92Zr0.08	2.2687	0.0000	4.5621	2.3177	0.0570	HCP		38
Ti0.5Zr0.5	2.2457	0.0000	7.8534	5.7628	0.1050	HCP		27
Ti0.62Zr0.38	2.2523	0.0000	7.7703	5.5210	0.1019	HCP	Arc-melting	262
Zr0.953Nb0.047	2.2580	0.0000	0.8234	1.5762	0.0571	HCP	Arc-melting	116
Tc0.875Hf0.125	4.7755	0.0000	4.4422	3.1325	0.1984	BCC	Arc-melting	147

Mn0.55Re0.45	4.9956	0.0000	7.7576	5.7212	0.1741	BCC	Arc-melting	264
Mn0.65Fe0.35	5.6179	0.0000	1.4975	5.3829	0.1336	BCC		111
Mn0.944Re0.056	5.2625	0.0000	3.8200	1.7943	0.0805	BCC		74
Re0.8Mo0.2	4.4917	0.0000	0.4246	4.1603	0.1040	BCC		70
Re0.77Mo0.23	4.4720	0.0000	0.4466	4.4835	0.1094	BCC		7
Nb0.02Mn0.98	5.2556	0.0000	3.2027	0.8151	0.0070	BCC		69
Tc0.875Nb0.125	4.9205	0.0000	2.6833	3.1325	0.0992	BCC	Arc-melting	147
Tc0.85Nb0.15	4.8674	0.0000	2.8913	3.5144	0.1071	BCC	Arc-melting	53
Tc0.75Nb0.25	4.6549	0.0000	3.4780	4.6753	0.1299	BCC	Electron beam melting	9
Re0.63Nb0.37	4.0452	0.0000	2.5185	5.4786	0.1448	BCC	Arc-melting	214
Re0.75Nb0.25	4.2327	0.0000	2.2730	4.6753	0.1299	BCC	Arc-melting	248
Re0.6Nb0.4	3.9984	0.0000	2.5516	5.5954	0.1470	BCC	Electron beam melting	61
Os0.67Nb0.33	4.7897	0.0000	3.2293	5.2726	0.2821	BCC	Arc-melting	238
Nb0.5Os0.5	4.3512	0.0000	3.3943	5.7628	0.3000	BCC	Electron beam melting	61
Os0.6Nb0.4	4.6092	0.0000	3.3484	5.5954	0.2939	BCC	Arc-melting	5
Nb0.6Pd0.4	5.2739	0.0000	7.6218	5.5954	0.2939	BCC	Electron beam melting	61
Tc0.875Sc0.125	4.7020	0.0000	0.1806	3.1325	0.1786	BCC	Arc-melting	147
Tc0.875Ta0.125	4.8704	0.0000	3.0370	3.1325	0.1323	BCC	Arc-melting	147
Tc0.83Ta0.17	4.7568	0.0000	3.4352	3.7902	0.1503	BCC	Arc-melting	48
Re0.67Ta0.33	3.9755	0.0000	2.9394	5.2726	0.1881	BCC	Arc-melting	214
Re0.75Ta0.25	4.1325	0.0000	2.7205	4.6753	0.1732	BCC		7
Re0.7Ta0.3	4.0344	0.0000	2.8701	5.0787	0.1833	BCC	Arc-melting	152
Re0.6Ta0.4	3.8381	0.0000	3.0492	5.5954	0.1960	BCC		286
Os0.75Ta0.25	4.8960	0.0000	3.4412	4.6753	0.3031	BCC		7
Os0.5Ta0.5	4.1508	0.0000	3.8961	5.7628	0.3500	BCC	Arc-melting	219
Tc0.89Ti0.11	4.8657	0.0000	1.2019	2.8809	0.1126	BCC	Arc-melting	147
Tc0.875Ti0.125	4.8220	0.0000	1.2711	3.1325	0.1191	BCC	Arc-melting	48
Mn0.883V0.117	5.0512	0.0000	1.9820	3.0006	0.0257	BCC	Arc-melting	68
Mn0.9V0.1	5.0875	0.0000	1.8519	2.7027	0.0240	BCC	Arc-melting	68
Re0.75W0.25	4.3391	0.0000	1.1440	4.6753	0.1992	BCC	Arc-melting	308
Tc0.875Zr0.125	4.8151	0.0000	4.0923	3.1325	0.1885	BCC	Arc-melting	147
Pd0.92Lu0.08	7.9837	0.0000	7.5342	2.3177	0.2523	FCC	Arc-melting	317
Pd0.88Lu0.12	7.6792	0.0000	8.9255	3.0506	0.3022	FCC	Arc-melting	172

Table S2. Structural and feature values of single phase ternary alloy and HEAs from experiment. The expandable features $\{n_d^{ex},\ \sigma_d^{ex}\}$ are obtained using the trained feature transformation module. Atomic size difference (δ), configuration entropy (S_c), electronegativity difference (χ_d) are obtained using equation in method section.

Composition	n_d^{ex}	σ_d^{ex}	δ	S_c	χ_d	Phase	Preparation method	Ref
Ag0.25Au0.25Pd0.5	8.907	0.000	1.886	8.644	0.216	FCC	Induction-melting	364
Co0.15Ni0.6Pd0.25	8.272	0.480	5.480	7.796	0.129	FCC	Arc-melting	359
Co0.25Ni0.25Pt0.5	7.935	0.566	8.117	8.644	0.193	FCC	Arc-melting	359
Cr0.125Mn0.125Pt0.75	7.198	0.415	3.443	6.116	0.294	FCC	Arc-melting	332
Cr0.25Co0.25Pt0.5	6.873	0.445	6.114	8.644	0.267	FCC	Arc-melting	343
Cr0.3W0.05Ni0.65	6.859	0.496	7.301	6.576	0.162	FCC		338
Cu0.33Au0.33Ni0.34	8.890	0.175	8.200	9.133	0.299	FCC		336
Cu0.33Ni0.33Pd0.34	8.781	0.101	6.828	9.133	0.140	FCC		337
Fe0.75Ni0.125Pt0.125	6.706	2.181	4.836	6.116	0.147	FCC	Arc-melting	325
Mn0.175Fe0.65Ni0.175	6.429	2.358	2.301	7.400	0.117	FCC	Melting	330
Mn0.33Fe0.33Co0.34	6.283	2.390	2.360	9.133	0.145	FCC	Melted in plasma jet furnace	346
Mn0.3Co0.2Pt0.5	7.065	1.366	6.151	8.561	0.320	FCC	Arc-melting	343
V0.25Co0.375Ni0.375	6.676	0.855	5.765	8.997	0.115	FCC		363
V0.25Co0.375Pd0.375	6.756	0.661	5.304	8.997	0.225	FCC		363
V0.25Ni0.375Pd0.375	7.157	0.252	6.234	8.997	0.223	FCC		363
Fe0.08Ti0.092V0.828	3.331	0.195	2.610	4.804	0.062	FCC	Arc-melting	344
Au0.5Cu0.49Fe0.01	9.122	0.030	9.042	6.170	0.321	FCC	Melting	333
Au0.018Co0.853Fe0.129	7.197	1.788	2.062	3.925	0.090	FCC	Melting	350
Cr0.144Ni0.808Ti0.048	7.473	0.279	5.180	4.964	0.113	FCC		328
Cu0.333Ni0.333Pd0.333	8.774	0.102	6.807	9.133	0.139	FCC		337
Mo0.125Ni0.75V0.125	7.176	0.457	9.207	6.116	0.133	FCC		357
Ni0.375Pd0.375V0.25	7.157	0.252	6.234	8.997	0.223	FCC		363
Fe0.5Mn0.2Ni0.3	6.677	2.033	2.776	8.561	0.129	FCC	Arc-melting	339
Ir0.33Os0.33Ru0.34	6.202	0.000	1.627	9.133	0.000	FCC		331
Au0.2Ir0.55Os0.25	6.889	0.000	2.037	8.291	0.136	FCC		356
Cr0.2Ni0.72W0.08	7.137	0.300	8.233	6.323	0.169	FCC		338
Cr0.23Mn0.08Ni0.69	7.154	0.560	4.788	6.619	0.132	FCC		329
Co0.05Pd0.9Rh0.05	8.307	0.058	2.287	3.279	0.073	FCC	Arc-melting	360

Cr0.27Fe0.47Ni0.26	6.210	1.351	3.979	8.801	0.094	BCC	Arc-melting	358
Cr0.33W0.33Mo0.34	3.862	0.000	6.578	9.133	0.293	BCC		353
Hf0.16Ti0.17W0.67	3.027	0.000	4.786	7.173	0.446	BCC	Arc-melting	365
Hf0.17Ti0.16Mo0.67	3.344	0.000	4.853	7.173	0.356	BCC	Arc-melting	365
Hf0.17Zr0.16Mo0.67	3.335	0.000	4.104	7.173	0.398	BCC	Arc-melting	365
Hf0.17Zr0.16W0.67	3.014	0.000	3.351	7.173	0.492	BCC	Arc-melting	365
Hf0.4Zr0.3Nb0.3	2.344	0.000	2.089	9.053	0.132	BCC	Arc-melting	326
Ta0.33Nb0.33W0.34	3.074	0.000	1.501	9.133	0.386	BCC		340
Ta0.33W0.33Mo0.34	3.377	0.000	2.157	9.133	0.366	BCC		340
Ta0.34Nb0.33Mo0.33	3.224	0.000	2.202	9.133	0.290	BCC		340
Ti0.5V0.45Fe0.05	2.874	0.133	2.633	7.114	0.069	BCC		362
Zr0.16Ti0.17Mo0.67	3.398	0.000	4.531	7.173	0.345	BCC	Arc-melting	365
Zr0.16Ti0.17W0.67	3.078	0.000	4.522	7.173	0.438	BCC	Arc-melting	365
Cr0.2Mo0.4Nb0.4	3.637	0.000	6.241	8.771	0.265	BCC	Arc-melting	341
Cr0.25Fe0.5V0.25	4.929	1.391	4.003	8.644	0.093	BCC		342
Cr0.333Mo0.333V0.334	3.754	0.000	5.883	9.134	0.243	BCC		352
Cr0.4Mo0.32W0.28	3.897	0.000	6.895	9.042	0.301	BCC		355
Fe0.1Ti0.18V0.72	3.313	0.238	3.029	6.447	0.074	BCC	Arc-melting	344
Mo0.333Nb0.333W0.333	3.501	0.000	1.708	9.133	0.322	BCC		340
Fe0.1Ti0.18V0.72	3.313	0.238	3.029	6.447	0.074	BCC	Arc-melting	344
Nb0.2Cr0.2Mo0.6	3.817	0.000	5.809	7.901	0.260	BCC	Arc-melting	341
Mo0.33Re0.33V0.34	3.912	0.000	4.684	9.133	0.217	BCC		334
Nb0.333Re0.333V0.333	3.615	0.000	6.007	9.133	0.135	BCC		334
Nb0.333Ti0.333Zr0.333	2.515	0.000	6.565	9.133	0.116	BCC		361
Mo0.23Ru0.5Pd0.27	6.357	0.000	4.154	8.631	0.017	HCP		345
Mo0.2Ru0.5Rh0.3	6.129	0.000	3.330	8.561	0.045	HCP		345
Mo0.43Rh0.3Pd0.27	6.236	0.000	5.287	8.959	0.051	HCP		345
Ru0.7Rh0.15Pd0.15	6.769	0.000	1.927	6.808	0.029	HCP		345
Co0.4Nb0.3Pt0.3	6.266	0.784	11.079	9.053	0.265	HCP		335
Nb0.125Ni0.75Ti0.125	6.950	0.292	10.946	6.116	0.148	HCP		349
Ir0.43Os0.47Ru0.1	6.163	0.000	1.506	7.882	0.000	HCP		331
Mo0.4Pd0.35Rh 0.25	6.436	0.000	5.296	8.984	0.944	HCP		345
Mo0.25Pd0.4Ru0.35	6.616	0.000	4.650	8.984	0.017	HCP		345
Mo0.25Pd0.25Ru0.5	6.264	0.000	4.175	8.644	0.017	HCP	Induction-melting	347
Mo0.3Rh0.4Ru0.3	6.012	0.000	3.962	9.053	0.051	HCP		345
Pd0.15Rh0.15Ru0.7	6.769	0.000	1.927	6.808	0.029	HCP		345
Ag0.375Au0.375Pd0.25	9.064	0.000	2.304	8.997	0.265	FCC	Induction-melting	364
Pd0.75Ag0.125Au0.125	8.750	0.000	1.337	6.116	0.153	FCC	Induction-melting	364
Pd0.34Ag0.33Au0.33	9.008	0.000	2.164	9.133	0.248	FCC	Induction-melting	364
Ag0.34Au0.33Pd0.33	9.017	0.000	2.179	9.133	0.250	FCC	Induction-melting	364
Ni0.34Cu0.3Pd0.3	8.742	0.163	6.559	9.053	0.135	FCC	Arc-melting	348

Ni0.5Cu0.29Fe0.21	8.212	0.827	2.580	8.591	0.031	FCC	Melting	351
Cu0.75Ni0.17Fe0.08	8.953	0.283	2.154	5.978	0.020	FCC	Melting	351
Pt0.5Co0.25Fe0.25	7.404	1.200	7.001	8.644	0.213	FCC	Induction-melting	364
Mo0.6Cr0.2W0.2	3.903	0.000	5.365	7.901	0.233	FCC		354
Nb0.33W0.33Mo0.34	3.509	0.000	1.706	9.133	0.320	BCC		340
W0.45Mo0.45Os0.1	3.918	0.000	1.265	7.889	0.097	BCC	Arc-melting	327
CoFeNi	7.303	1.355	1.882	9.134	0.033	FCC	Mechanically Alloyed plus Spark Plasma Sintered	410
CoMnNi	6.966	1.752	3.311	9.134	0.163	FCC	As-cast	437
CrFeNi	6.262	0.722	4.443	9.134	0.104	FCC	As-cast, water quenching	437
FeMnNi	6.601	1.741	3.168	9.134	0.154	FCC	As-cast, water quenching	437
HfNbZr	2.506	0.000	2.118	9.134	0.135	BCC	As-cast	379
MoRhRu	5.962	0.000	3.956	9.134	0.050	HCP	As-cast	385
NbTiZr	2.601	0.000	6.561	9.134	0.116	BCC	As-cast	412
CoCrNi	6.619	0.676	4.759	9.134	0.111	FCC	As-cast, water quenching	437
TaNbHfZrTi	2.423	0.000	5.775	13.381	0.118	BCC	As-cast	420
TaNbVTi	2.943	0.000	6.922	11.526	0.051	BCC	As-cast	434
WNbMoTa	3.571	0.000	2.029	11.526	0.364	BCC	As-cast	414
WNbMoTaV	3.566	0.000	5.424	13.381	0.343	BCC	As-cast	414
CoCrFeNi	6.516	0.920	4.121	11.526	0.097	FCC	As-cast	411
CoFeMnNi	6.809	1.339	2.913	11.526	0.143	FCC	As-cast, water quenching	437
CoCrMnNi	6.289	0.848	4.343	11.526	0.150	FCC	As-cast	406
CoCrCu0.5FeNi	6.834	1.009	4.484	13.145	0.095	FCC	As-cast	430
CuNiCoFe	7.828	0.781	2.678	11.526	0.031	FCC	As-cast, Mechanically Alloyed plus Spark Plasma Sintered	410
CuNiCoFeMn	7.322	1.541	3.627	13.381	0.135	FCC	As-cast	390
CuNi2FeMn2Cr	6.724	1.474	4.653	12.885	0.155	FCC	As-cast	368
FeCoNiCrCu	7.088	0.948	4.669	13.381	0.092	FCC	As-cast	383
FeCrMnNiCo	6.273	1.531	3.898	13.381	0.138	FCC	As-cast	406
VCuFeCoNi	6.896	1.057	5.794	13.381	0.104	FCC	As-cast	429
CoCrCuFeNiTi	7.088	0.886	6.743	14.897	0.139	FCC		425
CoCrFeMnNbNi	5.209	1.508	9.982	14.897	0.141	FCC	As-cast	367
Co0.5CrFeMn1.5Ni	6.068	1.753	3.805	12.946	0.146	FCC	As-cast	396
CoCrFeMnNiV	6.273	1.531	3.898	13.381	0.138	FCC	As-cast	367
CoCrFeMo0.85Ni	6.069	1.057	8.820	13.364	0.156	FCC	Additive Manufacturing	419
CoCrFeMoNi	6.006	1.027	9.135	13.381	0.161	FCC	As-cast	400
Co1.5CrFeMo0.5Ni1.5Ti0.5	6.174	1.061	7.706	14.172	0.152	FCC	As-cast	431
CoCrFeNb0.3Ni	6.275	1.228	7.824	12.825	0.109	FCC	As-cast	422

CoCr2FeNi	6.038	0.909	4.470	11.076	0.108	FCC	As-cast	396
CoCrFeNiTi0.5	6.044	1.116	5.558	13.145	0.127	FCC	As-cast	416
Co1.5CrFeNi1.5Ti	6.033	1.096	6.144	13.211	0.138	FCC	As-cast	395
CoCrFeNiW	5.910	1.018	9.784	13.381	0.233	FCC	Mechanically alloying	415
CoCrMnNiV	5.665	1.148	5.182	13.381	0.143	BCC	As-cast	373
CoFeMnMoNi	6.240	1.743	9.133	13.381	0.195	FCC	As-cast	373
CoFeMnNiV	6.081	1.692	4.899	13.381	0.143	FCC	As-cast	373
CoFeNb0.75Ni2V0.5	6.513	0.981	10.442	12.482	0.119	FCC	As-cast	386
CrFe1.5MnNi0.5	5.732	1.872	3.396	10.982	0.131	FCC	As-cast	369
CrHfNbTiZr	2.716	0.000	8.810	13.381	0.145	BCC	As-cast	374
CrNbTiVZr	2.970	0.000	8.570	13.381	0.118	BCC	As-cast	405
CrNbTiZr	2.920	0.000	8.658	11.526	0.124	BCC	As-cast	405
HfNbTiVZr	2.525	0.000	8.026	13.381	0.138	BCC	As-cast	371
Mo0.7NbTiV0.3Zr	2.820	0.000	6.514	12.795	0.272	BCC	As-cast	424
CoCrCuFeMnNi	6.790	1.399	4.588	14.897	0.136	FCC	As-cast	403
CoCuFeMnNi	7.322	1.541	3.627	13.381	0.135	FCC	As-cast	390
CrMnNbTiV	3.587	0.646	7.348	13.381	0.046	BCC	As-cast	384
MoNbTaTiV	3.026	0.000	6.218	13.381	0.241	BCC		404
CrMoNbTaTiZr	3.051	0.000	7.436	14.897	0.257	BCC		427
Ag0.18Cu0.35Au0.37Pd0.10	9.158	0.000	7.843	10.594	0.294	FCC		381
Fe0.698Cr0.195Ni0.093Mn0.014	5.991	0.927	3.016	7.070	0.080	FCC		382
Co0.25Ni0.25Pd0.25V0.25	8.211	0.129	3.527	5.268	0.155	FCC		389
CoFeReRu	6.107	1.214	8.897	11.526	0.145	HCP	As-cast	376
MoPdRhRu	6.544	0.000	4.445	11.526	0.044	HCP	As-cast	385
Co1.5Cr0.5FeMn0.5Ni	6.739	1.452	3.456	12.662	0.118	FCC	As-cast	396
CoCr0.75FeMn0.75Ni	6.446	1.536	3.806	13.302	0.133	FCC	As-cast	396
CoCr1.25FeMn0.25Ni	5.951	0.991	4.199	12.630	0.115	FCC	As-cast	396
CoCrFe0.5Mn0.5Ni1.5	2.845	0.945	4.298	12.662	0.130	FCC	As-cast	396
CoCrFeMnNi	6.273	1.531	3.898	13.381	0.138	FCC	As-cast	406
CoCrFeNiPd	6.931	1.030	4.932	13.381	0.175	FCC	As-cast, cold rolled	398
CoCrFeNiPd2	7.208	0.909	5.090	12.976	0.196	FCC	As-cast, cold rolled	398
HfNbTaZr	2.460	0.000	2.031	11.526	0.123	BCC	As-cast	399
HfNbTiZr	2.363	0.000	6.441	11.526	0.130	BCC	As-cast	432
MoNbTaV	3.215	0.000	6.036	11.526	0.257	BCC	As-cast	433
MoNbTaW	3.294	0.000	2.029	11.526	0.364	BCC	As-cast	414
NbTiVZr	2.681	0.000	7.795	11.526	0.117	BCC	As-cast	424
HfMoNbTiZr	2.684	0.000	5.976	13.381	0.310	BCC	As-cast	420
MoNbTaVW	3.269	0.000	5.424	13.381	0.343	BCC	As-cast	414
MoNbTiVZr	2.938	0.000	6.972	13.381	0.275	BCC	As-cast	424
NbReTaTiV	3.158	0.000	6.191	13.381	0.141	BCC	As-cast	435
NbTaTiVW	2.931	0.000	6.313	13.381	0.320	BCC	As-cast	435

HfNbReTaVW	3.151	0.000	6.013	14.897	0.339	BCC	As-cast	440
MoNbTaTiVW	3.103	0.000	5.770	14.897	0.334	BCC	As-cast	440
Mo0.25Pd0.25Rh0.25Ru0.25	6.544	0.000	4.445	11.526	0.044	HCP		407
CoCrCu0.2FeNi	6.633	0.910	4.296	12.568	0.096	FCC	As-cast	420
CoCrCu0.4FeNi	6.749	0.939	4.429	13.011	0.095	FCC	As-cast	420
CoCrCu2FeMnNi	6.923	1.340	5.044	12.976	0.134	FCC	As-cast	403
CoCrCu0.6FeNi	6.861	0.984	4.532	13.242	0.094	FCC	As-cast	420
CoCrCu0.8FeNi	6.960	0.984	4.610	13.351	0.093	FCC	As-cast	420
CoCrCuFeNiTi0.5	6.905	1.077	4.792	13.145	0.094	FCC	As-cast	425
CoCrCuNi	7.291	0.588	5.167	11.526	0.103	FCC	Mechanically Alloying	372
Co0.25Cr0.25FeMn	5.728	2.508	2.429	9.923	0.138	FCC	As-cast, hot rolled	427
CoCrFe3.5Mn4.5	5.682	2.564	2.416	9.871	0.139	FCC	As-cast, hot rolled	391
CoCrFe4Mn4	5.728	2.508	2.429	9.923	0.138	FCC	As-cast, hot rolled	391
CoCrFe4.5Mn3.5	5.778	2.320	2.432	9.871	0.136	FCC	As-cast, hot rolled	391
CoCr0.4Fe8Mn5.4Ni5.2	6.517	1.090	3.020	10.794	0.143	FCC	As-cast, hot rolled	397
CoCrFe2.7MnNi	6.240	1.768	3.379	12.486	0.123	FCC	As-cast	406
CoCrFe6MnNi	6.234	2.003	2.775	10.206	0.103	FCC	As-cast	406
CoCr1.3FeMnNi0.7	6.000	1.540	3.893	13.229	0.135	FCC	As-cast	366
Co1.4CrFeMnNi	6.329	1.549	3.844	13.295	0.136	FCC	As-cast, cold rolled	366
Co5Cr2Fe40Mn27Ni26	6.517	1.090	3.020	10.794	0.143	FCC	As-cast	436
CoCrFeMnNiV0.25	5.880	1.774	3.538	12.708	0.135	FCC	As-cast	402
CoCr1.5Fe3.5Mn0.5Ni2.5V	6.542	1.477	3.707	11.859	0.105	FCC	As-cast, water quenching	387
CoCrFeMo0.3Ni	6.345	1.241	6.765	12.825	0.127	FCC	As-cast	418
Co42.5Cr12.5Fe20Mo5Ni20	6.730	1.431	5.981	11.782	0.102	FCC	As-cast	400
Co1.5CrFeMo0.1Ni1.5Ti0.5	4.906	1.115	31.970	13.374	0.512	FCC	As-cast	431
CoCr0.5FeNi	6.853	1.204	3.545	11.239	0.081	FCC	As-cast	394
CoCr0.6FeNi	6.780	1.222	3.704	11.358	0.085	FCC	As-cast	394
CoCr0.7FeNi	6.710	1.169	3.836	11.438	0.089	FCC	As-cast	394
CoCr0.8FeNi	6.644	1.104	3.947	11.490	0.092	FCC	As-cast	394
CoCr0.95FeNi	6.552	1.011	4.082	11.524	0.096	FCC	As-cast	394
CoCr0.9FeNi	6.582	1.033	4.041	11.517	0.094	FCC	As-cast	394
CoCr1.05FeNi	6.476	0.877	4.156	11.524	0.098	FCC	As-cast	394
CoCr1.10FeNi	6.449	0.877	4.188	11.518	0.099	FCC	As-cast	394
CoCr1.15FeNi	6.392	0.883	4.218	11.510	0.100	FCC	As-cast	394
CoCrFeNiW0.3	6.320	1.234	7.155	12.825	0.166	FCC	As-cast	409
CoCrMoNbTi0.4	4.551	0.442	9.945	13.011	0.225	BCC	As-cast	441
CoCuFeNiV	6.843	1.057	5.794	13.381	0.104	FCC	As-cast	429
CoCuFeNiTi	6.679	1.054	6.953	13.381	0.139	FCC	As-cast	426
Cr0.5CuFeMn0.5Ni	7.144	1.178	4.667	12.966	0.126	FCC	As-cast	368

CrCu0.5FeMnNi	6.343	1.470	4.597	13.145	0.142	FCC	As-cast	368
CrCuFeMn2Ni2	6.686	1.474	4.653	12.885	0.155	FCC	As-cast	368
CrCu0.7FeNi	6.825	0.930	5.070	11.438	0.102	FCC	As-cast	417
Cr0.66FeMnNi	6.166	1.648	3.851	11.410	0.145	FCC	As-cast, water quenching	438
CrMoNbTaTiV1W1Zr	3.275	0.000	7.332	15.127	0.328	BCC	As-cast	401
CrMoNbTaTiVZr	3.206	0.000	7.781	16.178	0.238	BCC	As-cast	401
Cr0.5MoNbTaVW	3.524	0.000	6.424	14.697	0.332	BCC	As-cast	439
CrMoNbTaVW	3.583	0.000	7.029	14.897	0.321	BCC	As-cast	439
Cr2MoNbTaVW	3.670	0.000	7.665	14.532	0.303	BCC	As-cast	439
HfMoNbTaTi	2.912	0.000	5.575	13.381	0.288	BCC	As-cast	420
HfMoNbTaTiVZr	2.893	0.000	6.933	16.178	0.264	BCC	As-cast	375
HfMo0.25NbTaTiZr	2.617	0.000	5.706	14.335	0.190	BCC	As-cast	388
HfMo0.5NbTaTiZr	2.687	0.000	5.636	14.697	0.232	BCC	As-cast	388
HfMo0.75NbTaTiZr	2.754	0.000	5.567	14.855	0.262	BCC	As-cast	388
HfMoNbTaTiZr	2.813	0.000	5.499	14.897	0.284	BCC	As-cast	388
HfMoNbTaZr	2.911	0.000	3.181	13.381	0.311	BCC	As-cast	420
HfMo0.5NbTiV0.5	2.805	0.000	7.245	12.966	0.250	BCC	As-cast	392
Hf0.5Mo0.5NbTiZr	2.722	0.000	6.224	12.966	0.259	BCC	As-cast	378
HfMo0.5Nb0.5TiZr	2.583	0.000	6.574	12.966	0.270	BCC	As-cast	371
HfMoTaTiZr	2.726	0.000	6.020	13.381	0.311	BCC	As-cast	420
HfMo0.2Ti2V0.5Zr	2.429	0.000	8.265	11.597	0.188	BCC	As-cast	393
HfNbTaTiVZr	2.676	0.000	7.445	14.897	0.126	BCC	As-cast	376
Hf0.5Nb0.5Ta0.5Ti1.5Zr	2.449	0.000	7.094	12.423	0.112	BCC	As-cast	421
Hf0.75NbTa0.5Ti1.5Zr1.25	2.505	0.000	6.647	12.841	0.119	BCC	As-cast	408
Hf0.8Nb0.31Ta0.31Ti0.18Zr0.13	2.413	0.000	4.746	11.663	0.125	BCC	As-cast	408
HfNb0.18Ta0.18Ti1.27Zr	2.283	0.000	7.378	11.437	0.115	BCC	As-cast, cold rolled	408
HfNb0.5Ta0.5TiZr	2.419	0.000	6.451	12.966	0.118	BCC	As-cast	371
HfNbTaTiZr	2.538	0.000	5.775	13.381	0.118	BCC	As-cast, cold rolled	420
HfNb3Ta3TiZr	2.751	0.000	4.319	12.178	0.106	BCC	As-cast	377
HfNb2TiVZr2	2.655	0.000	7.173	12.885	0.137	BCC	As-cast	371
HfNb0.5TiV0.5Zr	2.460	0.000	7.900	12.966	0.135	BCC	As-cast	371
HfTaTiZr	2.372	0.000	6.459	11.526	0.104	BCC	As-cast	371
MoNbTaTiVZr	2.955	0.000	45.102	14.897	0.658	BCC	As-cast	401
MoNbTaTi0.25W	3.433	0.000	3.060	12.708	0.363	BCC	As-cast	380
MoNbTaTi0.5W	3.373	0.000	3.681	13.145	0.362	BCC	As-cast	380
MoNbTaTi0.75W	3.317	0.000	4.112	13.332	0.359	BCC	As-cast	380
MoNbTaTiW	3.266	0.000	4.428	13.381	0.357	BCC	As-cast	380
MoNbTiV	3.178	0.000	5.866	11.526	0.249	BCC	As-cast	370
Mo0.1NbTiV0.3Zr	2.702	0.000	7.054	11.621	0.160	BCC	As-cast	424

Mo0.3NbTiV0.3Zr	2.776	0.000	6.859	12.318	0.214	BCC	As-cast	424
Mo0.3NbTiVZr	2.886	0.000	7.518	12.825	0.197	BCC	As-cast	424
Mo0.5NbTiV0.3Zr	2.871	0.000	6.680	12.648	0.248	BCC	As-cast	424
Mo0.5NbTiVZr	2.944	0.000	7.349	13.145	0.228	BCC	As-cast	424
Mo0.7NbTiVZr	2.995	0.000	7.191	13.308	0.251	BCC	As-cast	424
MoNbTiV0.25Zr	3.013	0.000	6.212	12.708	0.298	BCC	As-cast	428
MoNbTiV0.3Zr	3.032	0.000	6.287	12.825	0.296	BCC	As-cast	424
MoNbTiV0.5Zr	3.040	0.000	6.544	13.145	0.290	BCC	As-cast	428
MoNbTiV0.75Zr	3.050	0.000	6.789	13.332	0.282	BCC	As-cast	428
MoNbTiV1.5Zr	3.076	0.000	7.208	13.254	0.262	BCC	As-cast	428
MoNbTiV2Zr	3.085	0.000	7.330	12.976	0.251	BCC	As-cast	428
MoNbTiV3Zr	3.103	0.000	7.388	12.264	0.232	BCC	As-cast	428
Mo1.3NbTiV0.3Zr	3.089	0.000	6.082	12.724	0.312	BCC	As-cast	424
Mo1.3NbTiVZr	3.123	0.000	6.772	13.330	0.292	BCC	As-cast	424
Mo1.5NbTiV0.3Zr	3.057	0.000	5.956	12.614	0.320	BCC	As-cast	424
Mo1.5NbTiVZr	3.159	0.000	6.647	13.254	0.300	BCC	As-cast	424
Mo1.7NbTiVZr	3.195	0.000	6.530	13.154	0.307	BCC	As-cast	424
Mo2NbTiVZr	3.244	0.000	6.364	12.976	0.314	BCC	As-cast	424
MoNbTiZr	2.996	0.000	5.755	11.526	0.307	BCC	As-cast	424
MoTaTiV	3.132	0.000	6.216	11.526	0.265	BCC	As-cast	401
MoTaTiVZr	2.996	0.000	7.126	13.381	0.281	BCC	As-cast	401
NbTaTiV	2.895	0.000	6.922	11.526	0.051	BCC	As-cast	435
NbTaTiVZr	2.805	0.000	7.349	13.381	0.105	BCC	As-cast	413
NbTaTiZr	2.667	0.000	5.825	11.526	0.100	BCC	As-cast	423
NbTiV0.3Zr	2.655	0.000	7.157	10.836	0.117	BCC	As-cast	424
NbTiV2Zr	2.867	0.000	7.975	11.076	0.113	BCC	As-cast	405

References

1. A. A. Presnyakov, L. I. Dautova and Y. A. Dzhanbusinov Phys. Met. Metallogr. 16, 1, 52-55 (1963).
2. A. A. Rudnitskii and R. S. Polyakova, Russ. J. Inorg. Chem. 4, 631-636 (1959)
3. A. Clauss and B. Heulin C. R. Seances Acad. Sci., Ser. C 291, 215-218 (1980).
4. A. Drake, T. R. Finlayson and I. R. Harris J. Less-Common Met. 129, 123-131 (1987).
5. A. E. Dwight Metall. Soc. Conf. (Proc.). 10, 383-405 (1961).
6. A. Fuchs and B. Jlschner Acta Crystallogr. 19, 488 (1965).
7. A. G. H. Andersen and E. R. Jette Trans. Am. Soc. Met. 24, 375-419 (1936).
8. A. G. Knapton J. Less-Common Met. 1, 480-486 (1959).

9. A. L. Giorgi and E. G. Szklarz J. Less-Common Met. 20, 173-175 (1970).
10. A. L. Giorgi Physica B+C (Amsterdam) 135, 420-422 (1985).
11. A. L. Sutton and W. Hume Rothery Philos. Mag. 46, 1295-1309 (1955).
12. A. M. Van Der Kraan, D. B. De Mooij and K. H. J. Buschow Phys. Status Solidi A 88, 231-237 (1985).
13. A. Maldonado and K. Schubert, Z. Metallkd. 55, 619-626 (1964)
14. A. N. Zelikman et al., Inorg. Mater. 7, 381-384 (1971).
15. A. P. Gulgaev and E. F. Trusova Zh. Tekh. Fiz. 20, 66-78 (1950).
16. A. P. Miodownik J. Magn. Magn. Mater. 10, 126-135 (1979).
17. A. Raman and K. Schubert Z. Metallkd. 55, 798-804 (1964).
18. A. Schneider amd U. Esch Z. Elektrochem. Angew. Phys. Chem. 49, 72-89 (1943).
19. A. Soutter, A. Colson and J. Hertz Mem. Sci. Rev. Metall. 68, 575-591 (1971).
20. A. T. Grigor'ev et al., Russ. J. Inorg. Chem. 5, 1275-1277 (1960)
21. A. Taylor and N. J. Doyle J. Less-Common Met. 13, 413-430 (1967).
22. A. Taylor and R. W. Floyd J. Inst. Met. 80, 577-587 (1951).
23. A. Taylor J. Inst. Met. 77, 585-594 (1950).
24. A. Taylor, B. J. Kagle and N. J. Doyle J. Less-Common Met. 3, 333-347 (1961).
25. A. Taylor, N. J. Doyle and B. J. Kagle J. Less-Common Met. 3, 265-280 (1961).
26. A. Taylor, N. J. Doyle and B. J. Kagle J. Less-Common Met. 4, 436-450 (1962).
27. A. V. Dobromyslov and N. I. Taluts, Phys. Met. Metallogr. 63, 114-120 (1987)
28. B. C. Giessen et al., Metall. Trans. A 11, 709-715 (1980)
29. B. C. Giessen et al., Metall. Trans. A 11, 709-715 (1980).
30. B. C. Giessen, P. N. Dangel and N. J. Grant J. Less-Common Met. 13, 62-70 (1967).
31. B. C. Giessen, R. Nordheim and N. J .Grant Trans. Metall. Soc. AIME 221, 1009-1013 (1961).
32. B. J. Beaudry and A. H. daane, Trans. Metall. Soc. AIME 227, 865-868 (1963)
33. B. V. Cherkashin, Y. A. Vereshchagin, A. A. Kuranov and F. A. Sidorenko Phys. Met. Metallogr. 2, 176-177 (1983).
34. C. C. Chao, P. E. Duwez and C. C. Tsuei J. Appl. Phys. 42, 4282-4284 (1971).
35. C. C. Koch and D. M. Kroeger J. Less-Common Met. 40, 29-38 (1975).
36. C. C. Koch and G. R. Love J. Less-Common Met. 15, 43-58 (1968).
37. C. J. McHargue and H. W. Maynor, Jr. Trans. Am. Inst. Min. Metall. Pet. Eng. 197, 1382-1382 (1953)

38. C. J. McHargue, S. E. Adair, Jr. and J. P. Hammond, Trans. Am. Inst. Min. Metall. Pet. Eng. 197, 1199-1203 (1953)
39. C. Lin, J. B. Liu, G. W. Yang and B. X. Liu J. Alloys Compd. 283, 225-230 (1999).
40. C. P. Susz, R. Flükiger, J. L. Jorda and J. Muller J. Less-Common Met. 63, 45-52 (1979).
41. C. Raub, E. Röschel, D. Menzel and M. Gadhof Metall (Heidelberg). 25, 761-762 (1971).
42. C. S. Smith J. Appl. Phys. 12, 817-822 (1941).
43. C. W. Haworth and W. Hume Rothery J. Inst. Met. 87, 265-272 (1958).
44. Crespo P. et al., J. Magn. Magn. Mater. 150, 409-416 (1995).
45. D. E. G. Williams, A. Jezierski J. Magn. Magn. Mater. 59, 41-56 (1986).
46. D. E. Williams and W. H. Pechin Trans. Am. Soc. Met. 50, 1081-1089 (1958).
47. D. E. Williams, R. J. Jackson and W. L. Larsen Trans. Metall. Soc. AIME 224, 751-756 (1962).
48. D. J. Lam, J. B. Darby, Jr., J. W. Downey and L. J. Norton, Nature 192, 744-744 (1961)
49. D. J. Maykuth , H. R. Ogden and R. I. Jaffee Trans. Am. Inst. Min. Metall. Pet. Eng. 197, 225-230 (1953).
50. D. L. Ritter, B. C. Giessen and N. J. Grant Trans. Metall. Soc. AIME 230, 1250-1259 (1964).
51. D. M. Jones and E. A. Owen Proc. Phys. Soc., London, Sect. B 67, 297-303 (1954).
52. D. McIntyre, J. E. Sundgren and J. E. Greene J. Appl. Phys. 64, 3689-3696 (1988).
53. D. O. Van Ostenburg et al., J. Phys. Soc. Jpn. 18, 1744-1754 (1963).
54. D. P.Morris and J. L. Hughes Acta Crystallogr. 15, 1062 (1962).
55. D. Papantonis and W. A. Bassett J. Appl. Phys. 48, 3374-3378 (1977).
56. D. S. Rickerby Met. Sci. 16, 495-496 (1982).
57. D. Summers Smith J. Inst. Met. 81, 73-76 (1952).
58. D. Summers Smith J. Inst. Met. 83, 189-190 (1954).
59. E. A. Owen and Y. H. Liu Philos. Mag. 38, 354-360 (1947).
60. E. Anderson, J. Less-Common Met. 6, 81-84 (1964)
61. E. Bucher, F. Heiniger and J. Müller, Helv. Phys. Acta 34, 843-858 (1961)
62. E. F. Kneller J. Appl. Phys. 35, 2210-2211 (1964).
63. E. Gebhardt amd W. Köster Z. Metallkd. 32, 253-261 (1940).

64. E. Hornbogen and M. Roth Arch. Eisenhüttenwes. 36, 201-209 (1965).
65. E. I. Gladyshevskii. T. F. Fedorov, R. V. Skolozdra and L. V. Gorshkova Sov. Powder Metall. Met. Ceram. 6, 406-408 (1967).
66. E. J. Rapperport and M. F. Smith Trans. Metall. Soc. AIME 230, 6-11 (1964).
67. E. Kudielka-Artner and B. B. Argent, Proc. Phys. Soc. London 80, 1143-1148 (1962)
68. E. Luqscheider and P. Ettmayer Monatsh. Chem. 102, 1234-1244 (1971).
69. E. M. Savitskii and C. V. Kopetskii, Russ. J. Inorg. Chem. 5, 363-364 (1960)
70. E. M. Savitskii, M. A. Tylkina and K. B. Povarova, Russ. J. Inorg. Chem. 4, 190-195 (1959)
71. E. M. Savitskii, M. A. Tylkina and O. K. Khamidov, Russ. J. Inorg. Chem. 9, 1475-1477 (1964)
72. E. M. Savitskii, M. A. Tylkina and O. K. Khamidov, Russ. Metall. 4, 130-135 (1969)
73. E. M. Savitskii, M. A. Tylkina and V. P. Polyakova, Russ. J. Inorg. Chem. 7, 224-225 (1962)
74. E. M. Savitskii, M. A. Tylkina, R. V. Kirilenko and C. V. Kopetskii, Russ. J. Inorg. Chem. 6, 755-756 (1961)
75. E. P. Abrahamson II and S. L. Lopata Trans. Metall. Soc. AIME 236, 76-87 (1966).
76. E. Pipitz and R. Kieffer Z. Metallkd. 46, 187-194 (1955).
77. E. R. Stevens and O. N. Carlson Metall. Trans. 1, 1267-1271 (1970).
78. E. Raub and E. Röschel Z. Metallkd. 57, 546-551 (1966).
79. E. Raub and E. Röschel Z. Metallkd. 60, 142-144 (1969).
80. E. Raub and E. Röschel Z. Metallkd. 61, 113-115 (1970).
81. E. Raub and E. Röschel, Z. Metallkd. 54, 455-462 (1963)
82. E. Raub and E. Röschel, Z. Metallkd. 57, 546-557 (1966)
83. E. Raub and G. Falkenburg, Z. Metallkd. 55, 392-397 (1964)
84. E. Raub and M. Engel Z. Metallkd. 39, 172-177 (1948).
85. E. Raub and P. Walter Z. Metallkd. 41, 234-238 (1950).
86. E. Raub and W. Fritzsche Z. Metallkd. 54, 317-319 (1963).
87. E. Raub and W. Mahler Z. Metallkd. 46, 282-290 (1955).
88. E. Raub and W. Mahler, Z. Metallkd. 45, 648-650 (1954)
89. E. Raub and W. Mahler, Z. Metallkd. 46, 210-215 (1955)
90. E. Raub J. Less-Common Met. 1, 3-18 (1959).
91. E. Raub Z. Metallkd. 45, 23-30 (1954).

92. E. Raub Z. Metallkd. 51, 290-291 (1960).
93. E. Raub, H .Beeskow and W. Fritzsche, Z. Metallkd. 54, 451-454 (1963)
94. E. Raub, H. Beeskow and D. Menzel Z. Metallkd. 52, 189-193 (1961).
95. E. Raub, H. Beeskow and W. Fritzsche Z. Metallkd. 54, 451-454 (1963).
96. E. Raub, J. Less-Common Met. 1, 3-18 (1959)
97. E. Raub, O. Jr. Loebich and H. Beeskow Z. Metallkd. 55, 367-370 (1964).
98. E. Raub, P. Walter and M. Engel Z. Metallkd. 43, 112-118 (1952).
99. E. Raub, R. Goble and E. Röschel, Z. Metallkd. 58, 567-568 (1967)
100. E. Raub, Z. Metallkd. 45, 23-30 (1954)
101. E. Raub, Z. Metallkd. 55, 316-319 (1964)
102. E. Rudy and F. Benesovsky Planseeber. Pulvermetall. 10, 42-64 (1962).
103. E. Rudy and S. Windisch Trans. Metall. Soc. AIME 242, 953-954 (1968).
104. E. Stolz and K. Schubert Z. Metallkd. 53, 433-444 (1962).
105. E. Z. Kurmaev, V. P. Belash, R. Flükiger and A. Junod Solid State Commun. 16, 1139-1142 (1975).
106. F. Abe, T. Tanabe Z. Metallkd. 76, 420-425 (1985).
107. F. B. Cuff, N. J. Grant and C. F. Floe Trans. Am. Inst. Min. Metall. Pet. Eng. 194, 848-853 (1952).
108. F. Grassat, E. Sabatié, P. Lecocq and A. Michel C. R. Seances Acad. Sci., Ser. C 272, 1315-1317 (1971).
109. F. Hofer J. Solid State Chem. 45, 303-308 (1982).
110. F. Lihl, H. Ebel and W. Baumgartner Z. Metallkd. 62, 42-45 (1971).
111. F. M. Jr. Walters and C. Wells Trans. Am. Soc. Met. 23, 727-750 (1935).
112. Flükiger R. et al., J. Less-Common Met. 32, 207-225 (1973).
113. G. A. Geach and D. Summers Smith J. Inst. Met. 82, 471-474 (1953).
114. G. F. Hurley and J. H. Brophy J. Less-Common Met. 7, 267-277 (1964).
115. G. Hausch and E. Török Phys. Status Solidi A 40, 55-62 (1977).
116. G. M. Benites, A. Fernandez Guillermet, G. J. Cuello and J. Campo J. Alloys Compd. 299, 183-188 (2000).
117. G. Pourroy, S. Lakamp and S. Vilminot J. Alloys Compd. 244, 90-93 (1996).
118. G. R. Stewart and A. L. Giorgi Phys. Rev. B: Condens. Matter 19, 5704-5710 (1979).
119. G. R. Stewart and A. L. Giorgi Solid State Commun. 28, 969-972 (1978).
120. G. S. Upadhyaya Trans. Indian Inst. Met. 20, 53-54 (1967).

121. H. A. Wilhelm, O. N. Carlson and J. M. Dickinson Trans. Am. Inst. Min. Metall. Pet. Eng. 200, 915-918 (1954).
122. H. Asano, Y. Bando, H. Nakanishi and S. Kachi Nippon Kinzoku Gakkaishi 30, 684-688 (1966).
123. H. Chessi, S. Arajs and R. V. Colvin J. Appl. Phys. 35, 2419-2423 (1964).
124. H. D. Kessler and M. Hansen Trans. Am. Soc. Met. 42, 1008-1032 (1950).
125. H. Ebert, J. Abart and J. Voitländer J. Less-Common Met. 91, 89-96 (1983).
126. H. Ebert, J. Abart and J. Voitländer Z. Phys. Chem. (Munich). 144, 223-229 (1985).
127. H. G. Baer Z. Metallkd. 57, 392-395 (1966).
128. H. Iwasaki, K. Okamura and S. Ogawa J. Phys. Soc. Jpn. 31, 497-505 (1971).
129. H. J. Goldschmidt and J. A. Brand J. Less-Common Met. 3, 44-61 (1961).
130. H. Kleykamp J. Less-Common Met. 136, 271-275 (1988).
131. H. Kleykamp, J. Less-Common Met. 152, 15-24 (1989)
132. H. L. Jr. Yakel J. Appl. Phys. 33, 2439-2443 (1962).
133. H. L. Luo, J. Less-Common Met. 15, 299-302 (1968)
134. H. Martens and P. E. Duwez Trans. Am. Soc. Met. 44, 484-494 (1952).
135. H. Oesterreicher amd J. Clinton J. Solid State Chem. 17, 443-445 (1976).
136. H. Ohno J. Phys. Soc. Jpn. 31, 92-101 (1971).
137. H. Rassaerts, F. Benesovsky and H. Nowotny Planseeber. Pulvermetall. 13, 199-206 (1965).
138. H. Sato, K. Taketoshi and R. C. McCune J. Appl. Phys. 43, 324-328 (1972).
139. H. Stuart and N. Ridley J. Phys. D: Appl. Phys. 2, 485-491 (1969).
140. H. T. Tsai and A. Muan J. Am. Ceram. Soc. 75, 1412-1415 (1992).
141. H. Uchishiba J. Phys. Soc. Jpn. 31, 436-440 (1971).
142. H. W. Worner, J. Inst. Met. 82, 222-226 (1953)
143. H. Y. Chen, D. Y. Zhang and G. R. Lu J. Magn. Magn. Mater. 84, 208-212 (1990).
144. I. R. Harris and M. Norman, J. Less-Common Met. 15, 285-298 (1968)
145. J. A. Catterall and S. M. Barker, Met. Space Age: Plansee Proc. 5th, 577-587 (1964)
146. J. B. Darby, Jr. and K. M. Myles, Metall. Trans. 3, 653-657 (1972)
147. J. B. Darby, Jr. J. D. J. Lam, L. J. Norton and J. W. Downey, J. Less-Common Met. 4, 558-563 (1962)
148. J. B. Jr. Darby, L. J. Norton and J. W. Downey J. Less-Common Met. 5, 397-402 (1963).

149. J. C.Woolley, J. H. Phillips and J. A. Clark J. Less-Common Met. 6, 461-471 (1964).
150. J. D. Fast, J. Appl. Phys. 23, 350-351 (1952)
151. J. Evans, I. R. Harris and L. S. Guzei, J. Less-Common Met. 64, 39-57 (1979)
152. J. H. Brophy, P. Schwarzkopf and J. Wulff Trans. Metall. Soc. AIME 218, 910-914 (1960).
153. J. L. Jorda and J. Muller J. Less-Common Met. 119, 337-345 (1986).
154. J. Li, Y. Zhuang and W. Lin J. Alloys Compd. 191, 187-189 (1993).
155. J. Niemiec, Bull. Acad. Pol. Sci. Ser. Sci. Chim. 11, 665-669 (1963)
156. J. O. A. Paschoal, H. Kleykamp and F. Thümmler, J. Less-Common Met. 98, 279-284 (1984)
157. J. W. Brophy and F. X. Kayser Phys. Status Solidi A 30, K33-K36 (1975).
158. K. A. Jr. Gschneidner, O. D. McMasters, D. G. Alexander, and R. F. Venteicher Metall. Trans. 1, 1961-1971 (1970).
159. K. Adachi et al., J. Phys. Soc. Jpn. 48, 62-70 (1980).
160. K. Adachi, K. Sato, M. Matsui and Y. Fujio J. Phys. Soc. Jpn. 30, 1201-1202 (1971).
161. K. Baba et al., J. Mater. Sci. 25, 3910-3916 (1990).
162. K. Baba, Y. Niki, Y. Sakamoto and T. B. Flanagan J. Alloys Compd. 179, 321-331 (1992).
163. K. H. J. Buschow, P. G. Van Engen and R. Jongebreur J. Magn. Magn. Mater. 38, 1-22 (1983).
164. K. H. Richter and A. Weiss Ber. Bunsen-Ges. 92, 833-840 (1988).
165. K. N. Semenenko, V. N. Verbetskii, E. A. Movlaev and A. A. Garibov, Russ. J. Inorg. Chem. 34, 445-446 (1989)
166. K. Schwerdtfeger, L. Zwell Trans. Metall. Soc. AIME 242, 631-633 (1968).
167. K. Sumiyama, H. Ezawa and Y. Nakamura Phys. Status Solidi A 93, 81-86 (1986).
168. K. Sumiyama, H. Ezawa and Y. Nakamura J. Phys. Chem. Solids 48, 255-261 (1987).
169. K. Sumiyama, N. Kataoka and Y. Nakamura Jpn. J. Appl. Phys. 27, 1693-1698 (1988).
170. K. Sumiyama, N. Ohshima and Y. Nakamura Physica Status Solidi A 98, 229-238 (1986).
171. K. Sumiyama, T. Yoshitake and Y. Nakamura J. Phys. Soc. Jpn. 53, 3160-3165 (1984).

172. K. Takao, Y. Sakamoto, T. Araki and H. Kohzuma, J. Alloys Compd. 193, 41-43 (1993)
173. K. Watanabe Trans. Jpn. Inst. Met. 29, 80-84 (1988).
174. K. Yasohama and T. Ogasawara J. Phys. Soc. Jpn. 36, 1349-1355 (1974).
175. L .M. Di and H. Bakker J. Phys.: Condens. Matter 3, 9319-9326 (1991).
176. L. A. Panteleimonov, O. P. Nesterova, K. G. Akhmetzyanov and I. G. Sokolova, Vestn. Mosk. Univ. Ser. 2: Khim 21, 63-68 (1965)
177. L. C. Ming, M. H. Manghnani and K. W. Katahara, Acta Metall. 29, 479-485 (1981)
178. L. I. Van Torne and G. Thomas Acta Metall. 14, 621-635 (1966).
179. L. Karmazin Czech. J. Phys. 19, 634-639 (1969).
180. L. Ming, M. H. Manghnani and K. W. Katahara, J. Appl. Phys. 52, 1332-1335 (1981)
181. L. N. Guseva amd L. B. Mariengof Inorg. Mater. 1, 809-813 (1965).
182. L. N. Larikov and O. A. Shmatko Dopov. Akad. Nauk Ukr. RSR, Ser. A 540-542 (1967).
183. L. R. Bidwell and R. Speiser Acta Crystallogr. 17, 1473-1474 (1964).
184. L. Zwell, G. R. Speich and W. C. Leslie Metall. Trans. 4, 1990-1992 (1973).
185. M. A. Tylkina, A. I. Pekarev and E. M. Savitskii, Russ. J. Inorg. Chem. 4, 1059-1060 (1959)
186. M. A. Tylkina, I. A. Tsyganova and E. M. Savitskii, Russ. J. Inorg. Chem. 7, 990-996 (1962)
187. M. A. Tylkina, V. P. Polyakova and V. S. Shekhtman Russ. J. Inorg. Chem. 8, 1335-1338 (1963).
188. M. De Indian J. Phys. 43, 367-376 (1969).
189. M. Garfinkle Metall. Trans. 1, 1062-1063 (1970).
190. M. H. Rashid and D. J. Sellmyer, J. Appl. Phys. 55, 1735-1737 (1984)
191. M. Hayase, M. Shiga and Y. J. Nakamura Phys. Soc. Jpn. 30, 729-735 (1971).
192. M. Hirabayashi, S. Ogawa, M. Hirabayashi and S. Ogawa J. Phys. Soc. Jpn. 12, 259-271 (1957).
193. M. Ko, T. Nishizawa Nippon Kinzoku Gakkaishi 43, 118-126 (1979).
194. M. Lomello-Tafin, P. Galez, P. Feschotte and J. L. Jorda, J. Alloys Compd. 296, 103-111 (2000)
195. M. Lu and C. L. Chien J. Appl. Phys. 67, 5787-5789 (1990).
196. M. Norman and I. R. Harris, J. Less-Common Met. 18, 333-345 (1969)

197. M. P. Krug and B. E. Davis, J. Less-Common Met. 22, 363-366 (1970)
198. M. S. Wire and G. W. Webb J. Phys. Chem. Solids 42, 233-238 (1981).
199. M. Shiga, M. Miyake and Y. Nakamura J. Phys. Soc. Jpn. 55, 2290-2295 (1986).
200. M. V. Itkin and O. A. Shmatko Phys. Met. 4, 806-810 (1982).
201. N. Honda, Y. Tanji and Y. Nakagawa J. Phys. Soc. Jpn. 41, 1931-1938 (1976).
202. N. Karlsson J. Inst. Met. 79, 391-405 (1951).
203. N. Shen, I. P. Jones and J. N. Pratt Proc. Int. Conf. Rapidly Quenched Met. 4th 1553-1556 (1981).
204. N. Y. Alekseyevskiy, O. A. Balakhovskii and I. V. Kirillov, Phys. Met. Metallogr. 40, 38-42 (1975)
205. O. Jin and B. X. Liu, J. Phys. Condens. Matter 6, L39-L42 (1994)
206. Ö. Rapp J. Less-Common Met. 21, 27-44 (1970).
207. O. S. Ivanov and A. T. Semenchenkov Russ. J. Inorg. Chem. 4, 638-642 (1959).
208. P. Durussel and P. Feschotte J. Alloys Compd. 239, 226-230 (1996).
209. P. E. Duwez and J. L. Taylor Trans. Am. Soc. Met. 44, 495-517 (1952).
210. P. E. Duwez J. Appl. Phys. 22, 1174-1175 (1951).
211. P. E. Duwez, J. Inst. Met. 80, 525-527 (1951)
212. P. E. Rider, K. A. Jr. Gschneidner and O. D. McMasters Trans. Metall. Soc. AIME 233, 1488-1496 (1965).
213. P. F. Schippnick and A. C. Lawson Solid State Commun 15, 1643-1644 (1974).
214. P. Greenfield and P. A. Beck, Trans. Am. Inst. Min. Metall. Pet. Eng. 206, 265-276 (1956)
215. P. J. Meschter and W. L. Worrell, Metall. Trans. A 8, 503-509 (1977)
216. P. Pietrokowsky J. Inst. Met. 90, 434-438 (1961).
217. P. S. Rudman J. Less-Common Met. 9, 77-79 (1965).
218. P. S. Rudman, J. Less-Common Met. 12, 79-81 (1967)
219. P. S. Rudman, J. Less-Common Met. 9, 77-79 (1965)
220. P. Sharifrazi, R. C. Mohanty and A. Raman, Z. Metallkd. 75, 801-805 (1984)
221. Pearson W.B., Hume Rothery W. J. Inst. Met. 80, 641-652 (1951).
222. R. B. Russell, J. Appl. Phys. 24, 232-233 (1953)
223. R. C. Ruhl, B. C. Giessen, M. Cohen and N. J. Grant Mater. Sci. Eng. 2, 314-319 (1967).
224. R. Cywinski and T. J. Hicks J. Magn. Magn. Mater. 54/57, 999-1000 (1986).

225. R. E. Hanneman and A. N. Mariano Trans. Metall. Soc. AIME 230, 937-939 (1964).
226. R. Flükiger et al., J. Less-Common Met. 32, 207-225 (1973)
227. R. Flükiger, C. P. Susz, F. Heiniger and J. Muller J. Less-Common Met. 40, 103-119 (1975).
228. R. Gurler and J.N. Pratt J. Alloys Compd. 177, 321-330 (1991).
229. R. H. Jones, V. F. Zackay and E. R. Parker Metall. Trans. 3, 2835-2842 (1972).
230. R. Kuentzler and H. R. Khan Phys. Lett. A 113, 89-92 (1985).
231. R. M. Waterstart and R. C. Manuszewski J. Less-Common Met. 48, 151-158 (1976).
232. R. M. Waterstart, B. C. Giessen, R. Koch and R. C. Manuszewski Metall. Trans. A 9, 643-648 (1978).
233. R. M. Waterstart, R. C. Manuszewski J. Less-Common Met. 52, 293-305 (1977).
234. R. M. Waterstrat amd B. C. Giessen Metall. Trans. A 16, 1943-1949 (1985).
235. R. M. Waterstrat and R. C. Manuszewski J. Less-Common Met. 51, 55-67 (1977).
236. R. M. Waterstrat and R. C. Manuszewski, J. Less-Common Met. 32, 331-343 (1973)
237. R. M. Waterstrat and R. C. Manuszewski, J. Less-Common Met. 32, 79-89 (1973)
238. R. M. Waterstrat and R. C. Manuszewski, J. Less-Common Met. 51, 55-67 (1977)
239. R. M. Waterstrat and R. C. Manuszewski, J. Less-Common Met. 52, 293-305 (1977)
240. R. M. Waterstrat Metall. Trans. 4, 455-466 (1973).
241. R. M. Waterstrat, B. C. Giessen, R. Koch and R. C. Manuszewski, Metall. Trans. A 9, 643-648 (1978)
242. R. M. Waterstrat, Metall. Trans. 4, 1585-1592 (1973)
243. R. M. Waterstrat, Metall. Trans. 4, 455-466 (1973)
244. R. M. Waterstrat, R. C. Manuszewski, J. Less-Common Met. 48, 151-158 (1976)
245. R. P. Van Ingen, R. H. J. Fastenau and E. J. Mittemeijer J. Appl. Phys. 76, 1871-1883 (1994).
246. R. R. Preston J. Mater. Sci. 1, 309-310 (1966).
247. R. Ray, B. C. Giessen and N. J. Grant Metall. Trans. 3, 627-629 (1972).
248. R. Steadman and P. M. Nuttall, Acta Crystallogr. 17, 62-63 (1964)
249. R. Wang, Metall. Trans. 3, 1213-1221 (1972)
250. S. B. Prima, L. A. Tret'yachenko and G. I. Kostrygina Dopov. Akad. Nauk Ukr. RSR, Ser. A 229-233 (1979).
251. S. C. Ng, B. N. Brockhouse and E. D. Hallman Mater. Res. Bull. 2, 69-73 (1967).
252. S. J. Michalik and J. H. Brophy Trans. Metall. Soc. AIME 227, 1047-1053 (1963).

253. S. J. Poon Solid State Commun. 47, 431-434 (1983).
254. S. L. Zhang, K. Sumiyama and Y. Nakamura J. Magn. Magn. Mater. 73, 58-64 (1988).
255. S. Maki and K. Adachi J. Phys. Soc. Jpn. 46, 1131-1137 (1979).
256. S. N. Tripathi and M. S. Chandrasekharaiah J. Less-Common Met. 91, 251-260 (1983).
257. S. N. Tripathi and M. S. Chandrasekharaiah Z. Metallkd. 74, 241-245 (1983).
258. S. Ochiai, Y. Mishima and T. Suzuki Bull. Res. Lab. Precis. Mach. Electron. (Tokyo Inst. Technol.). 53, 15-28 (1984).
259. S. Ohara, S. Komura and T. Takeda Magnetic J. Phys. Soc. Jpn. 34,1472-1476 (1973).
260. S. S. Budagovskiy, V. N. Bykov, M. I. Gavrylyuk and V. N. Pod'yachev Metallofizika (Akad. Nauk Ukr. SSR, Inst. Metallofiz.) 44, 57-67 (1973).
261. S. S. Lu and C. K. Liang Chin. J. Phys. Peking 22, 505-527 (1966).
262. S. S. Sidhu, L. Heaton and M. H. Mueller, J. Appl. Phys. 30, 1323-1340 (1959)
263. S. Setz, H. Nowotny and F. Benesovsky Monatsh. Chem. 99, 2004-2015 (1968).
264. S. Setz, H. Nowotny and F. Benesovsky, Monatsh. Chem. 99, 730-732 (1968)
265. S. U. Jen and S. A. Chang J. Appl. Phys. 73, 6402-6404 (1993).
266. S. V. Nagender Naidu, A. M. Sriramamurthy and P. R. Rao Trans. Indian Inst. Met. 36, 24-29 (1983).
267. S. V. Nagender Naidu, A. M. Sriramamurthy and P. R. Rao Trans. Indian Inst. Met. 37, 107-110 (1984).
268. T. A. Yamaoka J. Phys. Soc. Jpn. 36, 445-450 (1974).
269. T. Doi, H. Ishida and T. Umezawa Nippon Kinzoku Gakkaishi 30, 139-145 (1966).
270. T. Hagii, Y. Sato, M. Yasuda and K. Tanaka Trans. Jpn. Inst. Met. 28, 198-204 (1987).
271. T. I. Babyuk, G. P. Kushta, O. I. Rybailo Izv. Vyssh. Uchebn. Zaved., Chern. Metall. 7, 126-128 (1974).
272. T. J. Quinn and W. Hume Rothery J. Less-Common Met. 5, 314-324 (1963).
273. T. Kaneko and H. Fujimori, J. Phys. Soc. Jpn. 28, 1373-1373 (1970)
274. T. Onozuka, S. Yamaguchi, M. Hirabayashi and T. Wakiyama J. Phys. Soc. Jpn. 37, 687-693 (1974).
275. T. Suzuki and M. Hagiwara Trans. Jpn. Inst. Met. 16, 473-479 (1975).
276. T. Takahashi, W. A. Bassett and H. K. Mao J. Geophys. Res. 73, 4717-4725 (1968).

277. T. Tsuchida J. Phys. Soc. Jpn. 18, 1016-1019 (1963).
278. T. W. E. Tsang, K. A. Gschneidner, Jr. and F. A. Schmidt, Phys. Rev. B 21, 3100-3109 (1980)
279. U. Esch and A. Schneider Z. Elektrochem. Angew. Phys. Chem. 50, 268-274 (1944).
280. V. D. Dobrovolskii, S. M. Karal'nik and A. V. Koval' Metallofizika (Akad. Nauk Ukr. SSR, Inst. Metallofiz.). 41, 73-77 (1972).
281. V. I. Spitsyn S. P. Grishina, O. A. Balakhovskii and A. L. Krasovskiy Russ. Metall. 6, 156-158 (1975).
282. V. N. Svechnikov, G. P. Dmitrieva, G. F. Kobzenko and A. K. Shurin Dokl. Akad. Nauk SSSR 158, 668-670 (1964).
283. V. N. Verbetskii and E. A. Movlaev, Russ. J. Inorg. Chem. 36, 783-784 (1991)
284. V. V. Pet'kov and M. V. Kireev Metallofizika (Akad. Nauk Ukr. SSR, Inst. Metallofiz.). 33, 107-115 (1971).
285. V. V. Pet'kov, V. P. Skvorchuk and S. Y. Golub Russ. Metall. 4,194-198 (1976).
286. V. V. Vavilova et al., Dokl. Phys. Chem. 300, 531-535 (1988).
287. V. V. Vavilova, L. N. Galkin and M. V. Glazov Inorg. Mater. 27, 1801-1805 (1991).
288. W. B. Pearson and W. J. Hume Rothery Inst. Met. 81, 311-314 (1952).
289. W. Baden and A. Weiss Z. Metallkd. 74, 89-93 (1983).
290. W. Bronger and W. Klemm, Z. Anorg. Allg. Chem. 319, 58-81 (1962)
291. W. C. Ellis and E. S. Greiner Trans. Am. Soc. Met. 29, 415-434 (1941).
292. W. C. Hubbell and F. R. Brotzen J. Appl. Phys. 43, 3306-3312 (1972).
293. W. E. Krull amd R. W. Newman J. Appl. Crystallogr. 3, 519-521 (1970)
294. W. H. Ferguson, Jr., B. C. Giessen and N. J. Grant, Trans. Metall. Soc. AIME 227, 1401-1406 (1963)
295. W. Jr. Klement Trans. Metall. Soc. AIME 233, 1180-1182 (1965).
296. W. Jr. Sluserk, B. Lalevic and N. Fuschillo J. Appl. Phys. 44, 2891-2892 (1973).
297. W. K. Goetz and J. H. Brophy J. Less-Common Met. 6, 345-353 (1964).
298. W. Köster amd E. Horn Z. Metallkd. 43, 444-449 (1952).
299. W. Köster and W. Gmöhling, Z. Metallkd. 51, 385-391 (1960)
300. W. Trzebiatowski and J. Berak, Bull. Acad. Pol. Sci. 2, 37-40 (1954)
301. W. Zarek, M. Tuszynski and E. S. Popiel J. Magn. Magn. Mater. 104/107, 2067-2068 (1992).
302. X. Zhou, H. R. Khan and C. J. Raub J. Less-Common Met. 96, 249-256 (1984).

303. Y. A. Chang J. Less-Common Met. 17, 325-328 (1969).
304. Y. A. Dorofeyev, A. Z. Men'shikov and G. A. Takzey Phys. Met. Metallogr. 55, 5, 102-109 (1983).
305. Y. Aoki and M. Yamamoto Phys. Status Solidi A 22, K131-K133 (1974).
306. Y. Aoki and M. Yamamoto Phys. Status Solidi A 33, 625-632 (1976).
307. Y. Aoki, Y. Gotoh and Y. Obi Phys. Status Solidi A 36, K149-K152 (1976).
308. Y. B. Kuz'ma, V. I. Lakh, B. I. Stadnyk and Y. V. Voroshilov, Sov. Powder Metall. Met. Ceram. 7, 462-466 (1968)
309. Y. Endoh and Y. Ishikawa J. Phys. Soc. Jpn. 30, 1614-1627 (1971).
310. Y. Hamaguchi and N. Kunitomi J. Phys. Soc. Jpn. 19, 1849-1856 (1964).
311. Y. I. Dutchak and V. G. Chekh Phys. Met. 4, 169-174 (1982).
312. Y. Matsuo J. Phys. Soc. Jpn. 32, 972-978 (1972).
313. Y. Mishima, S. Ochiai and T. Suzuki Acta Metall. 33, 1161-1169 (1985).
314. Y. Ning, X. Zhou and H. Dai Chin. J. Met. Sci. Technol. 7, 391-398 (1991).
315. Y. Ohtani and I. Hatakeyama J. Appl. Phys. 74, 3328-3332 (1993).
316. Y. Sakamoto, F. L. Chen and R. A. McNicholl, J. Alloys Compd. 192, 145-148 (1993)
317. Y. Sakamoto, F. L. Chen, M. Kinoshita and M. Minamikawa, J. Alloys Compd. 192, 141-144 (1993)
318. Y. V. Pal'guyev, A. A. Kuranov, P. N. Syutkin and F. A. Sidorenko Phys. Met. Metallogr. 42, 1, 46-50 (1976).
319. Z. Blazina, R. Trojko and Z. Ban J. Less-Common Met. 83, 175-183 (1982).
320. Z. Blazina, R. Trojko and Z. Ban J. Less-Common Met. 97, 91-98 (1984).
321. Z. I. Tolmacheva and V. I. Kornilova Russ. Metall. 3, 167-169 (1972).
322. Z. J. Zhang and B. X. Liu J. Appl. Phys. 75, 4948-4952 (1994).
323. Z. J. Zhang and B. X. Liu J. Appl. Phys. 75, 4948-4952 (1994).
324. Z. J. Zhang and B. X. Liu J. Phys.: Condens. Matter 7, L293-L298 (1995).
325. A. P. Miodownik, J. Magn. Magn. Mater. 10, 126-135 (1979)
326. A. Taylor and N. J. Doyle, J. Less-Common Met. 7, 37-53 (1964)
327. A. Taylor and N. J. Doyle, J. Less-Common Met. 9, 190-205 (1965)
328. A. Taylor and R. W. Floyd, J. Inst. Met. 80, 577-587 (1951)
329. A. V. Progrushchenko and Yu. M. Lebedev, Ukr. Fiz. Zh. 14, 282-286 (1969)
330. A. Z. Menshikov, V. A. Kazantsev, N. N. Kuzmin and S. K. Sidorov, J. Magn. Magn. Mater. 1, 91-97 (1975)

331. D. C. Harris and L. J. Cabri, Canad. Mineral. 12, 104-112 (1973)
332. D. E. G. Williams and A. Jezierski, J. Magn. Magn. Mater. 59, 41-56 (1986)
333. D. K.-H. Finkler et al., Physica B+C 145, 335-341 (1987)
334. E. A. Smol'yaninova, E. K. Stribuk and V. I. Tyavlovskii, Izv. Akad. Nauk SSSR Metally 1987, 207-209 (1987)
335. E. A. Statnova, V. V. Kuprina and E. M. Sokolovskaya, Vestn. Mosk. Univ, Ser 2: Khim. 16, 115-116 (1975)
336. E. Raub and A. Engel, Z. Metallkd. 38, 11-16 (1947)
337. E. Raub, O. Loebich, Jr., W. Plate and H. Krill, Z. Metallkd. 62, 826-829 (1971)
338. F. Abe and T. Tanabe, Z. Metallkd. 76, 420-425 (1985)
339. F. J. J. Van Loo, G. F. Bastin, J .W. G. A. Vrolijk and J. J. M. Hendriks, J. Less-Common Met. 72, 225-230 (1980)
340. H. Bückle, Z. Metallkd. 37, 53-56 (1946)
341. H. J. Goldschmidt and J. A. Brand, J. Less-Common Met. 3, 44-61 (1961)
342. I. I. Kornilov, Zh. Neorg. Khim. 2, 355-366 (1957)
343. J. C. Woolley, J. H. Phillips and J. A. Clark, J. Less-Common Met. 6, 461-471 (1964)
344. J. F. Lynch, A. J. Maeland and G. G. Libowitz, Z. Phys. Chem. 145, 51-59 (1985)
345. J. O. A. Paschoal, H. Kleykamp and F. Thümmler, Z. Metallkd. 74, 652-664 (1983)
346. K. Adachi, K. Sato, M. Matsui and Y. Fujio, J. Phys. Soc. Jpn. 30, 1201-1202 (1971)
347. K. Naito, T. Tsuji, T. Matsui and A. Date, J. Nucl. Mater. 154, 3-13 (1988)
348. K. V. K. Rao, J. Less-Common Met. 9, 70-73 (1965)
349. L. I. Pryakhina et al., Poroshk. Metall. 6, 61-69 (1966)
350. L. Lynch, G. Krauss and P. S. Venkatesan, Metall. Trans. 1, 1471-1472 (1970)
351. M. E. Hargreaves, Acta Crystallogr. 4, 301-309 (1951)
352. N. N. Sirota and B. P. Shimanskii, Dokl. Akad. Nauk BSSR 20, 1068-1071 (1976)
353. N. V. Grum-Grzhimailo and D. I. Prokof'ev, Russ. J. Inorg. Chem. 6, 590-595 (1961)
354. N. V. Grum-Grzhimailo and D. I. Prokof'ev, Russ. J. Inorg. Chem. 7, 303-308 (1962)
355. N. V. Grum-Grzhimailo and D. I. Prokof'ev, Zh. Neorg. Khim. 6, 1155-1164 (1961)
356. O. S. Zvyagintsev, Comptes Rendus de l'Academie des Sciences de I'URSS 4, 176-179 (1934)
357. S. B. Prima, L. O. Tret'yachenko and G. I. Kostrigina, Dop. Akad. Nauk Ukr. RSR\A\ 41, 229-233 (1979)
358. S. Ohara, S. Komura and T. Takeda, J. Phys. Soc. Jpn. 34, 1472-1476 (1973)

359. S. U. Jen and Y. R. Huang, J. Appl. Phys. 69, 4674-4676 (1991)
360. T. B. Flanagan, D. Wang, J. D. Clewley and H. Noh, J. Alloys Compd. 305, 172-182 (2000)
361. T. Doi, H. Ishida and T. Umezawa, Nippon Kinzoku Gakkaishi 30, 139-145 (1966)
362. V. N. Verbetskii, S. V. Mitrokhin and K. N. Semenenko, Russ. J. Inorg. Chem. 28, 262-265 (1983)
363. W. Köster and W. Gmöhling, Z. Metallkd. 51, 385-391 (1960)
364. Y. C. Venudhar, L. Iyengar and K. V. K. Rao, J. Less-Common Met. 116, 341-350 (1986)
365. Z. Blazina, R. Trojko and Z. Ban, J. Less-Common Met. 83, 175-183 (1982)
366. A.J. Zaddach, R.O. Scattergood, C.C. Koch. Mat. Sci. Eng. A 636, 373-378 (2015).
367. B. Cantor, I. T. H. Chang, P. Knight, A. J. B. Vincent. Mater. Sci. Eng. A 375, 213-218 (2004).
368. B. Ren, Z.X. Liu, D.M. Li, L. Shi, B. Cai, M.X. Wang. J. Alloys Compd. 493 148-153 (2010).
369. C.-P. Lee, C.-C. Chang, Y.-Y. Chen, J.-W. Yeh, H.-C. Shih. Corros. Sci. 50, 2053-2060 (2008).
370. Chen, S. Y., Yang, X., Dahmen, K. A., Liaw, P. K. & Zhang, Y. Entropy 16, 870–884 (2014).
371. Chen, Y. et al. Materials (Basel). 11 (2018).
372. Durga, A., Hari Kumar, K. C. & Murty, B. S. Trans. Indian Inst. Met. 65, 375–380 (2012).
373. F. Otto, Y. Yang, H. Bei, E.P. George. Acta Mater. 61, 2628-2638 (2013).
374. Fazakas, É. et al. RMHM 47, 131–138 (2014).
375. Gao, M. C. et al. JOM 67, 2653– 2669 (2015).
376. Gao, M. C., Zhang, B., Guo, S. M., Qiao, J. W. & Hawk, A Phys. Metall. Mater. Sci. 47, 3322–3332 (2016).
377. Guo, J. et al. Proc. Natl. Acad. Sci. 114, 13144–13147 (2017).
378. Guo, N. N. et al. Intermetallics 69, 74–77 (2016).
379. Guo, W. et al. A Phys. Metall. Mater. Sci. 44, 1994–1997 (2013).
380. Han, Z. D. et al. Mater. Sci. Eng. A 712, 380–385 (2018).
381. Venudhar Y.C., Reddy C.V.V., Murthy K.S.N., Iyengar L., Krishna Rao K.V. J. Less-Common Met.,109, L25-L28 (1985).

382. Almasan C., Datta T., Edge R.D., Jones E.R., Cable J.W., Ledbetter H.M. J. Magn. Magn. Mater.80, 329-338 (1989).
383. J.-W. Yeh, S.-K. Chen, S.-J. Lin, J.-Y. Gan, T.-S. Chin, T.-T. Shun, C.-H. Tsau, S.-Y. Chang. Adv. Eng. Mat. 6, 299-303 (2004).
384. J.M. Cotton, Kaufman, (2014).
385. J.O.A. Paschoal, H. Kleykamp, F. Z. Metallkd. 74 652 (1983).
386. Jiang, L., Lu, Y., Wu, W., Cao, Z. & Li, T. J. Mater. Sci. Technol. 32, 245–250 (2016).
387. Jo, Y. H. et al. Nat. Commun. 8, 1–8 (2017).
388. Juan, C. et al. Mater. Lett. 175, 284–287 (2016).
389. Köster W., Gmöhling W. Z. Metallkd. 51, 385-391 (1960).
390. L. Jiang, Y. Lu, Y. Dong, T. Wang, Z. Cao, T. Li. Intermetallics 44, 37-43 (2014).
391. Li, Z., Pradeep, K. G., Deng, Y., Raabe, D. & Tasan, C. C. Nature 534, 227–30 (2016).
392. Liu, Y. et al. J. Alloys Compd. 694, 869– 876 (2017).
393. Lu, Y. et al. J. Mater. Sci. Technol. 35, 369–373 (2019).
394. Lucas, M. S. et al. J. Appl. Phys. 113, 1–4 (2013).
395. M.-H. Chuang, M.-H. Tsai, W.-R. Wang, S.-J. Lin, J.-W. Yeh. Acta Mater. 59, 6308-6317 (2011).
396. M.-H. Tsai, K.-Y. Tsai, C.-W. Tsai, C. Lee, C.-C. Juan, J.-W. Yeh. Mater. Res. Lett. 1, 207-212 (2013).
397. M.J. Yao, K.G. Pradeep, C.C. Tasan, D. Raabe. Scripta Mater. 72, 5-8 (2014).
398. M.S. Lucas, L. Mauger, J.A. Munoz, Y.M. Xiao, A.O. Sheets, S.L. Semiatin, J. Horwath, Z. Turgut. J. Appl. Phys. 109 (2011).
399. Maiti, S. & Steurer, W. Acta Mater. 106, 87–97 (2016).
400. Ming, K., Bi, X. & Wang, J. Scr. Mater. 137, 88–93 (2017).
401. Mu, Y. et al. J. Alloys Compd. 714, 668–680 (2017).
402. N.D. Stepanov, D.G. Shaysultanov, G.A. Salishchev, M.A. Tikhonovsky, E.E. Oleynik, A.S. Tortika, O.N. Senkov. J. Alloys Compd. 628, 170-185 (2015).
403. Nagase, T. Mater. Sci. Forum 941, 1238–1241 (2018).
404. O.N. Senkov, G.B. Wilks, D.B. Miracle, C.P. Chuang, P.K. Liaw. Intermetallics 18, 1758-1765 (2010).

405. O.N. Senkov, S.V. Senkova, C. Woodward, D.B. Miracle. Acta Mater. 61, 1545-1557 (2013).
406. Ondicho, I. et al. J. Alloys Compd. 785, 320–327 (2019).
407. Paschoal J.O.A., Kleykamp H., Thümmler F. Z. Metallkd.74, 652-664 (1983).
408. Podolskiy, A. V et al. Mater. Sci. Eng. A 710, 136–141 (2018).
409. Poletti, M. G., Fiore, G., Gili, F., Mangherini, D. & Battezzati, L. Mater. Des. 115, 247–254 (2017).
410. S. Praveen, B.S. Murty, R.S. Mater. Sci. Eng. A 534 83-89 (2012).
411. Salishchev, G. A. et al. J. Alloys Compd. 591, 11–21 (2014).
412. Senkov, O. N., Rao, S., Chaput, K. J. & Woodward, C. Acta Mater. 151, 201–215 (2018).
413. Senkov, O. N., Rao, S., Chaput, K. J. & Woodward, C. Acta Mater. 151, 201–215 (2018).
414. Senkov, O. N., Wilks, G. B., Miracle, D. B., Chuang, C. P. & Liaw, P. K. Intermetallics 18, 1758–1765 (2010).
415. Shang, C. et al. Mater. Des. 117, 193–202 (2017).
416. Shun, T. T., Chang, L. Y. & Shiu, M. H. Mater. Sci. Eng. A 556, 170–174 (2012).
417. Singh, A. K. & Subramaniam, A. Adv. Mater. Res. 585, 3–7 (2012).
418. T.-T. Shun, C.-H. Hung, C.-F. Lee. J. Alloys Compd. 493, 105-109 (2010).
419. T.-T. Shun, L.-Y. Chang, M.-H. Shiu, Mater. Charact. 70, 63-67 (2012).
420. Tseng, K. K. et al. Entropy 21, 1–14 (2019).
421. Veselý, J. et al. J. Appl. Phys. 120, 164902 (2016).
422. W.H. Liu, J.Y. He, H.L. Huang, H. Wang, Z.P. Lu, C.T. Liu. Intermetallics 60, 1-8 (2015).
423. Wang, R. et al. Mater. Des. 162, 256–262 (2019).
424. Wu, Y. D. et al. Mater. Des. 83, 651–660 (2015).
425. X. F. Wang, Y. Zhang, Y. Qiao, G. L. Chen. Intermetallics 15, 357-362 (2007).
426. X. Wang, H. Xie, L. Jia, Z.L. Lu. Mater. Sci. Forum 724, 335-338 (2012).
427. Y. Deng, C.C. Tasan, K.G. Pradeep, H. Springer, A. Kostka, D. Raabe. Acta Mater. 94 124-133 (2015).
428. Y. Zhang, X. Yang, P. K. Liaw. JOM 64 830-838 (2012).
429. Y. Zhang, Y.J. Zhou, J.P. Lin, G.L. Chen, P.K. Liaw. Adv. Eng. Mat. 10, 534-538 (2008).

430. Y.-J. Hsu, W.-C. Chiang, J.-K. Wu. Mater. Chem. Phys. 92 112-117 (2005).
431. Y.-L. Chou, J.-W. Yeh, H.-C. Shih. Corros. Sci. 52, 2571-2581 (2010).
432. Y.D. Wu, Y.H. Cai, T. Wang, J.J. Si, J. Zhu, Y.D. Wang, X.D. Hui. Mater. Lett. 130, 277-280 (2014).
433. Yao, H. et al. Entropy 18, 1–15 (2016).
434. Yao, H. W. et al. NbTaV-(Ti,W) Mater. Sci. Eng. A 674, 203–211 (2016).
435. Yao, H. W. et al. NbTaV-(Ti,W) Mater. Sci. Eng. A 674, 203–211 (2016).
436. Yao, M. J., Pradeep, K. G., Tasan, C. C. & Raabe, D. Scr. Mater. 72, 5–8 (2014).
437. Z. Wu, H. Bei, F. Otto, G.M. Pharr, E.P. George. Intermetallics 46 131-140 (2014).
438. Z. Wu, H. Bei. Mat. Sci. Eng. A 640, 217-224 (2015).
439. Zhang, B., Gao, M. C., Zhang, Y. & Guo, S. M. Calphad Comput. Coupling Phase Diagrams Thermochem. 51, 193–201 (2015).
440. Zhang, B., Gao, M. C., Zhang, Y., Yang, S. & Guo, S. M. Mater. Sci. Technol. 31, 1207–1213 (2015).
441. Zhang, M., Zhou, X. & Li, J. J. Mater. Eng. Perform. 26, 3657–3665 (2017).
